# Genetic analysis of global faba bean diversity, agronomic traits and selection signatures

**DOI:** 10.1007/s00122-023-04360-8

**Published:** 2023-04-19

**Authors:** Cathrine Kiel Skovbjerg, Deepti Angra, Tom Robertson-Shersby-Harvie, Jonathan Kreplak, Gabriel Keeble-Gagnère, Sukhjiwan Kaur, Wolfgang Ecke, Alex Windhorst, Linda Kærgaard Nielsen, Andrea Schiemann, Jens Knudsen, Natalia Gutierrez, Vasiliki Tagkouli, Lavinia Ioana Fechete, Luc Janss, Jens Stougaard, Ahmed Warsame, Sheila Alves, Hamid Khazaei, Wolfgang Link, Ana Maria Torres, Donal Martin O’Sullivan, Stig Uggerhøj Andersen

**Affiliations:** 1 Department of Molecular Biology and Genetics, Aarhus University, 8000 Aarhus, Denmark; 2 Center for Quantitative Genetics and Genomics, Aarhus University, 8000 Aarhus, Denmark; 3 School of Agriculture, Policy and Development, University of Reading, Reading, UK; 4 Agroécologie, AgroSup Dijon, INRAE, Univ. Bourgogne, Univ. Bourgogne Franche-Comté, Dijon, France; 5 Agriculture Victoria, AgriBio, Centre for AgriBioscience, Bundoora, VIC Australia; 6 Department of Crop Sciences, Georg-August-University, Göttingen, Germany; 7 Georg-August-Universität Göttingen, DNPW, Carl-Sprengel 1, Germany; 8 Sejet Planteforædling, 8700 Horsens, Denmark; 9 Nordic Seed, 8300 Odder, Denmark; 10 Área de Mejora Vegetal y Biotecnología, IFAPA Centro “Alameda del Obispo”, Apdo 3092, 14080 Córdoba, Spain; 11 Crops Research, Teagasc, Oak Park, Carlow, Ireland; 12 Production Systems, Natural Resources Institute Finland (Luke), Latokartanonkaari 9, 00790 Helsinki, Finland

## Abstract

**Key message:**

We identified marker-trait associations for key faba bean agronomic traits and genomic signatures of selection within a global germplasm collection.

**Abstract:**

Faba bean (*Vicia faba* L.) is a high-protein grain legume crop with great potential for sustainable protein production. However, little is known about the genetics underlying trait diversity. In this study, we used 21,345 high-quality SNP markers to genetically characterize 2678 faba bean genotypes. We performed genome-wide association studies of key agronomic traits using a seven-parent-MAGIC population and detected 238 significant marker-trait associations linked to 12 traits of agronomic importance. Sixty-five of these were stable across multiple environments. Using a non-redundant diversity panel of 685 accessions from 52 countries, we identified three subpopulations differentiated by geographical origin and 33 genomic regions subjected to strong diversifying selection between subpopulations. We found that SNP markers associated with the differentiation of northern and southern accessions explained a significant proportion of agronomic trait variance in the seven-parent-MAGIC population, suggesting that some of these traits were targets of selection during breeding. Our findings point to genomic regions associated with important agronomic traits and selection, facilitating faba bean genomics-based breeding.

**Supplementary Information:**

The online version contains supplementary material available at 10.1007/s00122-023-04360-8.

## Introduction

Faba bean (*Vicia faba* L*.*) is an important cool-season grain legume (pulse) crop grown worldwide for its high seed-protein content that is of great interest for the production of animal feed and food for human consumption. Faba bean has a high yield potential and an average crude protein content of 29%. In addition, it is one of the most efficient nitrogen fixers and is grown with little or no applied inorganic nitrogen fertilizer (Singh et al. 2013; Griffiths and Lawes 1978; Baddeley et al. 2013). This provides major benefits for cropping systems and supports sustainable agricultural practices.

In 2020, the total worldwide production of faba bean was 5.7 million tonnes, which represents an increase of approximately 55% since 2000 (FAOSTAT 2022). Despite the multiple advantages of growing faba bean, the global production is still surpassed by other pulses such as common bean (*Phaseolus vulgaris* L.), chickpea (*Cicer arietinum* L.), field pea (*Pisum sativum* L.), cowpea (*Vigna unguiculata* L.), and lentil (*Lens culinaris* Medik.) (Adhikari et al. 2021).

In general, faba bean thrives in the cool and moist conditions found in temperate climates, but it is cultivated in various climate zones from boreal to subtropical and warm temperate areas, where it is grown as a winter crop (Singh et al. 2013; O’Sullivan and Angra 2016). Its history of cultivation has been traced back to the Stone Age, making faba bean one of the earliest domesticated crops (Duc et al. 2010). The Middle East is popularly considered the center of origin, although other studies point toward Central Asia (Cubero 1974; Ladizinsky 1975). Interestingly, no wild faba bean progenitor has been found, and *Vicia faba* is not cross-compatible with other *Vicia* species, meaning that all existing faba bean genetic diversity is maintained in germplasm collections and in local populations kept by farmers (Duc et al. 2010). This situation, combined with the current lack of effective transgenic technologies for faba bean, means that ongoing breeding programs rely highly on the exploitation of existing genetic diversity.

For optimal crop improvement, it is crucial to obtain a better understanding of population structure and genetic diversity in the accessible faba bean germplasm. To date, the genetic relationships and diversity of faba bean germplasm have been examined in various studies using different types of molecular markers and germplasm collections (e.g., Torres et al. 1993; Link et al. 1995; Terzopoulos and Bebeli 2008; Oliveira et al. 2016; Kaur et al. 2014a; Sallam et al. 2016a, b; Wang et al. 2012; Mulugeta et al. 2021). Although these studies have found genetic distinctions between germplasm belonging to different geographic origins, the underlying selection signatures remain poorly understood. This is mainly due to the large and complex genome of faba bean (approx. 13 Gbp) (Khazaei et al. 2021).

The identification of genomic regions differentiated between subpopulations of faba bean with different geographic origins will be an important factor in addressing the challenges associated with the frequent climatic fluctuations and future climate change. Signatures of selection have been identified in multiple important crops such as maize (*Zea mays* L.), rice (*Oryza sativa* L.), alfalfa (*Medicago sativa* L.), and soybean (*Glycine max* (L.) Merr.) by comparing subgroups with different geographical origins (Xu et al. 2022; Bouchet et al. 2013; Xie et al. 2015; Chen et al. 2021; Saleem et al. 2021). This is typically done using statistical methods that rely on differences in allele frequencies between subpopulations (Luu et al. 2017; Chen et al. 2010; Tajima 1989; Foll and Gaggiotti 2008). Meanwhile, genome-wide association studies (GWAS) have consistently proven to be a powerful tool for detecting candidate genes for agronomically important traits (Huang et al. 2010; Sonah et al. 2015). By looking for overlaps of genomic regions under selection and quantitative trait loci (QTLs) identified by GWAS studies, it is possible to study traits under selection. However, traits under selection during breeding are typically strongly correlated with population structure, posing a challenge to GWAS. GWAS models correcting for population structure will cause many false negatives, and ultimately QTLs associated with traits under selection might not be identified. In contrast, a naïve GWAS model with no population structure adjustment will yield too many false-positive signals, since it is not able to distinguish genetic regions associated with overall population structure from causal genes associated with traits under selection (Zhao et al. 2011). A way to overcome this problem is to combine selection signatures of a diversity panel with GWAS results from independent populations. This is especially straightforward for well-studied crops such as maize and rice, where a large number of functional genes and loci associated with traits have already been identified and published (Xu et al. 2022; Xie et al. 2015). For an orphan crop such as faba bean, however, most QTLs associated with agronomic important traits have yet to be identified (Adhikari et al. 2021).

In view of the above, the objectives of the present study were to: (1) analyze the genetic diversity, population structure, and linkage disequilibrium (LD) of a global faba bean panel of 2678 accessions using high-quality SNP data; (2) use a mapping population to identify markers associated with key agronomic traits; and (3) select a large, non-redundant diversity panel to study faba bean genetic diversity and inter-population selection signatures. Understanding the genetic diversity and structure of these accessions lays a foundation for future genome-wide association studies (GWAS) or genomic selection (GS) and will aid in the utilization of these materials in future faba bean breeding programs.

## Materials and methods

### Plant materials and panels

The data studied consist of 2682 faba bean accessions belonging to eight different international panels developed in different projects. The panels are referred to, respectively, as: EUCLEG, Göttingen Winter Bean (GWB), four-way-cross, seven-parent-MAGIC, Northern Faba (NORFAB), ProFaba, Reading Spring Bean Panel (RSBP) and Virtual Irish Centre for Crop Improvement (VICCI). The panels differed in size, type, allelic diversity included, and crossing strategies (Table 1). Extensive descriptions of the panels and passport information on the individual accessions can be found in Supplementary File 1.

**Table 1:** Summary of panels

	EUCLEG	NORFAB	ProFaba	GWB	RSBP	VICCI	Seven-parent-MAGIC	Four-way-cross
Number of accessions^a^	358 (358)	195 (196)	234 (234)	268 (268)	160 (162)	563 (564)	255 (255)	645 (645)
Population type	Diversity	Diversity	Diversity	Mapping	Mapping	Outcrossing	Mapping	Mapping
Number of founders	–	–	–	11	21	22	7	4
Number of polymorphic markers	21,286	21,254	21,335	20,722	19,328	20,681	17,352	14,893
Number of markers with MAF ≥ 5%	19,509	19,599	19,630	16,840	16,888	17,307	16,228	13,822

^a^The number of accessions after each panel was filtered for genetic redundancy. The numbers in parentheses refer to the number of accessions before the individual panels were filtered for genetic redundancy

### Phenotyping and field trials of seven-parent-MAGIC lines

The seven-parent-MAGIC lines were grown in field trials at two locations in Denmark during 2020 and 2021. The first location was Sejet Plant Breeding, Sejet (55.82°N, 9.94°E) and the second Nordic Seed, Dyngby (55.96°N, 10.25°E). The experimental design for field trials was an alpha design with three replicates. Plants were grown in plots made up of 6 rows with 14–15 seeds sown per row. Each plot contained two entries and therefore consisted of two inbred lines, each contributing three rows. To minimize neighbor effects, every other plot between the 6-rowed plots consisted of commercial cultivars; Kontu at both field trials during 2020 (Sej20 and Dyn20) and Taifun and Daisy at both field trials during 2021 (Sej21 and Dyn21). For the field trials at Sejet, the sowing dates were 9 April 2020 and 19 April 2021, and the harvest dates were 10–12 September 2020 and 31 August to 1 September 2021. For field trials at Dyngby, the sowing dates were 2 April 2020 and 8 April 2021, and the harvest dates were 24 August 2020 and 21 August 2021.

Trials were rain-fed and treated with herbicides and insecticides. Furthermore, the field trials in Dyngby had fertilizer (NPK 0-8-23) applied. More details on the treatments can be seen in Supplementary Table 1.

To minimize border effects, all traits were scored in the middle row of the three rows per inbred line. Plants were phenotyped for the following 17 traits: disease susceptibility to chocolate spot (caused by *Botrytis fabae*), rust (caused by *Uromyces viciae-fabae*), and downy mildew (caused by *Peronospora viciae*); herbicide damage; branching; plant height; number of ovules per plant; sterile tillers per plant; lodging; maturation date; earliness, end, and duration of flowering; thousand grain weight (TGW); and seed area, length, and width. The description of each trait and scoring methods appears in Supplementary File 2.

### DNA extraction and SNP genotyping

Genomic DNA was extracted from fresh leaf tissue using a DNeasy Plant Mini Kit (QIAGEN Ltd, UK) for the EUCLEG panel, a NucleoSpin Plant II kit (Macherey–Nagel) for the seven-parent-MAGIC and NORFAB panels, and a DNeasy 96 Plant Kit (QIAGEN Ltd, UK) for the remaining panels. DNA quality was assessed on agarose gel electrophoresis, while concentration was assessed using a Quant-iT PicoGreen dsDNA Assay Kit (ThermoFisher Scientific, UK) following the manufacturer’s guidelines.

Individuals were genotyped for SNPs using the Vfaba_v2 Axiom SNP array containing approximately 60 K probes (Khazaei et al. 2021; O’Sullivan et al. 2019). Genotype data were filtered following the ‘best practices workflow’ from the Affymetrix Axiom Analysis Suite which excluded markers with a call rate < 97%. Further, only markers that the software classified as “PolyHighResolution” (high quality and polymorphic) were kept. The flanking sequences of the resulting 24,599 SNPs were aligned to the *Vicia faba* reference sequence (Jayakodi et al. 2022) using the blastn application of the NCBI BLAST + suite of programs (v2.12.0+) with an e-value of 1e−8 as the significance threshold. The significance threshold for the blast analysis had been determined by aligning the first 1000 flanking sequences to the reference genome without a threshold, selecting the best alignments per sequence from the results by maximal bit-score and taking the highest e-value among the best hits rounded to the next higher full figure as significance threshold. The SNP position in the reference sequence was determined based on the “Blast trace-back operations” (BTOP) string of the alignments, counting upwards from subject start to the SNP position in the query sequence when the alignment was on the plus strand and downwards when it was on the minus strand. Markers that did not align to a unique chromosomal position in the genome were removed. This gave a set of 21,345 high-quality quality markers. Analyses were performed on this set of markers, unless otherwise specified.

Note that chromosome 1 was split into two parts (Chr1S and Chr1L) at position 1,574,527,093 by the faba bean genome consortium to facilitate data analysis.

Functional annotation was done using eggNOG-mapper v. 2.7.2 with the eggNOG eukaryotic database (Buchfink et al. 2015; Huerta-Cepas et al. 2017, 2019).

Using the 8423 markers for which both a genetic and physical position (Supplementary File 3, https://projects.au.dk/fabagenome/genomics-data) were available, we modeled, in the R package *cobs*, genetic position as a smooth, strongly monotonic function of physical position, and then, using this function, we estimated genetic positions for all SNPs (Ng and Maechler 2007). These genetic positions were used for imputation of missing genotypes using Beagle v. 5.2 with windows of 60 cM and 3 cM steps (Browning et al. 2018). Prior to this imputation, all markers and individuals showed missingness < 5% and < 8%, respectively.

### Redundancy filtering

Genetic identities (GI) between accessions were calculated as the fraction of shared alleles by applying the following equation to the VCF file using a custom R-script (1):

1 $${\text{GI}}_{{ij}} = \frac{1}{{2n}}\sum\limits_{{x = 1}}^{n} {S_{{xij}} }$$

where GI_*ij*_ is the genetic identity between the *i*th and *j*th sample, *n* is the number of markers where none of the two samples show missingness, *S*_*xij*_ is the number of shared alleles between sample *i* and *j* at marker *x* and therefore takes values of 0, 1, and 2.

When two samples showed GI ≥ 94%, the sample with the largest proportion of genetic missingness or the least information in terms of geographic origin (diversity panel) was removed. The threshold was set so that we excluded most accessions that we knew were present in duplicates and avoided discarding too many genetically close lines.

### Genetic variation and diversity

The site-frequency spectrums were based on the panel-wise polymorphic SNPs (Table 1). The alternative allele counts and resulting plots were made using a custom R-script. Nucleotide diversity was calculated by applying “-site-pi” in VCFtools v. 0.1.16 (Danecek et al. 2011). Observed and expected levels of heterozygosity were calculated in R using the inbreedR and adegenet packages, respectively (Stoffel et al. 2016; Jombart 2008). SNP densities were calculated chromosome wise using ‘–SNP density’ with a distance of 1 M base pairs (bp) in VCFtools v. 0.1.16 (Danecek et al. 2011).

### Population structure and phylogeny

To infer population structure and phylogeny of the diversity panel, a minor allele frequency (MAF) filter at 1% was applied, leaving 21,116 markers. The software ADMIXTURE was run with *K* ranging from 2 to 20 (Alexander et al. 2009). A tenfold cross-validation (CV) scheme was repeated 10 times for each value of *K*. The admixture proportions were graphically displayed using R.

Principal component analysis (PCA) was performed on all accessions across panels and within the diversity panel using the markers that passed a minor allele frequency (MAF) threshold ≥ 1%, that is 21,077 and 21,116 markers, respectively. All PCAs in this study were made by using PLINK v. 1.9 setting the number of principal components (PCs) to the number of samples (Purcell et al. 2007). The resulting eigenvectors were plotted in R using ggplot2 (Wickham 2016). Accessions were assigned to a subpopulation if they showed ancestry proportions ≥ 0.50.

A phylogenetic tree was constructed using MEGA X v. 10.2.6 to generate a neighbor-joining tree, applying a bootstrap method with 1000 replications and default parameters (Kumar et al. 2018). The tree was visualized with the R-package ggtree (Yu et al. 2017).

Population differentiations were investigated by calculating fixation indices (*F*_ST_) between pairs of subpopulations as identified by ADMIXTURE. For this purpose, the ‘–weir-fst-pop’ in VCFtools v. 0.1.16 was used (Danecek et al. 2011). Additionally, analysis of molecular variance (AMOVA) was performed using the adegenet package in R (Jombart 2008). Both *F*_ST_ and AMOVA analyses were based on markers that passed a MAF filter at 1%.

Statistical significance of differential allele frequencies between pairwise populations was calculated using a Fisher’s exact test in R.

### Linkage disequilibrium

Linkage disequilibrium (LD) was estimated individually for each panel using PLINK v. 1.9 to compute the squared correlation coefficients (*R*^2^) chromosome-wise for each pairwise combination of markers (Purcell et al. 2007). Before LD calculations, a MAF filter was applied at 5% in individual panels and 1% in the diversity panel. For each panel, the resulting LD data were merged across chromosomes, subsequently sorted according to SNP distance, and binned into groups of 1000 data points. For each bin, the average *R*^2^ was plotted against the average distance (bp) and a smooth curve was fitted using the *loess* function with a 10% smoothing span in R. There were 5000 bins plotted for the seven-parent-MAGIC and four-way-cross populations where the LD decayed slowly, whereas 1000 bins were plotted for the remaining populations. The LD decay was estimated per panel as the point where the fitted curve reached half of its maximum value.

### Identification of SNPs under selection

To detect SNPs showing signatures of selection, we employed three methods of outlier detection that differ in their statistical approaches. All aim to identify extreme differences in allele frequency between populations. We used the software package Ohana with the number of ancestry components set to 3 (Cheng et al. 2022), the R-package pcadapt with *K* = 3 (Luu et al. 2017) and the software BayeScan v. 2.1 with default settings (Foll and Gaggiotti 2008). In contrast to Ohana and pcadapt, BayeScan requires grouping into populations. For this purpose, we used the population memberships assigned by ADMIXTURE.

For candidate markers under selection, we focused on markers found by at least two of the methods. All methods were applied to the markers that passed a MAF filter at 1%.

### Statistical models and genome-wide association studies

Prior to GWAS, the phenotype scores were filtered for outliers and lines with many off-types were also discarded. This left between 188 and 234 seven-parent-MAGIC accessions for GWAS.

Phenotypic data analyses were performed for each trait in individual field trials and for all environments (envs) combined, using the lme4 package in R (Bates et al. 2015). For analysis of variance (ANOVA) and to get adjusted genotype means for GWAS inputs, we fitted the following mixed model to all traits (Eq. 2):

2 $${y}_{ijk}= \mu +{G}_{i}+{E}_{j}+{G}_{i} x{ E}_{j}+{R(E)}_{jk}+{\varepsilon }_{ijk}$$

where *y*_*ijk*_ denotes the phenotypic value of the *i*th inbred line in the *j*th environment (year x location combination) in the *k*th replication, *µ* is the overall trait mean, *G*_*i*_ is the genetic effect of the *i*th line, *E*_*j*_ is the environmental effect of the *j*th environment, *G*_*i*_* × E*_*j*_ denotes the genotype environment interaction of the *i*th line in the *j*th environment, *R(E)*_*jk*_ is the effect of the *k*th replicate within the *j*th environment, and ${\epsilon }_{ijk}$ is the residual error. All effects except the overall mean were treated as random. If a trait was scored on two separate dates within a field trial, each date was modeled as a separate environment. All random effects in the model were tested one at a time for statistical significance by using the ‘ANOVA’ function in R to compare the log-likelihood of a model with and without the random effect. When testing the significance of main effects, the interaction effects were excluded from the full model before the main effect was dropped. If the removal of an effect was associated with a *p* value > 0.05, inclusion of the effect was not considered to improve the model. To extract best linear unbiased estimators (BLUEs) for each trait, statistically insignificant terms are excluded from Eq. 2, which was then refitted with genotypes as a fixed effect. For the trait x environment combinations where *G*_*i*_ and ${\epsilon }_{ijk}$ were the only significant effects, the average phenotype value of each genotype was used for GWAS.

Broad sense heritabilities were calculated on a line mean basis from the estimated variances of Eq. 2 (Eq. 3):

3 $${H}^{2}= \frac{{\sigma }_{G}^{2}}{{\sigma }_{G}^{2}+ s \frac{{\sigma }_{GE}^{2}}{{n}_{E}}+\frac{{\sigma }_{\varepsilon }^{2}}{{n}_{E}{ n}_{R}}}$$

where ${\sigma }_{G}^{2}$, ${\sigma }_{GE}^{2}$, and ${\sigma }_{\varepsilon }^{2}$ are the estimated variances of the genetic effects, genotype x environment interactions and residual effects, respectively; *n*_E_ and *n*_R_ are the number of environments and replications, respectively; and *s* is a constant taking the value 0 if only one environment is included in Eq. 2 and otherwise taking the value 1. It should be noted that when *s* = 0, *H*^2^ is strictly speaking a measure of line repeatability and not line heritability.

GWAS was performed on the generated BLUEs using the fixed and random model Circulating Probability Unification (FarmCPU) method integrated in the GAPIT v. 3 library in R with a MAF filter of 5% (Liu et al. 2016; Wang and Zhang 2021). To avoid signals originating from population stratification, the first three PCs were included as covariates in the GWAS models. Finally, we verified the absence of confounding effects by checking for inflation of *p* values by examination of *Q*–*Q* plots and calculation of genomic inflation factors ($\lambda$). For traits where $\lambda$-values were not between 0.9 and 1.1, *p* values were divided by $\lambda$. To avoid the high penalty of Bonferroni correction, which assumes all markers are uncorrelated, we calculated the effective number of independent tests (*M*_ef_) using the SimpleM method (Gao et al. 2010). The significance threshold was then estimated as 0.05/*M*_ef_ with *M*_ef_ being 4790 for the seven-parent-MAGIC panel.

The phenotypic variance explained (PVE) by SNPs were estimated for all traits as proposed by Martinez et al. (2018) (Eq. 4):

4 $${r}^{2}= \frac{{\sum }_{i=1}^{n}{(\widehat{{y}_{i}}-\widehat{y})}^{2}}{{\sum }_{i=1}^{n}{({y}_{i}-y)}^{2}}$$

where *y*_i_ is the phenotypic value of the *i*th observation (not corrected for any effects as included in Eq. 1) and $\widehat{{y}_{i}}$ is the predicted value of the *i*th observation when phenotypes are fitted as a linear regression of the genotype of the significant SNP(s); *n* is the total number of observations.

### Syntenic alignment plots

The QTL from the present study (dataset name: Skovbjerg_2022), together with sequence-based markers (dataset name: Vfaba_hedin_v1.Vfaba_v2.physical) were mapped to the faba bean genome and loaded into *Pulses Pretzel* (https://pulses.plantinformatics.io/). In addition, the *Medicago truncatula* genome (MedtrA17_4.0, accession GCA_000219495) and its annotation was loaded, with the flowering time genes from Yeoh et al. (2013) defined. To establish syntenic alignments between genomes, CDS sequences from *Medicago truncatula* were mapped with BLAST 2.11.0 against the faba bean genome, requiring more than 70% coverage. *Pulses Pretzel* was used to visualize all the available data for flowering time into a single plot. Singleton, long-range cross-overs in the synteny alignment were removed from the plot to increase the clarity of the projection. The plots can be recreated following the instructions given in Supplementary Note 1.

## Results

### Quality filtering and genomic distribution of SNPs

After quality control, a total of 2,682 accessions from eight different panels were genotyped for 21,345 high-quality SNP markers. Since 6 accessions appeared twice by name within a panel, we checked for genetic identity between these expected duplicates using a threshold of ≥ 94% (Eq. 1). This removed a total of four accessions, leaving a final set of 2678 accessions (Table 1). SNPs were well-distributed across chromosomes, and the average SNP density was 1.9 SNPs/Mbp (Table 2). The average distance between two adjacent SNPs was 542.8 kbp.

**Table 2:** SNP distributions and nucleotide diversities

Chr	Length (bp)	# SNPs	SNP density^a^	SNP distance^b^	π (nucleotide diversity)							
					EUCLEG	NORFAB	ProFaba	GWB	RSBP	VICCI	Seven-parent-MAGIC	Four-way-cross
Chr1S	1,574,527,093	2262	1.4 (10)	705.6 (28,576.9)	0.33	0.33	0.33	0.29	0.30	0.31	0.30	0.27
Chr1L	1,805,244,829	3852	2.1 (14)	475.7 (31,207.6)	0.32	0.32	0.32	0.29	0.29	0.30	0.29	0.27
Chr2	1,716,769,615	3633	2.1 (19)	478.4 (22,075.1)	0.32	0.32	0.32	0.29	0.28	0.29	0.28	0.25
Chr3	1,637,815,978	3292	2.0 (20)	509.6 (41,013.2)	0.32	0.32	0.32	0.29	0.28	0.29	0.29	0.27
Chr4	1,645,877,737	3008	1.8 (13)	566.5 (60,679.0)	0.32	0.32	0.32	0.28	0.29	0.29	0.28	0.26
Chr5	1,365,994,436	2611	1.9 (19)	548.8 (68,652.8)	0.32	0.33	0.33	0.28	0.29	0.30	0.29	0.26
Chr6	1,520,236,431	2687	1.8 (18)	597.3 (86,906.2)	0.32	0.32	0.32	0.28	0.28	0.30	0.29	0.26
Avg	1,609,495,160	3049	1.9	542.8 (86,906.2)	0.32	0.32	0.32	0.28	0.29	0.30	0.29	0.26

*Chr* chromosome

^a^Average number of SNPs per 1 Mbp; numbers in parentheses indicate the maximum number of SNPs found within a 1 Mbp window

^b^Average spacing between neighboring SNPs in kbp; numbers in parentheses indicate the maximum distance between SNPs

### Characterization of individual panels

Most inbred panels showed average observed heterozygosity equal to or below 0.06. An exception was the EUCLEG panel, which comprised a group of accessions with higher heterozygosity. The outcrossing VICCI panel showed higher average heterozygosity than the other panels. As expected, all inbred panels had an average observed heterozygosity (*H*_o_) which was considerably lower than the expected heterozygosity (*H*_e_) (Supplementary Fig. 1).

**Fig. 1:**
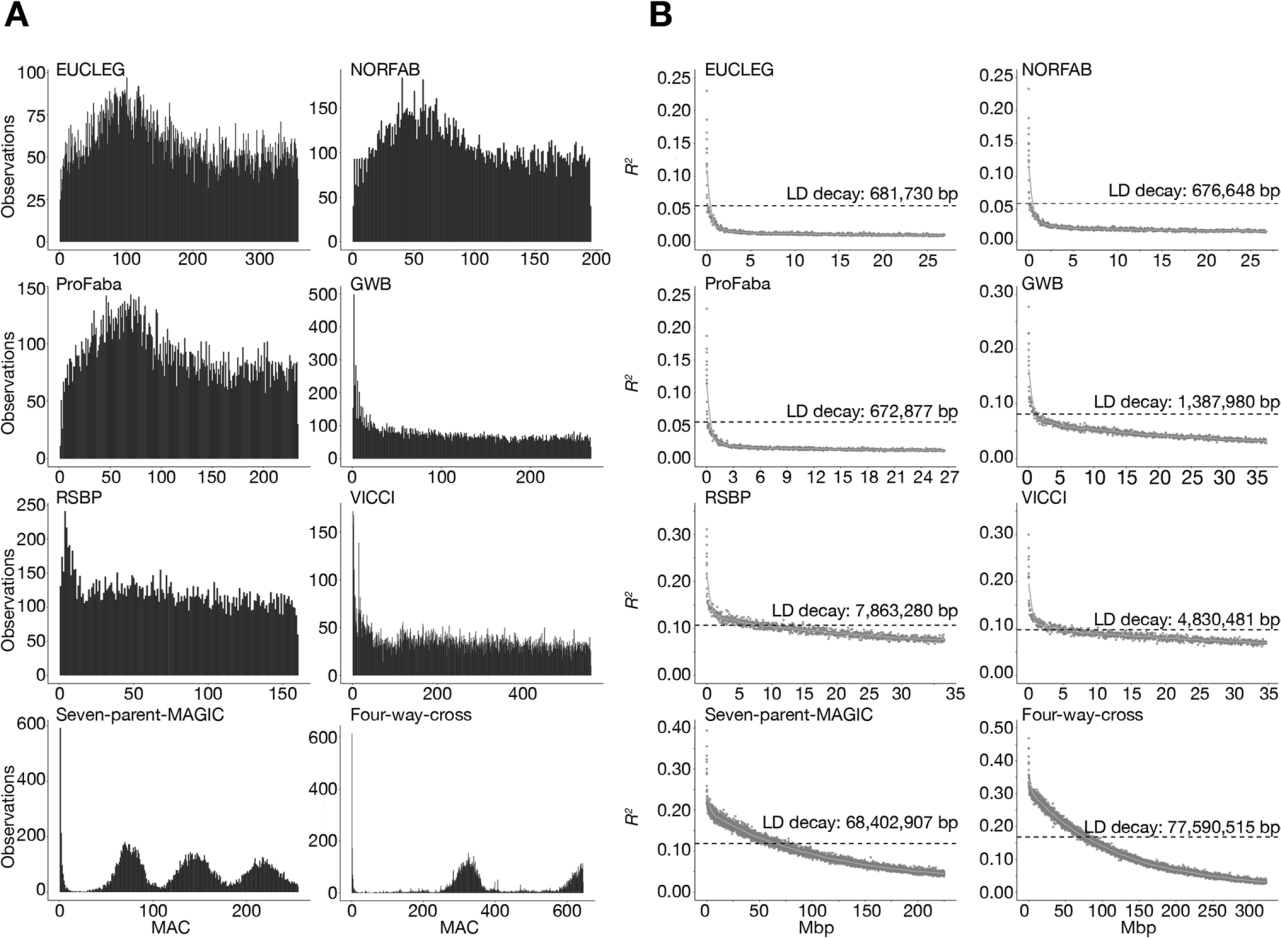
Genetic structure of individual panels. **A** Folded size frequency spectrums of the eight panels show the panel-wise distribution of minor allele counts (MAC). **B** Panel-wise LD decay plots. *Y*-axis displays the average squared correlation coefficient (*R*^2^) between markers when sorted after the average distance and binned into groups of 1000 or 5000 seven-parent-MAGIC and four-way-cross). For each bin, the *x*-axis displays the average distance in Mbp between two SNPs. The green line is the fitted loess curve with half its maximum *R*^2^ indicated by the dashed line

To compare the genetic diversity captured within each individual panel, we investigated the distribution of minor allele counts (MACs) and calculated nucleotide diversities (*π*). The nucleotide diversity was highest for the broad diversity panels, EUCLEG, ProFaba and NORFAB (0.32), whereas the remaining panels, which were all established from a limited number of founder lines, had lower π-values (0.26–0.30) (Table 2). The distribution of MACs was very similar for EUCLEG, NORFAB, and ProFaba, which showed a close-to-uniform distribution with a small overrepresentation of intermediate frequencies. For the remaining panels, we observed an excess of low-frequency variants. The distribution of MACs for the mapping panels, seven-parent-MAGIC and four-way-cross, was multimodal and reflected the numbers of founder alleles present, e.g., MACs of ~ 75 (1/7), ~ 150 (2/7), and ~ 220 (3/7) for the seven-parent-MAGIC population (Fig. 1A).

For the EUCLEG, NORFAB, and ProFaba panels, LD dropped to half of its maximum at values close to the average distance between SNPs—that is, 681.7 Kbp, 676.6 Kbp, and 672.9 Kbp, respectively. LD decayed over larger distances for the GWB (1.4 Mbp), VICCI (4.8 Mbp), and RSBP (7.8 Mbp) panels, consistent with the lower number of recombinations represented in the respective panels. The seven-parent-MAGIC and four-way-cross panels showed much larger LD blocks with average decay values of 68.4 Mbp and 77.6 Mbp, respectively (Fig. 1B**)**.

### Key agronomic traits in the seven-parent-MAGIC population

To get a better understanding of the genetic basis of key agronomic traits in faba bean, the seven-parent-MAGIC panel was phenotyped for a wide range of traits during the two years of field trials at two locations in Denmark (Supplementary Fig. 2, Table 3). In the multi-environmental ANOVA models, all traits had a statistically significant contribution from the genotypic variance to the overall phenotypic variance (*p* < 0.05). Additionally, we found that the replicate variance, the environmental variance, and the GxE interaction were significant (*p* < 0.05) for all traits, except susceptibility to downy mildew as measured in percentage (Supplementary File 4). Seed traits showed relatively little environmental influence and high heritabilities of 0.96–0.98 (Supplementary Fig. 3, Supplementary File 4). When including data from multiple environments, we found low heritabilities for disease resistance to chocolate spot as measured in percentage (0.21) and rust (0.28–0.30) (Supplementary File 4). When considering environments separately, however, heritabilities above 0.50 could be found for at least one of the environments scored for these traits (Supplementary File 4). Because of this, and the significant GxE interactions for almost all traits, we performed GWAS for each environment separately and by using the BLUEs of combined environments.

**Table 3:** Descriptive statistics of the GWAS traits

Trait	Dyn20	Sej20	Dyn21	Sej21
**Susc. to cs**				
Mean (CV%)	3.6 (72.2)	13.9 (74.1)	–	–
Range	0.2–15.0	1.0–75.0	–	–
**Susc. to rust cha**				
Mean (CV%)	–	–	2.8 (32.1)	0.2 (300)
Range	–	–	0–6	0–5
**Susc. to rust %**				
Mean (CV%)	5.1 (72.5)	4.5 (84.4)	–	–
Range	0.0–20.0	0.0–25.0	–	–
**Susc. to rust dm cha**				
Mean (CV%)	–	–	1.5 (66.7)	1.1 (81.8)
Range	–	–	0–5	0–5
**Susc. to rust dm%**				
Mean (CV%)	4.5 (68.9)/4.8 (64.6)	–	–	–
Range	0.2–20.0/0.5–25.0	–	–	–
**Herbicide damage**				
Mean (CV%)	–	–	0.5 (120)	–
Range	–	–	0–3	–
**Branching**				
Mean (CV%)	1.3 (23.1)	–	–	–
Range	0.4–3.1	–	–	–
**Plant height**				
Mean (CV%)	96.3 (13.9)	82.2 (12.7)	90.1 (13.3)	
Range	55.0–145.0	58.0–130.0	50.0–140.0	
**Sterile tillers**				
Mean (CV%)	0.4 (125.0)	–	–	–
Range	0.0–4.1	–	–	–
**Number of ovules**				
Mean (CV%)	3.3 (15.2)	–	–	–
Range	2.0–5.0	–	–	–
**Lodging**				
Mean (CV%)	4.3 (41.9)	–	1.2 (141.7)	3.8 (50.0)
Range	0–9	–	0–9	0–9
**Maturation**				
Mean (CV%)	136.4 (1.5)	–	–	–
Range	133–151	–	–	–
**Earliness of flowering**				
Mean (CV%)	71.9 (3.2)	68.6 (4.1)	70.2 (2.8)	63.8 (2.4)
Range	66–77	63–80	67–77	60–70
**End of flowering**				
Mean (CV%)	88.0 (9.4)	–	–	–
Range	77–116	–	–	–
**Duration of flowering**				
Mean (CV%)	15.2 (26.3)	–	–	–
Range	4–32	–	–	–
**TGW**				
Mean (CV%)	554.9 (26.1)	430 (26.7)	491 (21.2)	487.5 (23.1)
Range	238.2–1346.7	86.8–839.4	266.0–900.5	212.0–827.0
**Seed area**				
Mean (CV%)	91.3 (21.1)	74.2 (21.8)	86.1 (19.3)	81.7 (20.8)
Range	46.8–158.5	32.2–124.6	50.2–140.7	40.3–131.8
**Seed length**				
Mean (CV%)	12.5 (11.2)	11.5 (11.3)	12.1 (9.9)	11.9 (10.9)
Range	8.8–16.3	7.4–15.2	9.1–15.7	8.2–15.1
**Seed width**				
Mean (CV%)	9.5 (10.5)	8.6 (10.5)	9.2 (9.8)	9.1 (9.9)
Range	6.7–12.9	5.7–11.2	7.1–11.6	6.5–11.8

Two rows are dedicated to each trait-environment combination. The first row states the phenotypic mean followed by a parentheses which contains the coefficient of variation in percentage. The second row reports the phenotypic range as given by an interval of the minimum and maximum observed values. *cha.* Character, *CV* coefficient of variation, *cs* chocolate spot, *dm* downy mildew, *Dyn20* Dyngby 2020, *Dyn21* Dyngby 2021, *Sej20* Sejet 2020, *Sej21* Sejet 2021, *susc.* Susceptibility, *TGW* thousand grain weight

### Genome-wide association studies for key agronomic traits

**Fig. 2:**
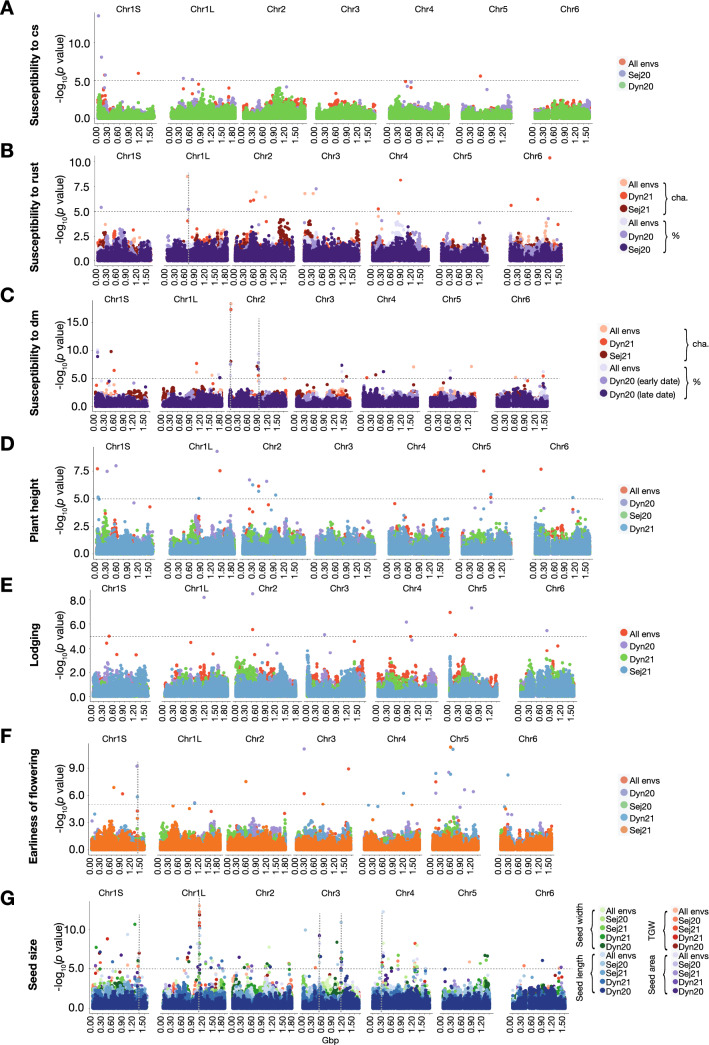
Manhattan plots of selected GWAS results in the 7-Parent-MAGIC panel. **A**–**C** Disease susceptibility to chocolate spot (**A**), rust (**B**), and downy mildew (**C**). **D** Plant height. **E** Lodging. **F** Earliness of flowering. **G** Seed size traits, i.e., thousand grain weight, seed area, seed width, and seed length. The dashed horizontal line indicates the SimpleM-corrected threshold for significance. The dashed vertical lines display broad genetic regions (peaks) made up of relatively close markers associated with multiple environments and/or measurements of the same trait

GWAS was performed using FarmCPU and the Q-Q-plots associated with the GWAS results raised no concerns regarding genomic inflation (Supplementary Fig. 4). We identified 238 (177 unique) markers associated with statistically significant signals for the following traits: earliness of flowering; plant height; lodging; sterile tillers; seed length, width, and area; TGW; herbicide damage; and susceptibility to chocolate spot, rust, and downy mildew (Supplementary File 5). Manhattan plots for multi-environmental traits—that is, susceptibility to chocolate spot, rust, and downy mildew; plant height; lodging; earliness of flowering; TGW; seed area, length, and width—are shown in Fig. 2. Manhattan plots for the remaining traits can be seen in Supplementary Fig. 5.

Of the 238 marker-trait associations, 230 originated from multi-environmental traits. Of these, 65 were stable across all environments and the associated markers explained between 0.03% (TGW) and 21.8% (seed width) of the overall trait variation (Table 4). Although only 10 of these markers point to major-effect QTLs (PVE(%) > 10%), they are, due to their stability, considered to report reliable trait-associated loci. In addition to single stable markers across environments, overlaying the Manhattan plots resulting from GWAS of multiple environments of the same trait allowed us to identify broad genetic regions (peaks) made up of clusters of markers associated with multiple environments and/or measurements of the same trait. Such peak-contributing genomic regions were also considered to be highly reliable candidates in identifying stable QTLs associated with traits (Fig. 2, Supplementary File 5).

**Table 4:** Stable genome-wide significant markers

Trait	SNP ID	Chromosome	Position	*P *value	Phenotypic variance explained (%)
Susceptibility to chocolate spot	AX-416788629	1S	1,126,488,632	1.10E−06	2.4
	AX-181482207	1S	189,966,542	1.89E−06	1.4
	AX-416797900	5	512,151,958	2.63E−06	0.5
Susceptibility to rust cha	AX-416816373	1L	609,902,557	2.84E−09	0.3
	AX-181482848	2	623,662,818	1.15E−07	1.5
	AX-416763140	2	890,099,195	3.73E−07	0.3
	AX-416742669	3	23,188,621	1.63E−07	0.1
	AX-181153730	3	266,769,997	1.62E−07	0.2
Susceptibility to downy mildew cha	AX-416819177	1L	1,030,914,144	7.63E−07	4.4
	AX-181159680	1L	1,502,215,608	2.67E−06	2.8
	AX-416747244	2	42,451,531	4.52E−19	1.0
	AX-416820991	2	1,683,844,226	1.05E−05	7.3
	AX-416728514	4	1,527,172,136	8.96E−08	2.9
	AX-181460581	5	1,255,992,674	7.70E−08	3.2
	AX-181193226	6	560,591,980	6.32E−06	1.2
Susceptibility to downy mildew %	AX-181484321	1S	64,917,771	7.51E−11	2.8
	AX-181463708	1L	1,703,280,330	1.98E−06	6.3
	AX-416817171	2	26,807,439	3.26E−06	0.9
	AX-181189191	2	880,296,875	3.79E−10	5.1
	AX-181191536	3	1,387,296,802	6.13E−07	6.5
	AX-416735217	5	602,985,248	4.01E−07	7.6
	AX-181175083	6	1,399,261,027	6.30E−07	1.0
Plant height	AX-416796020	1S	23,533,826	1.94E−08	10.8
	AX-181446054	1L	1,404,134,788	2.90E−08	6.0
	AX-181187562	2	441,935,150	7.67E−07	2.2
	AX-416787239	5	605,128,659	3.09E−08	2.1
	AX-416813291	5	801,083,781	7.66E−06	6.0
	AX-416777072	6	163,296,259	2.12E−08	0.2
Lodging	AX-416775708	1S	455,314,558	9.75E−06	2.4
	AX-181197227	2	480,659,904	2.81E−06	0.1
	AX-416757116	5	13,131,678	1.07E−07	1.2
	AX-416803185	5	162,933,615	7.75E−06	4.4
Earliness of flowering	AX-416722153	1S	922,704,193	6.56E−07	6.1
	AX-416722950	3	235,822,590	6.19E−07	0.1
	AX-416824308	3	1,567,679,832	1.24E−09	1.0
	AX-181153171	5	91,387,301	3.27E−08	1.0
TGW	AX-416754977	1S	1,016,983,171	3.51E−06	11.0
	AX-416726585	1L	1,060,752,872	9.51E−14	2.9
	AX-181462618	2	452,735,554	5.21E−06	16.7
	AX-181483910	4	1,216,173,552	1.61E−06	9.1
	AX-416747467	5	782,853,817	1.22E−06	1.0
	AX-416811554	6	1,348,503,534	8.30E−06	0.0
Seed area	AX-181481959	1S	1,318,697,535	3.95E−07	13.5
	AX-416722749	1L	1,014,586,839	7.14E−06	0.1
	AX-181487107	1L	1,439,666,414	1.43E−06	8.3
	AX-181193698	3	484,841,073	5.76E−09	4.7
	AX-181487700	4	299,822,811	8.65E−08	2.8
	AX-181483910	4	1,216,173,552	1.33E−05	14.2
	AX-181182248	4	1,281,997,173	5.24E−07	2.1
Seed length	AX-416754977	1S	1,016,983,171	4.31E−10	6.0
	AX-181194033	1L	1,001,211,223	6.24E−09	2.3
	AX-416780606	1L	1,075,870,570	6.01E−10	0.8
	AX-181178807	3	479,477,926	1.69E−06	19.2
	AX-181487700	4	299,822,811	5.29E−13	3.2
	AX-416747267	4	1,177,761,830	8.92E−07	16.5
	AX-416789448	4	1,526,450,873	9.10E−06	6.5
Seed width	AX-181481959	1S	1,318,697,535	1.66E−06	18.8
	AX-181170911	1L	1,049,955,413	4.59E−07	0.2
	AX-181487107	1L	1,439,666,414	8.50E−07	21.8
	AX-416796690	2	1,056,170,084	1.70E−08	3.8
	AX-181193698	3	484,841,073	1.81E−06	2.9
	AX-181487700	4	299,822,811	5.44E−09	2.8
	AX-416814129	4	444,381,662	4.97E−07	13.0
	AX-181483910	4	1,216,173,552	3.32E−06	0.4
	AX-181182248	4	1,281,997,173	9.84E−09	1.3

*cha.* character, *TGW* thousand grain weight

For the disease susceptibility traits, 58 marker-trait associations were identified, of which 22 were stable across environments (chocolate spot: 3/8, rust: 5/15, downy mildew: 14/35). All stable markers had a minor effect on trait variation (PVE(%) < 10%). Broader peaks were found for susceptibility to rust at Chr1L 609,902,557–635,636,923 (~ 26 Mbp) and for susceptibility to downy mildew at the following genomic locations: Chr2 26,807,439–42,451,531 (~ 16 Mbp) and 839,256,282–880,296,875 (~ 41 Mbp) (Fig. 2A–C, Supplementary File 5). For plant height, 18 marker-trait associations were significant. Six of the associations were stable across environments and individually explained up to 10.8% of all trait variance (Fig. 2D). Lodging gave rise to ten significant associations, four of which were stable across environments. All of the markers associated with lodging had relatively small effects (Fig. 2E). For earliness of flowering, 21 significant associations were identified of which 4—located on chromosomes 1S, 3, and 5—were stable across environments. Additionally, a region at chromosome 1S 1,352,951,752–1,362,763,661 (~ 10 Mbp) seemed to be associated with the trait in multiple environments (Fig. 2F). A total of 123 (83 unique) significant markers were identified for traits related to seed size—that is, seed area, seed width, seed length, and TGW (Fig. 2G). Interestingly, we identified genomic regions that were associated with multiple seed size-related traits and were stable across many environments; therefore, these can be regarded as highly reliable loci for controlling seed size. The most remarkable of these was a 26 Mbp region at chromosome 1L (1,049,955,413–1,075,870,570) that consists of 13 significant marker-trait associations and spans 101 genes. Additional stable regions associated with seed size were found at Chr1S 1,318,461–1,347,658,420 (~ 29 Mbp); Chr3 479,473,217–484,841,073 (~ 5 Mbp), Chr3 1,012,848,106–1,140,401,114 (~ 128 Mbp), and Chr4 269,654,967–299,822,811 (~ 30 Mbp) (Fig. 2G).

### A panel capturing the global faba bean diversity

**Fig. 3:**
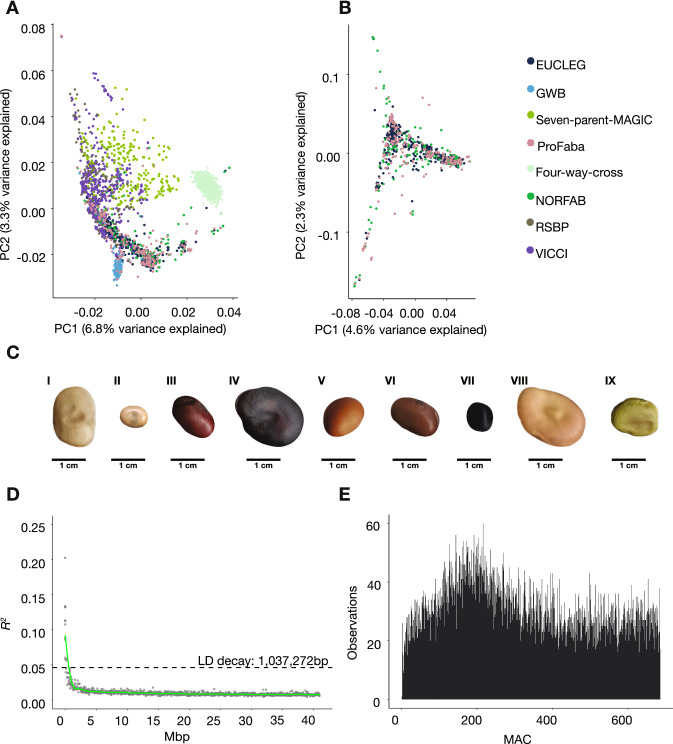
Characterization of the diversity panel. **A**, **B** The genetic structure of the data, as indicated by the first and second principal components and color-coded by panel membership **A** All panels, *n* = 2678. **B** The inbred EUCLEG, ProFaba, and NORFAB diversity panels, *n* = 787. **C** An image-based representation of the large phenotypic variation of seeds in the diversity panel. I) GPID_00080, II) EUC_VF_131, III) GPID_00162, IV) GPID_00176, V) GPID_00163, VI) GPID_00119, VII) GPID_00004), VIII) EUC_VF_272, IX) GPID_00042. **D** LD decay plot for the diversity panel. *Y*-axis displays the average squared correlation coefficient (*R*^2^) between markers when sorted after the average distance and binned into groups of 1000. For each bin, the *x*-axis displays the average distance in Mbp between two SNPs. The green line is the fitted loess curve with half its maximum *R*^2^ indicated by the dotted line. **E** Folded site-frequency spectrum of non-monomorphic SNPs in the diversity panel. The *x*-axis reports the minor allele counts (color figure online)

To investigate the genetic characteristics of the eight panels, a PCA plot was generated based on the 2678 studied accessions (Fig. 3A**)**. Given the high number of accessions, the first two PCs explained a noticeable share of the overall genetic variance (10.1%). We found that the plot showed a clear panel structure. Most obvious was the four-way-cross accessions, which formed a tight cluster clearly separated from the remaining panels. Additionally, the GWB accessions formed a tight cluster, suggesting relatively large genetic differences between winter and spring varieties.

To establish a diversity panel of inbred lines, we removed the populations that were outbred (VICCI) or generated from a limited number of founders (seven-parent-MAGIC, four-way-cross, RSBP, GWB). This left us with 787 combined accessions from the EUCLEG, NORFAB, and ProFaba panels (Fig. 3B). The accessions mixed well in the PCA, showing no underlying panel structure. The resulting diversity panel was then filtered for redundancy at a 94% genetic identity level. This removed 102 samples and resulted in a large diversity panel of 685 non-redundant lines. For all subsequent analyses of the diversity panel, except the nucleotide diversity of genetic subpopulations, a 1% MAF filter was applied to the genotype data, leaving 21,116 markers.

Passport information for the diversity panel is included in Supplementary File 1. The lines have a wide range of geographic origins representing 52 countries. In addition, they exhibit large seed variation, with seeds ranging widely in their size, color, and morphology, as exemplified in Fig. 3C. The genetic characteristics of the diversity panel were very similar to those of the individual EUCLEG, NORFAB, and ProFaba panels. The average chromosomal LD decay dropped to half of its maximum at 1.0 Mbp and the folded site frequency spectrum showed a similar pattern to the MAC distributions of EUCLEG, NORFAB, and ProFaba (Figs. 1, 3D, E).

### Population structure of the diversity panel

**Fig. 4:**
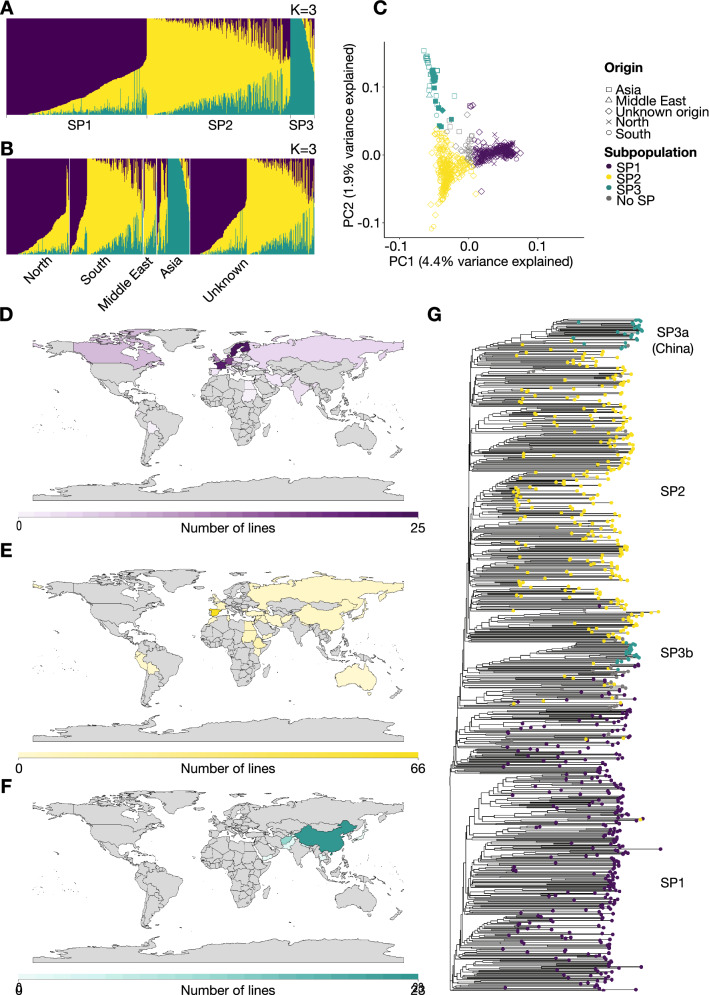
Population structure and subpopulations of the diversity panel. **A**, **B** ADMIXTURE plots at *K* = 3. Each vertical bar represents a single accession colored by its ancestry proportions. Accessions are grouped according to their subpopulation membership (**A**) or by their geographic origin (**B**). **C** Principal component analysis (PCA) based on genotypes. The ADMIXTURE subpopulations at *K* = 3 are represented by colors and geographic origin is represented by shapes. The distinction of the two phylogenetic groups of SP3 is indicated by filled (SP3a) or open points (SP3b). **D**–**F** Geographical origins of accessions belonging to SP1 (**D**), SP2 (**E**), and SP3 (**F**). Countries are colored by the number of SP accessions originating from the given country, as indicated by the scale at the bottom. For simplicity, eight lines with a geographic origin in ‘Scandinavia’ are plotted in Sweden. **G** A neighbor-joining tree of the accessions. The tips are colored by the subpopulation memberships of accessions (color figure online)

ADMIXTURE runs were performed with *K* ranging from 2 to 20. After plotting the average CV error as a function of K, we found that the local minimum was reached at *K* = 15, but that the relative reduction of the CV error when going from *K* to *K* + 1 was significantly smaller (less than 1%) after *K* = 4 (Supplementary Fig. 6A, B). With this in mind, and for interpretation reasons, we considered the best value of K to be between 2 and 4. The optimal number of K was chosen as the value where genetic subpopulations reflected geographic subpopulations to the highest degree. At *K* = 3, we found a clear correlation between the coarse geographic origin of accessions and their ancestral proportions (Fig. 4A, B). The correlation was not further resolved by setting *K* = 4 (Supplementary Fig. 6C–E). For the geographic groups, “North” covers Northern and Central Europe, Canada and Russia; “South” includes Southern Europe, South America, Africa and Australia; “Middle East” represents the Middle East; and “Asia” predominantly covers Central and East Asia. Based on membership coefficients, accessions were assigned to a subpopulation (SP). A PCA analysis of the genotypes separated accessions from different SPs by using the first two PCs. PC1 distinguished SP1 from SP2 and SP3, whereas PC2 further distinguished SP2 and SP3 (Fig. 4C). The three subpopulations were mostly reproduced in a phylogenetic analysis. However, SP3 gave rise to two different clades—one highly genetic distinct group that consisted of the Chinese germplasm (SP3a) and one containing the remaining SP3 accessions (SP3b) (Fig. 4G). The split of SP3 into SP3a and SP3b was not supported by the PCA and Admixture results (Fig. 4C, Supplementary Fig. 6). To further characterize the three inferred SPs, we looked at the exact distribution of SPs per country represented in the data (Fig. 4D–F). Supplementary File 1 includes information on geographic origin on 406 of the lines.

SP1 contains 301 accessions. Of these, 178 had a known geographic origin, and 75% of those were associated with the geographical group “North”. Among the 35 accessions associated with the geographical group “South”, 23 were French. In addition to France, the most highly represented countries/regions of origin in SP1 were Scandinavia (43), Finland (24), Germany (18), and Great Britain (12).

SP2 was made up of 304 accessions, of which 161 had a known geographic origin. The vast majority (133) was associated with the geographical group “South”. Of these accessions, 66 originate in Spain, but SP2 also includes most South American and African lines, as well as 24 Middle Eastern lines.

The smallest subgroup is SP3. It consists of 49 accessions, where the vast majority (46) have a geographic origin in Central and East Asia, predominantly China (23) and Afghanistan (12).

The remaining 31 accessions were considered admixed and were therefore not assigned to any population.

### Genetic differentiation of subpopulations

The genome-wide genetic differentiation between the three subpopulations was quantified by calculating pairwise *F*_ST_ values. SP2 was closely related to both SP1 and SP3, showing overall *F*_ST_ values of 0.06 and 0.07, respectively. SP1 and SP3 showed the highest degree of genetic differentiation with an *F*_ST_ value of 0.12 (Table 5). These results are consistent with the ability of the PC1 to completely separate accessions assigned to SP1 and SP3 (Fig. 4C). AMOVA analysis of the SPs found that 5.5% of the genetic variation was due to differences between SPs, while the remaining 94.5% of the variation was found within SPs (Table 6). To examine the amount of genetic diversity contained within each SP, we calculated their levels of expected and observed heterozygosity and genome-wide nucleotide diversity (π). We found that SP3 exhibited a lower level of observed heterozygosity (*H*_o_ = 0.03), expected heterozygosity (*H*_e_ = 0.26) and nucleotide diversity (*π* = 0.26) than the remaining SPs (Table 5). To ensure that the lower genetic diversity in SP3 was not due to its low sample size as compared to SP1 and SP2, we calculated *π* for 1000 subsets of 49 samples from SP1 and SP2 and used those in an FDR-based approach. We never observed a π-value as small as SP3 for the subsamples of SP1 and SP2 (FDR = 0) (Supplementary Fig. 7).

**Table 5:** F_ST_ analysis, nucleotide diversity and heterozygosity of subpopulations

	*F*_ST_			*π* (nucleotide diversity)	*H*_o_	*H*_e_
	SP1	SP2	SP3			
SP1	–	–	–	0.31	0.09	0.31
SP2	0.06	–	–	0.31	0.16	0.31
SP3	0.12	0.07	–	0.26	0.03	0.26

*H*_o_ observed heterozygosity, *H*_e_ expected heterozygosity

**Table 6:** AMOVA analysis

Source of variation	*df*	SSD	MSD	Percentage of variation
Among populations	2	482,764.7	241,382.4	5.5
Within populations	651	8,367,111.2	12,852.7	94.5
Total	653	8,849,875.9	13,552.6	100.0

*df* degrees of freedom, *SSD* sum of squared deviation, *MSD* mean squared deviation

### Candidate loci for population divergence

**Fig. 5:**
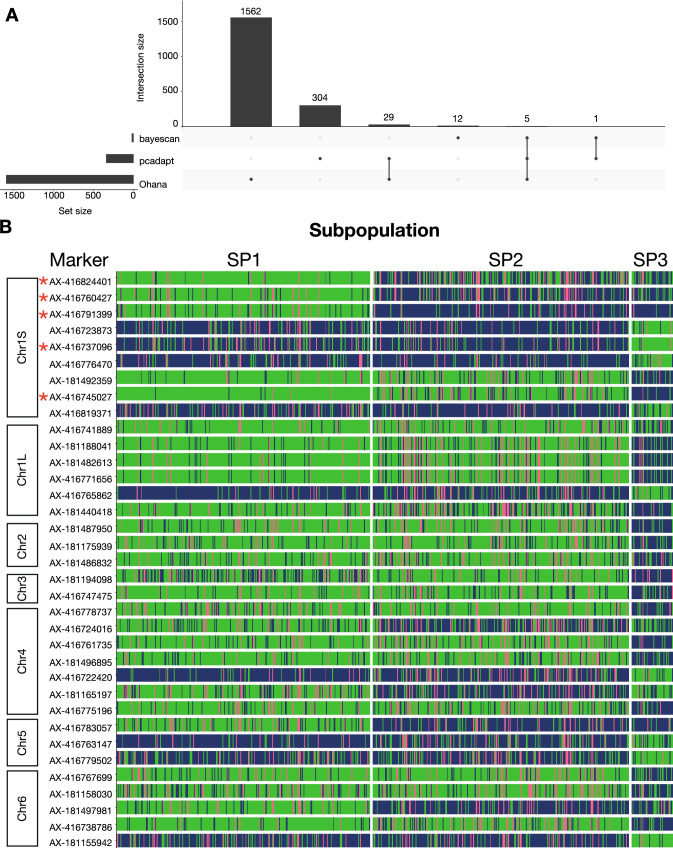
Markers under selection. **A** UpSet plot of methods used for outlier detection, showing the overlapping results of BayeScan, Ohana, and pcadapt. **B** Segregation of markers under selection. Each horizontal plot shows the segregation pattern of one of the 35 SNPs that shows evidence of selection. Markers are ordered according to genomic position. Each vertical line represents an accession and is colored by genotype for a specific marker. Genotype coloring scheme is as follows: green, reference homozygote; pink, heterozygote; blue, alternative homozygote. The five high-confidence markers identified by all outlier detection methods are marked by red asterisks (color figure online)

To explore whether the three geographically and genetically distinct SPs are under differential selection pressures and to identify genetic regions under selection, three different methods for outlier detection were applied (Fig. 5A, Supplementary File 6). BayeScan identified a total of 18 markers with *q*-values < 0.05, which show a substantial to decisive probability (0.89–1.00) of being under diversifying selection. The number of outliers detected by the other two methods were higher, with pcadapt identifying 339 significant outliers (*q*-value < 0.05) and Ohana finding 1596 SNPs with a likelihood ratio ≥ 2. Although the overlap between the methods was small, five markers were identified by all methods, giving rise to a confident set of markers pointing to direct targets of diversifying selection. In total, 35 markers were considered outliers by at least two of the three methods (Table 7). SNPs with a distance less than the average LD decay (1 Mbp) were considered a single genomic region, meaning that the analyses identified 30 genomic regions under selection, with three of the five high-confidence markers representing a single genomic region at chromosome 1S 17,355,793–18,116,022 bp.

**Table 7:** Markers under selection

SNP and genetic information					Rank		
Marker	Chr	Pos	MAF	Gene annotation^a^	pcadapt	Ohana	BayeScan
AX-416824401	Chr1S	17,355,793	0.34	Protein NRT1 PTR FAMILY	261	36	8
AX-416760427	Chr1S	17,684,368	0.45	Transaldolase/Fructose-6-phosphate aldolase	120	540	3
AX-416791399	Chr1S	18,116,022	0.45	Peptidyl-prolyl cis–trans isomerase	32	559	1
AX-416723873	Chr1S	244,272,085	0.19	Alpha-L-fucosidase	147	775	79
AX-416737096	Chr1S	245,221,411	0.24	Transcription factor	241	153	18
AX-416776470	Chr1S	1,189,800,874	0.18	Chaperone protein	134	1453	249
AX-181492359	Chr1S	1,375,226,525	0.18	Ubiquitin carboxyl-terminal hydrolase	140	1124	196
AX-416745027	Chr1S	1,376,463,514	0.18	Tobamovirus multiplication protein	38	919	4
AX-416819371	Chr1S	1,470,291,451	0.19	Receptor-like cytosolic serine threonine-protein kinase	326	992	3740
AX-416741889	Chr1L	1,210,905,827	0.16	Multiple C2 and transmembrane domain-containing protein 1	179	20,357	15
AX-181188041	Chr1L	1,727,444,242	0.20	SNF2 family *N*-terminal domain	108	709	1541
AX-181482613	Chr1L	1,727,749,638	0.18	SNF2 family *N*-terminal domain	76	1153	8595
AX-416771656	Chr1L	1,727,750,681	0.18	Glucose-induced degradation protein 8 homolog	78	1137	5621
AX-416765862	Chr1L	1,747,192,973	0.19	Sphingolipid transporter spinster homologue	166	871	6059
AX-181440418	Chr1L	1,801,916,585	0.29	Ras-related protein	176	159	5020
AX-181487950	Chr2	228,005,888	0.21	No annotation	131	456	15,934
AX-181175939	Chr2	1,160,381,636	0.21	ATPase B chain family	234	522	18,858
AX-181486832	Chr2	1,511,910,898	0.21	Involved in mitochondrial genome maintenance	302	797	2867
AX-181194098	Chr3	1,465,379,679	0.31	Pentatricopeptide repeat-containing protein	211	1039	16,108
AX-416747475	Chr3	1,549,101,456	0.18	phosphatidylglycerol acyl-chain remodeling	328	1541	9514
AX-416778737	Chr4	203,422,818	0.21	Brefeldin A-inhibited guanine nucleotide-exchange protein	254	429	7697
AX-416724016	Chr4	218,146,604	0.46	GPI mannosyltransferase	285	441	11,366
AX-416761735	Chr4	349,618,738	0.22	TIFY 10B-like	174	232	12,567
AX-181496895	Chr4	370,017,390	0.21	Protein of unknown function (DUF1296)	297	442	1259
AX-416722420	Chr4	889,829,096	0.21	Telomere repeat-binding factor	251	1423	10,805
AX-181165197	Chr4	1,086,134,085	0.49	Aldehyde dehydrogenase family	307	536	20,320
AX-416775196	Chr4	1,241,304,815	0.24	Ubiquitin carboxyl-terminal hydrolase	259	114	9265
AX-416783057	Chr5	448,641,887	0.44	Pentatricopeptide repeat-containing protein	191	590	9273
AX-416763147	Chr5	939,720,483	0.19	Nuclear transcription factor Y subunit	249	839	11,704
AX-416779502	Chr5	1,140,044,946	0.29	Pectinesterase	291	10	16,148
AX-416767699	Chr6	219,883,438	0.18	E3 SUMO-protein ligase	198	1558	19,473
AX-181158030	Chr6	265,567,017	0.27	Copper transporter	336	1283	9116
AX-181497981	Chr6	620,182,068	0.50	Zinc-RING finger domain	151	740	15,494
AX-416738786	Chr6	1,033,478,650	0.21	Cytochrome c biogenesis protein	220	1191	20,040
AX-181155942	Chr6	1,300,142,339	0.26	Ribosomal protein S1-like RNA-binding domain	288	43	8995

^a^ Annotation of closest gene if the marker is intergenic

To get a better understanding of the characteristics of the outlier SNPs, we visualized their segregation between subpopulations (Fig. 5B) and quantified the magnitudes of their *F*_ST_ signals when subpopulations were compared in a pairwise manner (Supplementary Fig. 8). We found that the 35 selection markers showed extreme differentiation between subpopulations, as compared to 35 randomly chosen markers (Supplementary Fig. 9). The vast majority of outlier SNPs, including two of the five high-confidence SNPs (AX-416737096 and AX-416745027), were related to divergence of SP3 from SP1 and SP2.

**Fig. 6:**
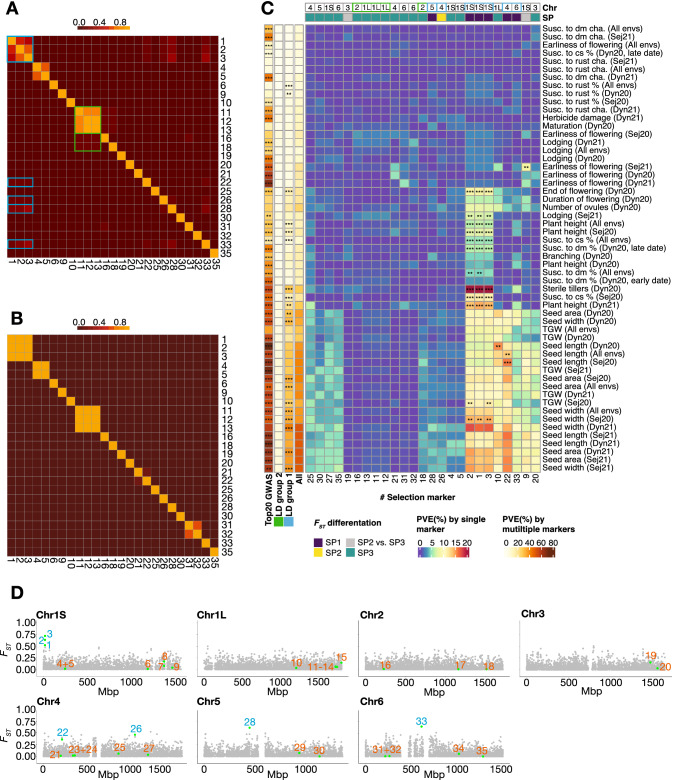
Trait variance explained by markers under selection. **A**, **B** Heatmap of LD between selection markers in the diversity panel (**A**) or the seven-parent-MAGIC panel (**B**). Markers (numerical code) are ordered according to positions in the genome. **C** Proportion of variance explained (PVE) by selection markers for all traits. PVE is calculated by all selection markers individually (the large panel), all selection markers collectively (fourth column from left), top 20 most significant GWAS markers (first column from left), all markers of LD group 2 (second column from left), and all markers of LD group 1 (third column from left). At the top of the heatmap, markers are annotated by which chromosome they are located on and which SPs they differentiate: purple, differentiation of SP1 from SP2 and SP3; yellow, differentiation of SP2 from SP1 and SP3; teal, differentiation of SP3 from SP1 and SP2; grey, differentiation of SP2 from SP3. Significance of PVE explained by different methods is calculated using an FDR-approach, where the fraction of times an obtained PVE-value was larger than what we would get from 1000 rounds of one random selected marker or different size-appropriate groups of random markers. **0.005 < FDR < 0.01; ***FDR < 0.005. **D** Genome-wide distribution of *F*_ST_ values for SP1 versus SP2. The *F*_ST_ values of each SNP throughout a chromosome are displayed as grey dots. The green dots report the 35 SNPs under selection identified in the outlier scans. The numbers next to the green dots serve as a marker code. Selection markers in panel **A**–**D** are denoted by a numerical code: 1: AX-416824401, 2: AX-416760427, 3: AX-416791399, 4: AX-416723873, 5: AX-416737096, 6: AX-416776470, 7: AX-181492359, 8: AX-416745027, 9: AX-416819371, 10: AX-416741889, 11: AX-181188041, 12: AX-181482613, 13: AX-416771656, 14: AX-416765862, 15: AX-181440418, 16: AX-181487950, 17: AX-181175939, 18: AX-181486832, 19: AX-181194098, 20: AX-416747475, 21: AX-416778737, 22: AX-416724016, 23: AX-416761735, 24: AX-181496895, 25: AX-416722420, 26: AX-181165197, 27: AX-416775196, 28: AX-416783057, 29: AX-416763147, 30: AX-416779502, 31: AX-416767699, 32: AX-181158030, 33: AX-181497981, 34: AX-416738786, 35: AX-181155942. Markers in LD group 1 are highlighted in blue (color figure online)

With the coarse geographical distinction of the SPs in mind, this clearly suggests that these markers could be associated with breeding preferences in Central and Eastern Asia (Figs. 4, 5B, Supplementary Fig. 8). Interestingly, we found that the remaining three (AX-416824401, AX-416760427, AX-416791399) of the five high-confidence markers covering the 760 kbp genetic region at chromosome 1S were associated with the differentiation of SP1 from the remaining subpopulations. The *F*_ST_ values of these markers were especially large for SP1 versus SP2 when compared to the background signal (0.52–0.71), reflecting what could be patterns of selection during breeding in Nordic environments (Figs. 5B, 6D). Although SP2 did not show large differentiation from either SP1 or SP3 (Table 5), we found one SNP on chromosome 4 (AX-181165197) that clearly separated SP2 from both remaining SPs (Figs. 5B, 6D).

### Candidate traits under selection

To get a better understanding of the selection markers and how they have been important in the global selection during breeding of faba bean, we investigated their pairwise LD in the diversity panel (Fig. 6A). We then compared the observed patterns with the pairwise LD in the seven-parent-MAGIC panel (Fig. 6B) where we were able to identify broad genetic regions associated with traits of interest (Fig. 2, Supplementary Fig. 5). As allele frequencies were different between the two panels, only 25 out of the 35 selection markers were included in the analyses. We found that all markers showing strong differentiation between northern and southern accessions—that is, between SP1 versus SP2 and SP3 or SP2 versus SP1 and SP3—showed unusual LD patterns in the diversity panel (blue boxes, Fig. 6A). This group of markers (referred to as ‘LD group 1’) consists of the three adjacent high-confidence markers at chromosome 1S, which due to their physical proximity are fully linked in the seven-parent-MAGIC panel, as well as the four remaining markers, which give rise to long-range LD, since they are located on chromosomes 4, 5, and 6 and consequently lose their LD in the seven-parent-MAGIC (Fig. 6A, B). In addition, we found another group of selection markers showing long-range LD in the diversity panel (green boxes, Fig. 6A). This group, referred to as LD group 2, was associated with the differentiation of Asian lines—that is, SP3 versus SP1 and SP2 (Fig. 5, 6A). After recombination in the seven-parent-MAGIC panel, the adjacent markers of LD group 2 showed full LD, whereas long-range LD was broken down.

To investigate possible links between the genomic regions under selection and specific traits, we resorted to the seven-parent-MAGIC panel. For each trait subjected to GWAS in the seven-parent-MAGIC panel, we calculated the proportion of phenotypic variance explained by each marker and LD groups under selection in the diversity panel (Fig. 6C). For comparison, we tested how large a fraction of the trait variation could be explained by all 25 selection markers and the top 20 most significant GWAS markers. We used top 20 markers because the 25 selection markers, when considering LD in the seven-parent-MAGIC population, behave as 20 markers (Fig. 6B).

Most of the selection markers did not explain a statistically significant proportion of variance for any of the traits. However, four of the selection markers in LD group 1 individually explained a proportion of the variance for one or more traits. Most remarkable were the three adjacent markers at chromosome 1S covering a 760 kbp genetic region, which explained a statistically significant proportion of the phenotypic variance of traits related to seed size, plant height, end of flowering, lodging, sterile tillers, and disease resistance to downy mildew and chocolate spot (Fig. 6C). These markers were among the most differentiated for SP1 and SP2 (Fig. 6D and Supplementary Fig. 8). The fourth marker was located at chromosome 4 and explained a significant proportion of variance for seed length (Fig. 6C). Expanding the single markers to the entire LD group 1, significant variance was also explained for: susceptibility to rust and several additional traits related to seed size traits. All traits that could be explained by selection markers were better explained by top GWAS markers that generally explained a large proportion of the overall trait variance. An exception to this is susceptibility to rust, where top GWAS markers did not explain a significant part of the trait variation, while LD group 1 markers did (Fig. 6C).

To better disentangle the traits significantly explained by the selection markers associated with SP1 versus SP2 differentiation (LD group 1), we looked at correlations between genetic values of the traits (as used in GWAS) (Supplementary Fig. 10, Supplementary File 7). Traits related to seed size were correlated with the following four types of traits that showed no correlations with each other: end of flowering (negative), susceptibility to rust (negative), sterile tillers (positive), and lodging (positive). Additionally, susceptibility to chocolate spot had a positive correlation with sterile tillers and lodging. Plant height and susceptibility to downy mildew show no correlation with any of the other measured traits (Supplementary Fig. 10, Supplementary File 7). From the results, it seems likely that multiple traits might have been co-selected during breeding for different market types or environments. This was further supported by the geographically distinct SPs having different proportions of allele frequencies for the 65 stable QTLs MTAs identified in GWAS (Supplementary File 5, Supplementary Fig. 11).

## Discussion

### Characterization of individual panels

Using 21,345 genome-wide high-quality SNPs, we performed genetic analyses on a large collection of faba bean germplasm. Our results revealed genetic diversity reflecting the underlying panel structure. Most strikingly, GWB, a population derived from 11 winter-type founders, was clearly genetically distinguishable from the remaining panels. As the remaining panels predominantly consisted of spring-type germplasms, this suggests that winter-type and spring-type cultivars are highly genetically distinct. A similar distinction between winter and spring-types has been described in Chinese germplasm (Zong et al. 2009; Wang et al. 2012).

The site frequency spectrum of the diversity panels revealed a relatively uniform distribution with a slight overrepresentation of markers with intermediate allele frequencies (~ 0.1–0.3). This pattern is expected because of the ascertainment bias of the Axiom SNP array, which is caused by using only 12 individuals for SNP discovery, with preference given to alleles of intermediate frequency with a high polymorphism information content (Albrechtsen et al. 2010).

The nucleotide diversity of the individual panels ranged from 0.26 to 0.32. As expected, the lowest genetic diversity was found for populations established from a limited number of founders, with the four-way-cross being the most extreme. The highest nucleotide diversities were found for the diversity panels (*π* = 0.32) and the outbreeding population (VICCI, *π* = 0.30). The nucleotide diversity in the combined diversity panel (*n* = 685) was 0.31. These values are similar to those reported using SNP data in inbred panels of maize, where values between 0.27 and 0.39 have been estimated (Hamblin et al. 2007; Lu et al. 2009; Van Inghelandt et al. 2010; Yang et al. 2011; Bouchet et al. 2013; Shu et al. 2021). The highest genetic diversity (0.39) stems from a population of 527 inbred maize lines with very broad origins (Yang et al. 2011).

### Mapping of agronomic traits

Few studies have been performed to identify QTLs of agronomically important traits in faba bean (Khazaei et al. 2021). Although a couple of recent studies have performed GWAS on unrelated and diverse faba bean germplasm (Maalouf et al. 2022; Abou-Khater et al. 2022), most of the published studies have relied on biparental populations, limiting the amount of genetic variation studied as compared to a MAGIC population. Here, we use GWAS to identify 238 significant marker-trait associations linked to 12 agronomic important traits. Of these marker-trait associations, 65 (27%) were stable across multiple environments, pointing to high-confidence candidate regions for harboring genes associated with plant height, stem lodging, earliness of flowering, seed size, and resistance to chocolate spot, downy mildew, and rust. Furthermore, all traits scored in multiple environments gave rise to stable QTLs. Among these we found major QTLs (PVE > 10%) for TGW (11.0–16.8%), seed width (13.0–21.8%), seed length (16.4–19.2%), seed area (13.5–14.3%), and plant height (10.8%). As these QTLs have major effects and are associated with 3–4 different Danish environments, they provide valuable information for future breeding programs.

Especially striking is the tall peak identified at chromosome 1L position 1,049,955,413–1,075,870,570 bp, which consists of markers significantly associated with multiple traits related to seed size (TGW, area, length, width) scored in multiple environments. Markers here explained between 0.1 and 15.8% of phenotypic variation.

Earlier studies have identified several stable QTLs associated with seed size on chromosomes 2, 4, 5, and 6 in faba bean (Khazaei et al. 2014; Ávila et al. 2017). Here, we found traits related to seed size to be highly polygenic with stable signals on all chromosomes. We checked the location of the seed weight QTLs on chromosomes 2 and 4 reported in Khazaei et al. (2014) against our QTLs for yield component traits (seed area, seed width, seed length, TGW) and found seed width (Sej21), seed area (Sej20) and TGW (Sej20) to be within the region defined by their flanking markers on chromosome 2 (Vf_Mt3g070310_001 and Vf_Mt3g065190_001). The TGW (Dyn21), seed length (Sej21) and seed width (Sej21) QTLs were within the region defined by their flanking markers on chromosome 4 (CNGC4 and Vf_Mt7g038120_001) (Supplementary Fig. 12).

Plant height is another important trait related to faba bean yield. Previous studies have performed QTL mapping of plant height but have not identified any stable QTLs across environments (Ávila 2017). In this study, we detected six QTLs that were stable across three Danish environments for plant height. The stable markers individually explained between 0.2 and 10.8% of phenotypic variation.

None of our stable flowering-related QTLs were estimated to explain a large proportion (> 10%) of the trait variation. On the contrary, our findings suggest a relatively polygenic nature of flowering, with multiple QTLs specific to environments. A major stable flowering time QTL was previously found on chromosome 5 (Cruz-Izquierdo et al. 2012; Catt et al. 2017). Interestingly, the region did not only have a large effect on the trait but is also highly conserved in multiple legumes, including *Lotus japonicus* (Gondo et al. 2007), *Medicago truncatula* (Pierre et al. 2008), chickpea (Cobos et al. 2009), narrow-leafed lupin (Nelson et al. 2006), and alfalfa (Robins et al. 2007). The region on chromosome 5 from approximately 489 Mb to 602 Mb (comprising 244 genes) contains four of the peak markers identified for flowering time in this study, the QTL for flowering time identified from a bi-parental cross by Catt et al. 2017, as well as the peak markers identified in Cruz-Izquierdo et al. (2012) and Aguilar-Benitez et al. (2021). The region is syntenic to the region of *Medicago truncatula* chromosome 7 that harbors five flowering time genes and the *spring1* locus (Yeoh et al. 2013; Supplementary Fig. 13). Inspecting protein alignments between *Medicago truncatula* and faba bean, we found three (MtFTa1, MtFTa2, MtFTc) of the five flowering time genes in the identified region of *Medicago truncatula* chromosome 7 to have putative orthologs in the corresponding region on faba bean chromosome 5 (Supplementary Fig. 13).

Stable QTLs for number of ovules and branching (number of branches with flower) has previously been reported on chromosomes 3 and 6, respectively (Ávila et al. 2017), but here we report no QTLs related to these traits. This could indicate a high genetic complexity of these traits.

One of the main threats for the global production of faba bean is foliar diseases such as rust (caused by *Uromyces viciae-fabae*), chocolate spot (caused by *Botrytis fabae*), and downy mildew (caused by *Peronospora viciae*). Due to environmental and economic reasons, breeding for disease resistance is preferred over treating crops with fungicides (Stoddard et al. 2010). Still, the genetic basis of faba bean disease resistance is to a large extent unknown.

Here, we identified several genomic regions associated with resistance toward all three fungal diseases. We especially obtained many stable marker-trait associations (14) for downy mildew, where we found very strong peaks on chromosome 2, at positions 26,807,439–42,451,531 bp and 839,256,282–880,296,875. This is of great interest, as no QTLs for this trait have, to our knowledge, yet been published for faba bean. Similar to recent studies, we found that chromosome 1 harbors QTLs associated with resistance to chocolate spot (Gela et al. 2022). For rust resistance, we found five stable markers located at chromosomes 1L, 2, and 3. Two genes associated with rust resistance in faba bean, *Uvf2* and *Uvf3*, have successfully been identified and mapped to chromosomes 3 and 5, respectively, using KASP markers (Ijaz et al. 2021). By mapping the KASP markers to our reference genome, we did not observe any overlap between the genetic regions associated with *Uvf2* and *Uvf3* and our peaks for rust resistance. This is most likely due to differences in experimental designs and genetic material.

Although we detected many high-confidence QTLs associated with key agronomic traits, the low resolution in the seven-parent-MAGIC population complicates the search for underlying candidate genes. As compared to the diversity panels, where almost no LD were detected between neighboring SNPs, larger LD blocks were observed for the seven-parent-MAGIC population. For this reason, the GWAS is expected to cover close to all genome-wide QTLs. However, this is accompanied by a poor mapping resolution when it comes to identifying genes associated with traits of interest. As the average genome-wide distance between annotated genes is 307,734 bp and the LD-decay in the population is ~ 68 Mbp, each marker-trait association is expected to report a region representing hundreds of genes. With this in mind, the presented GWAS results are useful in associating traits with mapped but relatively broad underlying genetic regions. For this reason, we suggest that future studies take advantage of the diversity panel for fine-mapping of the QTLs.

### Faba bean diversity and genetic differentiation

With a long history of cultivation and widespread adaptation, faba bean provides excellent material for studying global genetic diversity. In order to understand the genetic differentiation related to different geographic regions, we established a diversity panel using genetically non-redundant accessions from the described EUCLEG, NORFAB and ProFaba panels. In the process we removed 102 lines that we found to be genetically redundant (GI ≥ 94%). Although some accessions were present in duplicates because of their inclusion in more than one of the initial project-based diversity panels, many were also found to be genetically redundant within these panels. In general, earlier studies have reported that germplasm collections both within and between genebanks suffer from the presence of genetically redundant lines, which do not contribute to genetic diversity and complicates the genetic analyses (Song et al. 2015; Milner et al. 2019).

We divided the diversity panels into three subpopulations with different coarse geographic origins: SP1, consisting of germplasm originating mostly from Northern and Central Europe but also including all Canadian lines; SP2, which mostly consists of Spanish germplasm but also includes African, South American, and Middle Eastern varieties; and SP3, which has a narrower geographic origin, mostly consisting of Central and East countries of Asia, predominantly China and Afghanistan.

Consistent with previous studies, our analyses revealed that the genetic diversity of faba beans was highly associated with geographical origin (Kaur et al. 2014b; Wang et al. 2012; Zong et al. 2010; El-Esawi 2017). Outcomes of our PCA and* F*_ST_ studies identified the northern accessions (SP1) and Central and East Asian accessions (SP3) as genetically distinct subpopulations with southern accessions (SP2) located in between. This is also demonstrated by very few accessions showing a high degree of admixture between SP1 and SP3 and close to no geographical overlap between SP1 and SP3. Geographically, our findings fit well with the proposed routes of migration for faba bean cultivation, suggesting that different routes radiated from the Middle East (SP2). One progressed eastwards to Asia (SP3), whereas two different routes are proposed for the European cultivation—One toward the Iberian Peninsula (SP2) via the Mediterranean coast of Africa, and a second toward Northern Europe (SP1) via the Mediterranean regions of Southern Europe (SP2) (Cubero 1974).

Consistent with our findings, previous studies have reported that Asian, or specifically Chinese, germplasm is highly distinct from other germplasm (Kaur et al. 2014b; Wang et al. 2012; Zeid et al. 2003). Our findings agree with those of Zeid et al. (2003), who reported a close genetic relationship between Northern African lines and South European lines, which support the observed grouping of African and Southern European lines in SP2. Furthermore, Zong et al. (2010) reported genetic support of a subdivision of European lines into those originating from Spain versus those from Northern Europe. Other studies, however, have found that germplasm from both Southern and Northern Europe cluster together and are genetically distinct to the group formed by Asian and African germplasm (Göl et al. 2017).

The level of genetic diversity was lowest for SP3, which includes most of the Central and East Asian accessions. This is in contrast to the findings published by Zong et al. (2009), where Asian lines (excluding Chinese) showed higher genetic diversity than either the African or European lines. As our findings did not seem to be a direct consequence of the low sample size of SP3 (*n* = 49), we speculate that it might be a consequence of SP3 mostly originating from two countries (China and Afghanistan), thereby representing what might be expected to be a low effective population size compared to the remaining subpopulations.

AMOVA results revealed a higher genetic diversity within than between the three subpopulations. This is in agreement with what has earlier been found for faba bean (Göl et al. 2017; Wang et al. 2012; Oliveira et al. 2016). In our findings, the low degree of genetic variability observed between subpopulations is most likely both a result of overlapping geographical regions of SP2 and the remaining SPs, as well as an indication of global exchange of germplasm. The high degree of within-population variability is most likely due to the reproductive nature of faba bean, which is partially outcrossing (Göl et al. 2017; Brünjes and Link 2021).

### Signatures of selection

Of the total markers, 35 (0.2%) were identified to be under selection by at least two of the three outlier detection methods. In general, there was low agreement between the results of the different methods, most likely due to the different assumptions and estimation methods of the models. This helped us limit the selection signatures to a few highly confident markers that show strong differentiation between the different subpopulations. Most (26) of these markers were associated with differentiation of SP3 from SP1 and SP2, whereas only 6 markers (from four genetic regions) were associated with SP1 differentiation from SP2 and SP3. This further supports the differentiation of northern (SP1) and asian germplasm (SP3), with the southern germplasm (SP2) being located somewhere in between. Especially interesting were five selection markers that were identified by all three methods. These markers, representing three regions at chromosome 1S (approximately at 17.4–18.1 Mbp, 245.2 Mbp, and 1376.5 Mbp), show very strong selection signatures and have very likely played an important role in the geographical differentiation of faba bean.

To couple the selection signatures with their associated traits, we took advantage of the seven-parent-MAGIC panel, where we tested the amount of trait variance that markers under selections could explain compared to random markers. Interestingly, we mainly found selection markers associated with the differentiation of northern (SP1) versus southern (SP2) germplasm to explain a significant proportion of trait variances. With a key influence of the strongly differentiated region at chromosome 1S position 17.4–18.1 Mbp, the selection signatures of northern and southern accessions explained variance related to disease resistance, end of flowering, seed size, plant height, and lodging. This is in line with studies of selection in other domesticated crops such as chickpea (Varshney et al. 2019), soybean (Saleem et al. 2021), and maize (Bouchet et al. 2013), which found that genes underlying selection signatures are often associated with flowering or disease resistance.

Our results indicate that one or more of these traits could have played a role in selection for different market types or climatic conditions. Because of the large extent of LD in the seven-parent-MAGIC panel, however, we are not able to pinpoint specific causal trait(s) at this stage. With comprehensive phenotyping, the better mapping resolution of the diversity panel could help to clarify this question in future studies.

## Conclusions

This study provides valuable insights into the genetic diversity, geographical differentiation and the underlying genomic regions of key agronomic traits in faba bean. Genome-wide association studies in a MAGIC population provided high-confidence candidate genomic regions associated with seed size, flowering time, plant height, lodging and disease resistance to downy mildew, rust and chocolate spot. Our identified QTLs confirmed both previous studies and provided novel QTLs for key agronomic traits in faba bean. However, the extent of LD in the MAGIC population complicated candidate gene discovery.

Genetic analysis of a large sample of global faba bean germplasm allowed establishment of a non-redundant faba bean diversity panel representing 52 countries. Accessions in the diversity panel could be divided into three subpopulations, which showed clear genetic divergence related to their geographical origin. The largest genetic differentiation was observed between SP1, which mostly consisted of Northern European accessions, and SP3 comprising lines from Central and East Asia, predominantly China. The latter also showed lower genetic diversity than the remaining subpopulations. In addition to its role in describing global diversity in faba bean, the diversity panel constitutes a valuable resource for future breeding and high-resolution gene mapping, including candidate gene discovery for the wide genomic regions covered by the QTLs identified in the MAGIC population.

## Supplementary Information

Below is the link to the electronic supplementary material.

Supplementary Figure 1. Heterozygosity of panels. Histograms showing the heterozygosity of genotypes within each panel. The average observed (Ho) and expected (He) levels of heterozygosity are indicated by green and red lines, respectively. (PDF 1132 KB)

Supplementary Figure 2. Histograms for GWAS traits. The distributions of raw phenotypes are plotted for all traits and environments. Abbreviations: cs, chocolate spot; dm, downy mildew; Dyn20, Dyngby 2020; Dyn21, Dyngby 2021; dur, duration; ear, earliness; Sej20, Sejet 2020; Sej21, Sejet 2021; susc., susceptibility; TGW, Thousand grain weight. (PDF 2160 KB)

Supplementary Figure 3. Trait variance. Proportion of phenotypic variance of traits explained by residual variance (VarRes), replication variance (VarRep), genotype x environment variance (VarGxE), environmental variance (VarE), and genetic variance (VarG). A) All traits scored in multi-environmental field trials. B) Traits scored in one environment only. Abbreviations: cha, character; susc., susceptibility; TGW, Thousand grain weight. (PDF 489 KB)

Supplementary Figure 4. QQ-plots for all GWAS results. The plots show the observed distribution of p-values of markers tested for association in GWAS plotted against the expected distribution of p-values if no associated loci are found. Abbreviations: cha, character; Dyn20, Dyngby 2020; Dyn21, Dyngby 2021; envs., environments; Sej20, Sejet 2020; Sej21, Sejet 2021; TGW, Thousand grain weight. (PDF 3159 KB)

Supplementary Figure 5. Additional Manhattan plots. Manhattan plots for GWAS of herbicide damage, branching, number of ovules, number of sterile tillers per plant, maturation date, end of flowering, and duration of flowering. The green line indicates the SimpleM-corrected threshold for significance. Abbreviations: Dyn20, Dyngby 2020; Dyn21, Dyngby 2021. (PDF 902 KB)

Supplementary Figure 6. ADMIXTURE results. A) Cross-validation error of ADMIXTURE with K = 2 to K = 20. The bars display standard errors associated with repeating the CV 10 times for each value of K. B) ADMIXTURE proportions at K = 15 where the CV error is minimized. C–D) ADMIXTURE plots at K = 4. Each vertical bar represents a single accession colored by its ancestry proportions. Accessions are grouped according to their subpopulation membership (C) or by their geographic origin (D). E) Principal component analysis (PCA) based on genotypes. The ADMIXTURE subpopulations at K = 4 are represented by colors and geographic origin is represented by shapes. (PDF 1131 KB)

Supplementary Figure 7. Distribution of average genome-wise nucleotide-diversity (π) values of 1000 subsets (n = 49) of SP1 (A) and SP2 (B). The horizontal green line displays the π value for SP3 (n = 49). (PDF 484 KB)

Supplementary Figure 8. Genome-wide distribution of FST values for pairs of subpopulations. The FST values of each SNP throughout a chromosome are displayed as grey dots. The green dots report the 35 SNPs under selection identified in the outlier scans. The numbers next to the green dots serve as a marker code: 1: AX-416824401, 2: AX-416760427, 3: AX-416791399, 4: AX-416723873, 5: AX-416737096, 6: AX-416776470, 7: AX-181492359, 8: AX-416745027, 9: AX-416819371, 10: AX-416741889, 11: AX-181188041, 12: AX-181482613, 13: AX-416771656, 14: AX-416765862, 15: AX-181440418, 16: AX-181487950, 17: AX-181175939, 18: AX-181486832, 19: AX-181194098, 20: AX-416747475, 21: AX-416778737, 22: AX-416724016, 23: AX-416761735, 24: AX-181496895, 25: AX-416722420, 26: AX-181165197, 27: AX-416775196, 28: AX-416783057, 29: AX-416763147, 30: AX-416779502, 31: AX-416767699, 32: AX-181158030, 33: AX-181497981, 34: AX-416738786, 35: AX-181155942. Markers in LD group 1 are highlighted in blue. (PDF 747 KB)

Supplementary Figure 9. Segregation of 35 random markers. Each row shows the segregation pattern of one of 35 random markers. Each vertical line represents an accession and is colored by genotype for a specific marker. Genotype coloring scheme is as follows: green, reference homozygote; pink, heterozygote; blue, alternative homozygote. (PDF 621 KB)

Supplementary Figure 10. Genetic correlations between traits significantly explained by markers associated with North (SP1) versus South (SP2) differentiation. Green asterisk indicates statistical significance of correlation coefficients using a Bonferroni-corrected threshold of p < 0.05. Abbreviations: cha, character; cs, chocolate spot; dm, downy mildew; Dyn20, Dyngby 2020; Dyn21, Dyngby 2021; envs., environments; Sej20, Sejet 2020; Sej21, Sejet 2021; susc., susceptibility; TGW, Thousand grain weight. (PDF 350 KB)

Supplementary Figure 11. The subpopulation specific allele frequencies of the 65 stable QTLs identified in GWAS. Alleles associated with larger trait values in the GWAS population were defined as ‘positive’ (blue), whereas those associated with lower trait values were defined as ‘negative’ (pink). Asterisks indicate statistical significance of differential allele frequencies between subpopulations when corrected for multiple testing (Bonferroni correction) at p < 0.05 (*), p < 0.01 (**) and p < 0.005 significance levels. Abbreviations: cha, character; Dyn20, Dyngby 2020; Dyn21, Dyngby 2021; envs., environments; Sej20, Sejet 2020; Sej21, Sejet 2021; TGW, Thousand grain weight. (PDF 1028 KB)

Supplementary Figure 12. Synteny for QTLs related to seed size. A) Mapped QTLs for seed area, seed width, seed length and TGW on chromosome 2 (whole chromosome, left axis) and zoomed region (right side). Markers defining the QTL interval on chromosome 2 reported for seed weight in Khazaei et al. 2014 are shown in blue. B) Mapped QTLs for seed area, seed width, seed length and TGW on chromosome 4 (whole chromosome, left axis) and zoomed region (right side). Markers defining the QTL interval on chromosome 4 reported for seed weight in Khazaei et al. 2014 are shown in blue. (PDF 74 KB)

Supplementary Figure 13. Synteny for QTLs related to flowering. A) A region on Medicago truncatula chromosome 7 containing five flowering time genes, defined in Yeoh et al. 2013 (zoomed in view, axis 1). The complete Medicago truncatula chromosome 7 projected across to faba bean chromosome 5 (axes 2 and 3). Green lines indicate gene mappings by sequence from Medicago truncatula to faba bean. The earliness of flowering QTLs from the present study are indicated by colored diamonds (all environments shown in red, Dyn20 in brown, Dyn21 in purple, Sej21 in light blue). The QTL from Catt et al. 2017 identified in the Icarus x Ascot genetic map (axis 4) is projected against faba bean chromosome 5 via mapped markers (green lines connecting the genetic map to physical sequence). B) Zoomed in view of the syntenic context around the spring1 locus in Medicago truncatula chromosome 7 (left axis) and corresponding region of faba bean chromosome 7 (right axis). The plot features 5 flowering time genes in Medicago truncatula (MtFTa1, MtFTa2, MtFTc, MtFD,MtPKS) located on chromosome 7. (PDF 73 KB)

Supplementary File 1: Passport information on individual accessions(XLSX 631 KB)

Supplementary File 2: Trait descriptions (XLSX 15 KB)

Supplementary File 3: SNP markers and their positions (XLSX 809 KB)

Supplementary File 4: ANOVA results of the seven-parent-MAGIC population (XLSX 27 KB)

Supplementary File 5: Significant marker-trait associations identified by GWAS (XLSX 93 KB)

Supplementary File 6: Individual results of all three methods of outlier detection (XLSX 3005 KB)

Supplementary File 7: Correlations between genetic values of all traits (XLSX 29 KB)

Supplementary File 8: Seven-parent-MAGIC phenotypes used for GWAS (TXT 133 KB)

Supplementary File 9: Raw Phenotype Scores from Field Trials (PDF 7208 KB)

Supplementary Note 1. Pretzel Instructions (CSV 1567 KB)

Supplementary file24 (DOCX 13 KB)

## Data Availability

Data supporting the findings are available within the paper and its Supplementary Information Files. Genotype data are available at: https://figshare.com/s/a30c37481c6c8f6626e8.

## Abbreviations

cha: Character
cs: Chocolate spot
dm: Downy mildew
Dyn20: Dyngby 2020
Dyn21: Dyngby 2021
Sej20: Sejet 2020
Sej21: Sejet 2021
TGW: Thousand grain weight

## Code availability

Scripts used for data analyses and plotting are provided on github: https://github.com/cks2903/DiversityStudiesFabaBean2021.
