# Supplementary figures and images for: Genetic analysis of global faba bean diversity, agronomic traits and selection signatures
Source: Theor Appl Genet. 2023 Apr 19;136(5):114. doi: 10.1007/s00122-023-04360-8 (PMC10115707; doi:10.1007/s00122-023-04360-8)

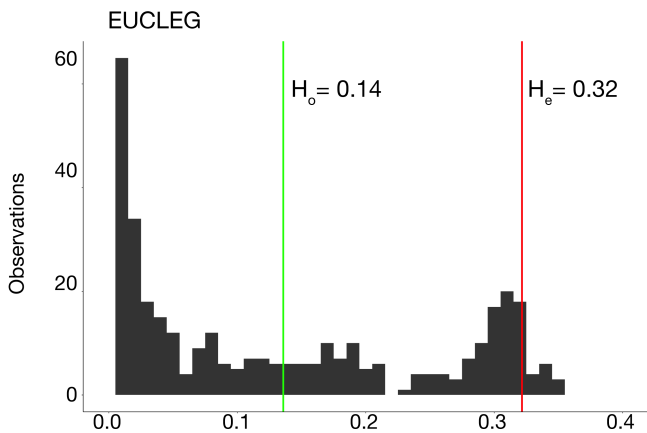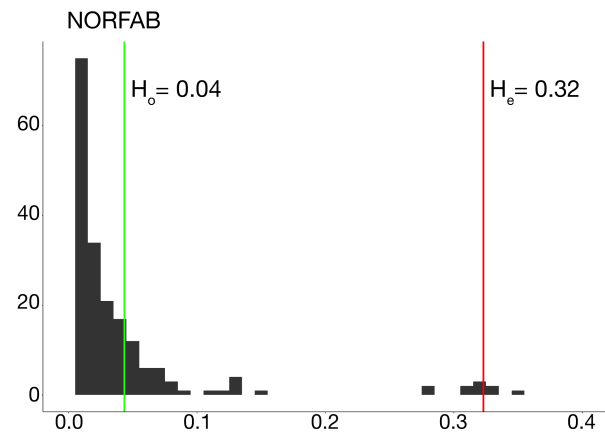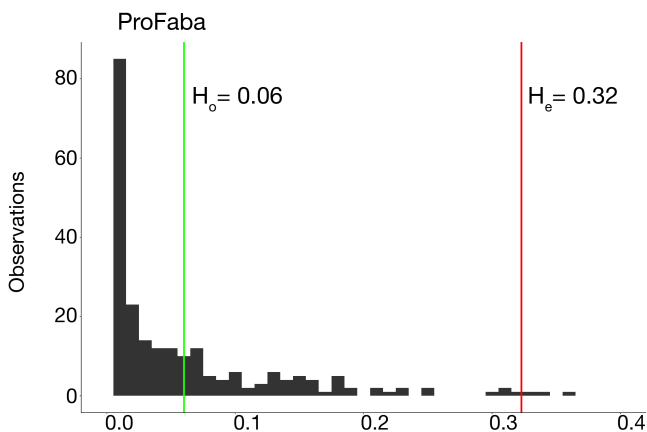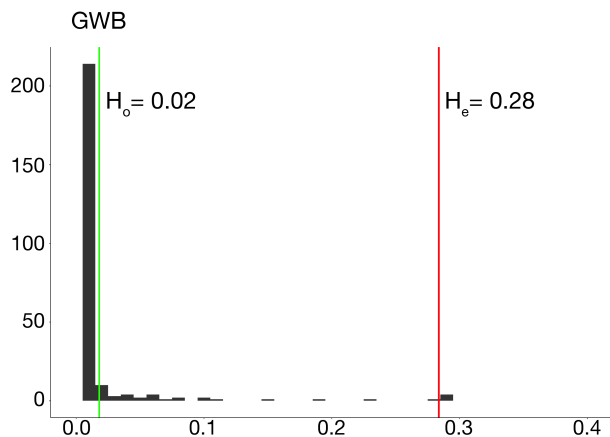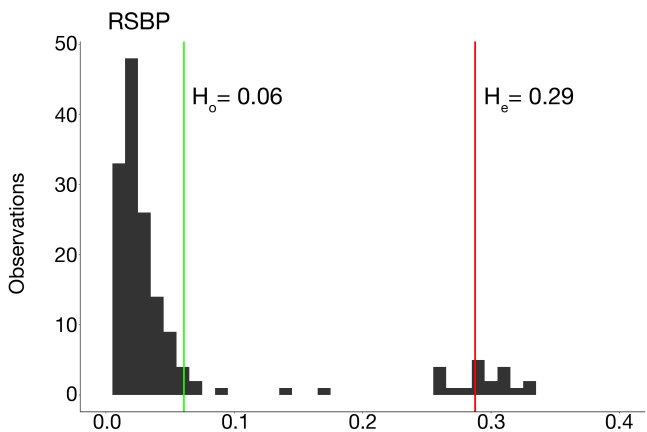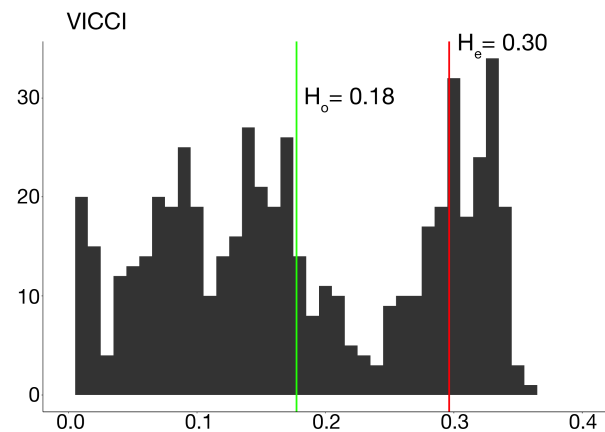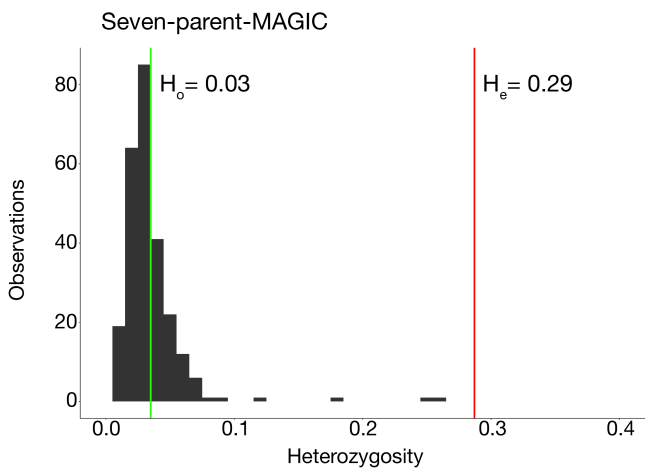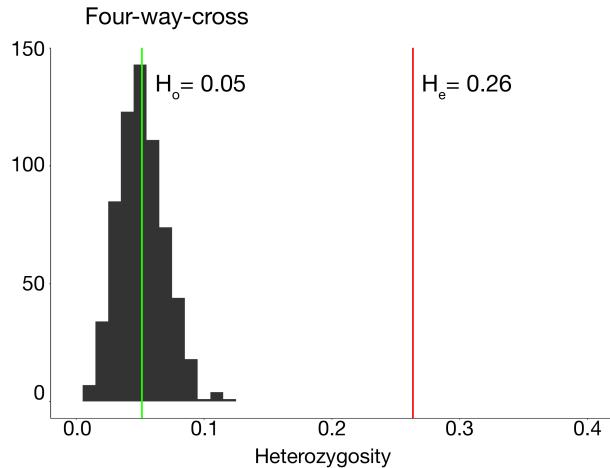

Supplement: Supplementary file 1 — Supplementary Figure 1. Heterozygosity of panels. Histograms showing the heterozygosity of genotypes within each panel. The average observed (Ho) and expected (He) levels of heterozygosity are indicated by green and red lines, respectively. (PDF 1132 KB) [file 122_2023_4360_MOESM1_ESM.pdf]

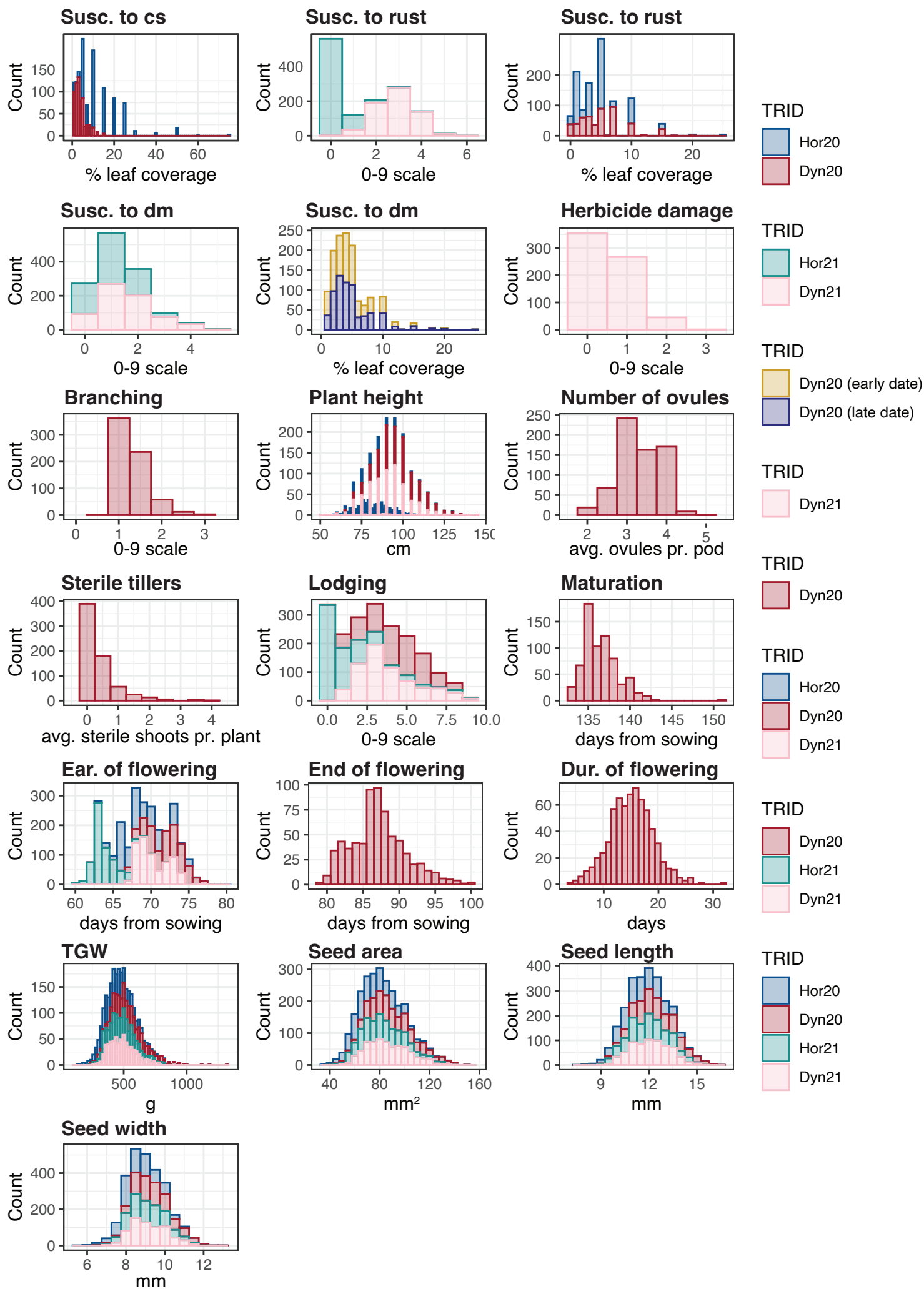

Supplement: Supplementary file 2 — Supplementary Figure 2. Histograms for GWAS traits. The distributions of raw phenotypes are plotted for all traits and environments. Abbreviations: cs, chocolate spot; dm, downy mildew; Dyn20, Dyngby 2020; Dyn21, Dyngby 2021; dur, duration; ear, earliness; Sej20, Sejet 2020; Sej21, Sejet 2021; susc., susceptibility; TGW, Thousand grain weight. (PDF 2160 KB) [file 122_2023_4360_MOESM2_ESM.pdf]

**A**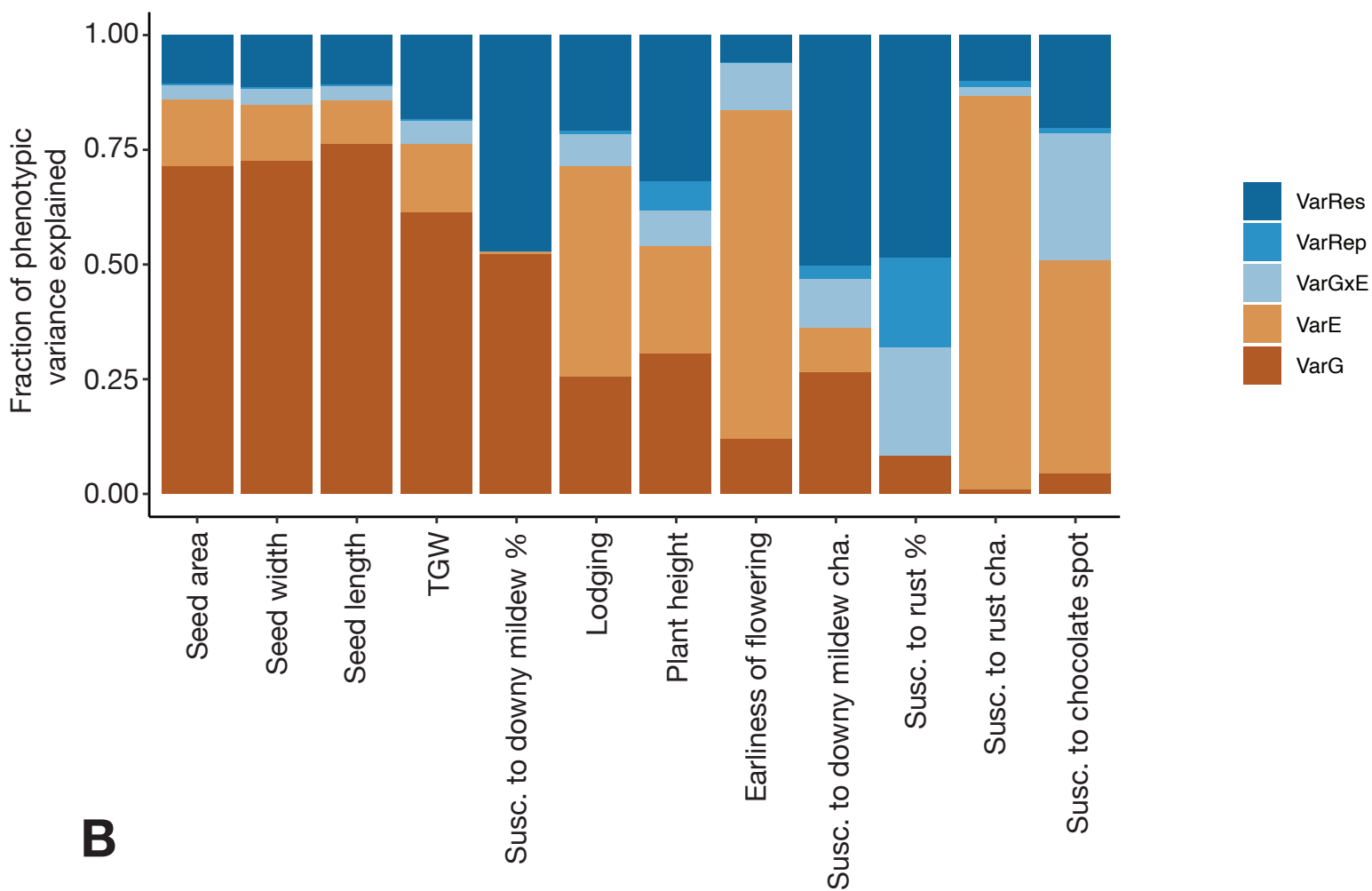**B**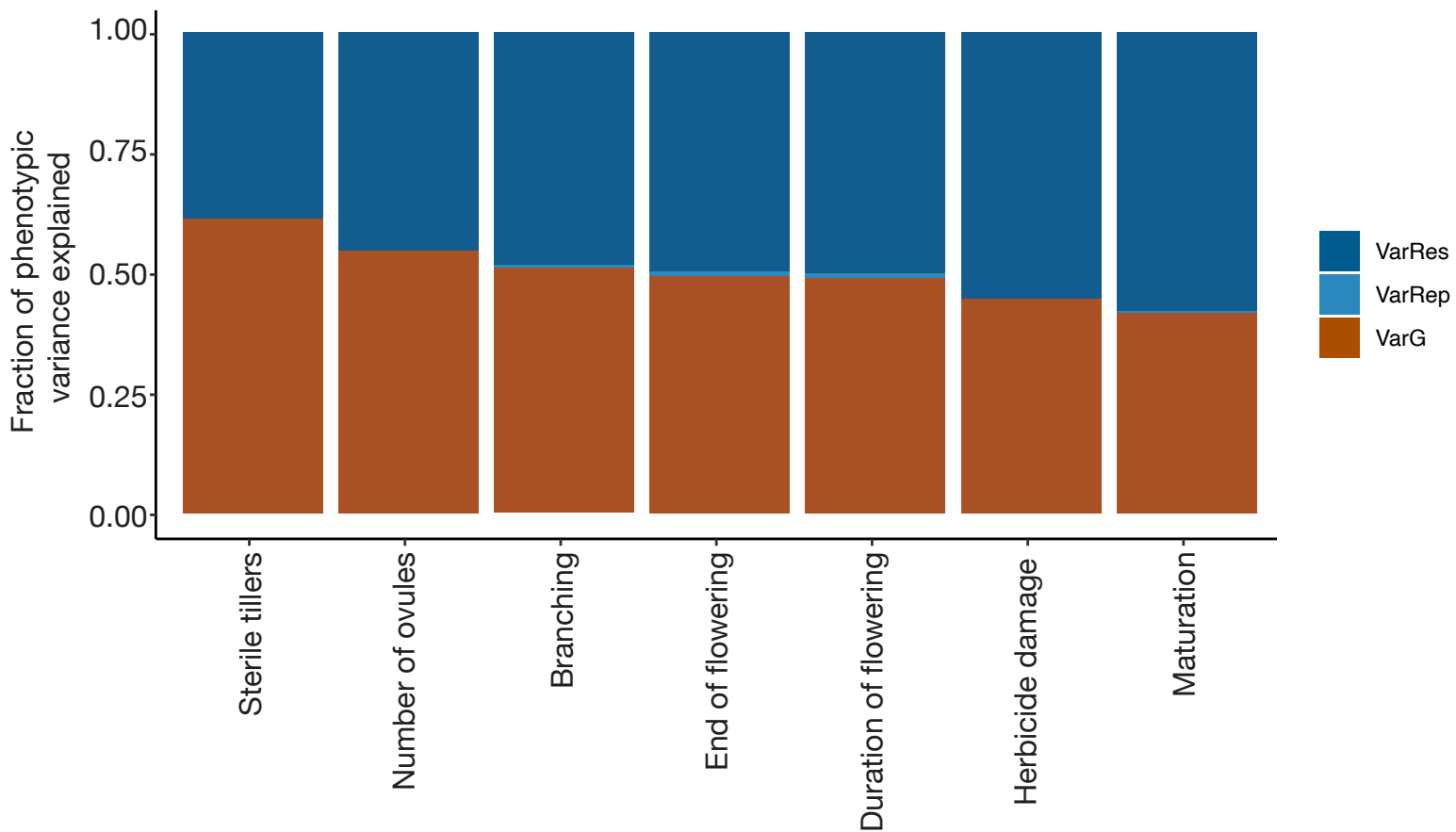

Supplement: Supplementary file 3 — Supplementary Figure 3. Trait variance. Proportion of phenotypic variance of traits explained by residual variance (VarRes), replication variance (VarRep), genotype x environment variance (VarGxE), environmental variance (VarE), and genetic variance (VarG). A) All traits scored in multi-environmental field trials. B) Traits scored in one environment only. Abbreviations: cha, character; susc., susceptibility; TGW, Thousand grain weight. (PDF 489 KB) [file 122_2023_4360_MOESM3_ESM.pdf]

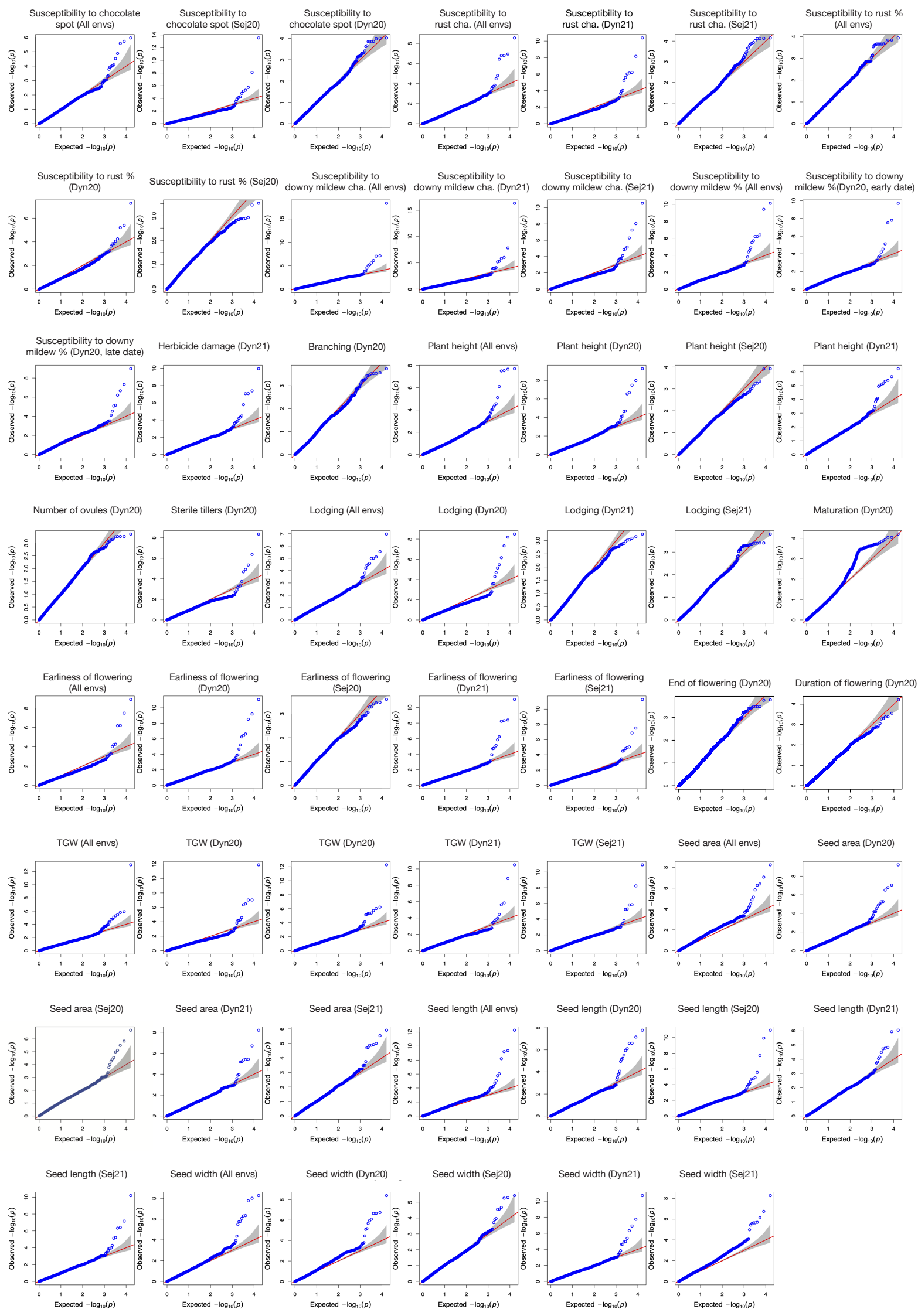

Supplement: Supplementary file 4 — Supplementary Figure 4. QQ-plots for all GWAS results. The plots show the observed distribution of p-values of markers tested for association in GWAS plotted against the expected distribution of p-values if no associated loci are found. Abbreviations: cha, character; Dyn20, Dyngby 2020; Dyn21, Dyngby 2021; envs., environments; Sej20, Sejet 2020; Sej21, Sejet 2021; TGW, Thousand grain weight. (PDF 3159 KB) [file 122_2023_4360_MOESM4_ESM.pdf]

**A**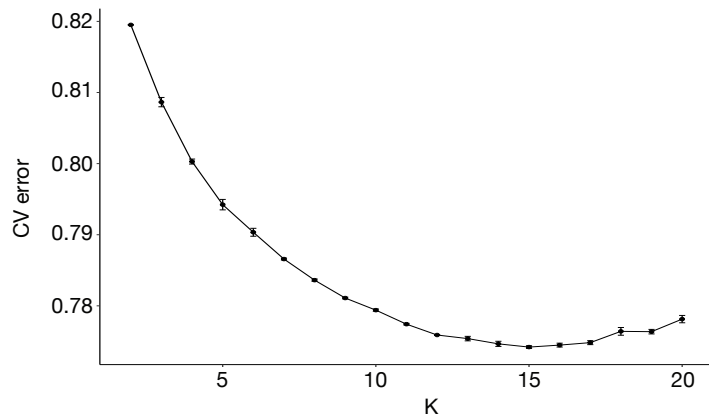**B**

K = 15

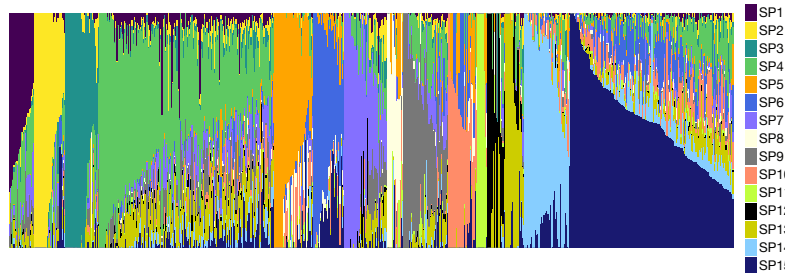**C**

K = 4

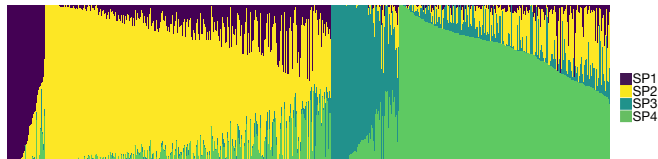**D**

K = 4

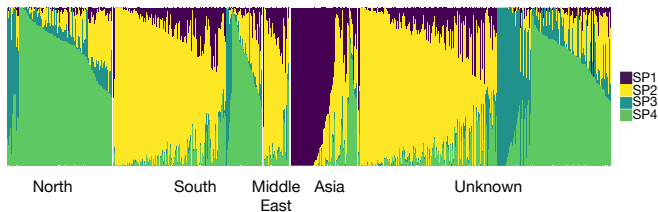**E**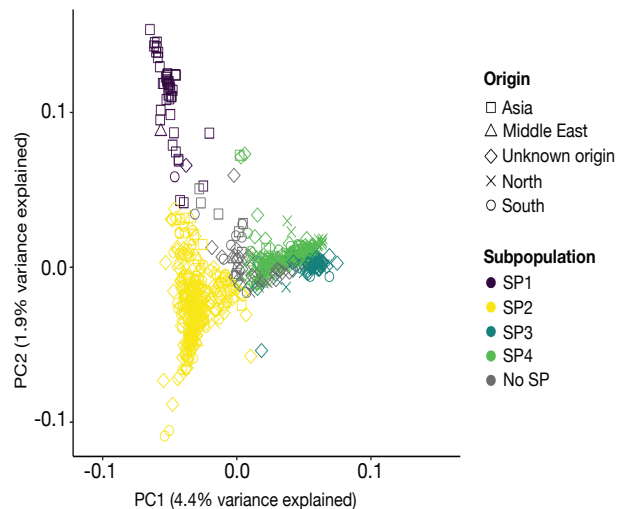

Supplement: Supplementary file 6 — Supplementary Figure 6. ADMIXTURE results. A) Cross-validation error of ADMIXTURE with K = 2 to K = 20. The bars display standard errors associated with repeating the CV 10 times for each value of K. B) ADMIXTURE proportions at K = 15 where the CV error is minimized. C–D) ADMIXTURE plots at K = 4. Each vertical bar represents a single accession colored by its ancestry proportions. Accessions are grouped according to their subpopulation membership (C) or by their geographic origin (D). E) Principal component analysis (PCA) based on genotypes. The ADMIXTURE subpopulations at K = 4 are represented by colors and geographic origin is represented by shapes. (PDF 1131 KB) [file 122_2023_4360_MOESM6_ESM.pdf]

**A**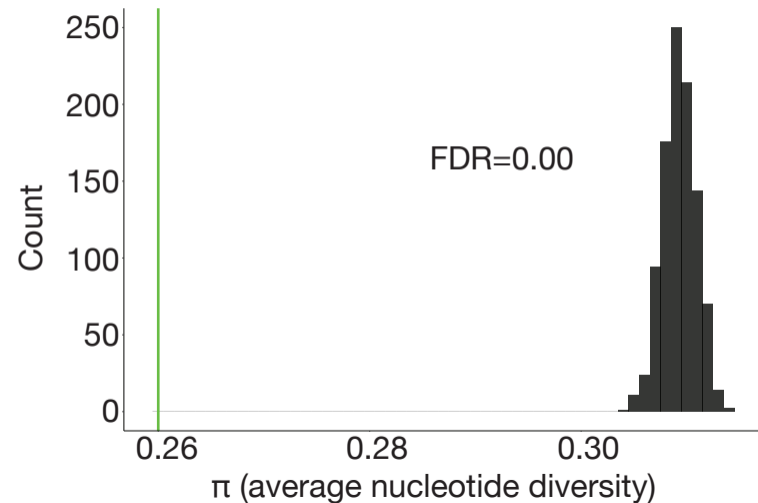**B**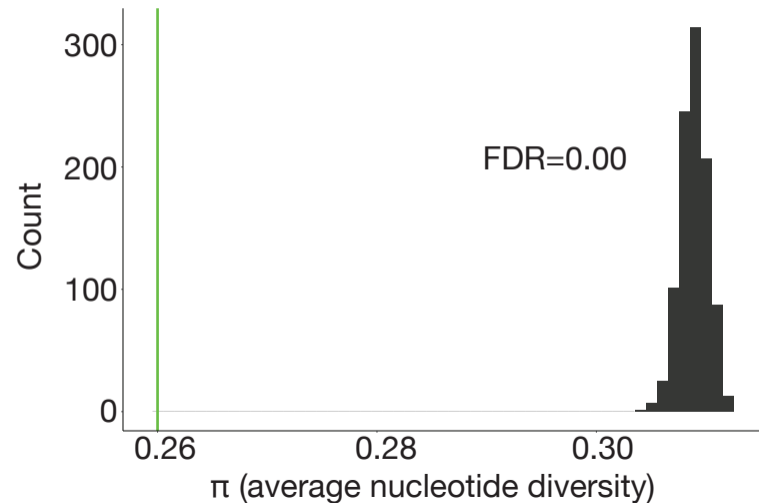

Supplement: Supplementary file 7 — Supplementary Figure 7. Distribution of average genome-wise nucleotide-diversity (π) values of 1000 subsets (n = 49) of SP1 (A) and SP2 (B). The horizontal green line displays the π value for SP3 (n = 49). (PDF 484 KB) [file 122_2023_4360_MOESM7_ESM.pdf]

SP1 vs. SP3

SP2 vs. SP3

Chr1S

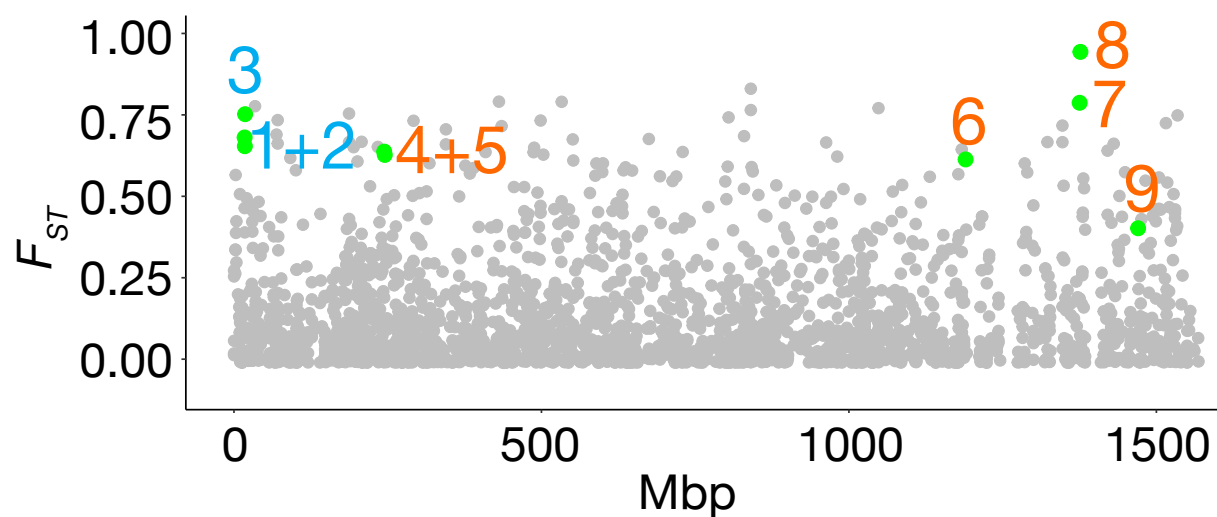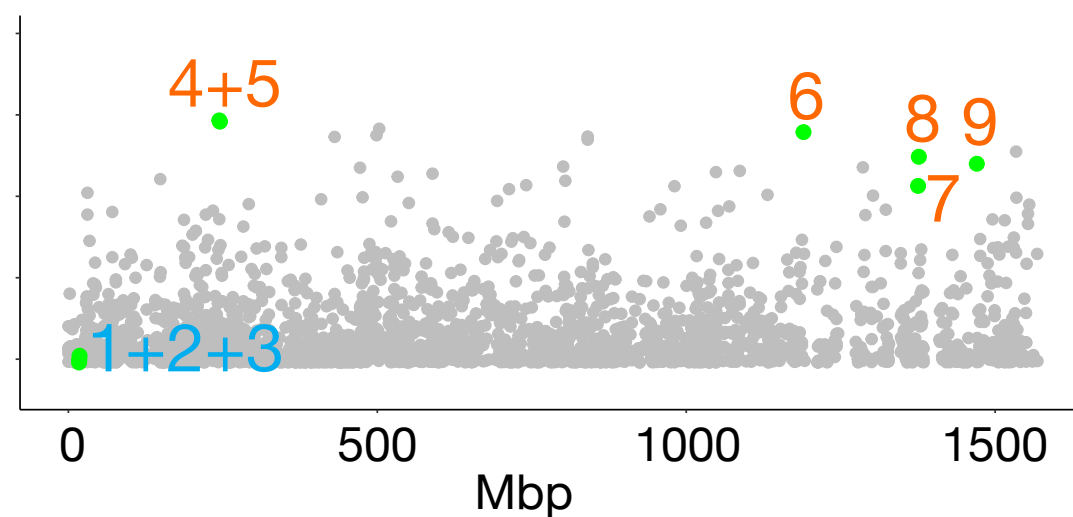

Chr1L

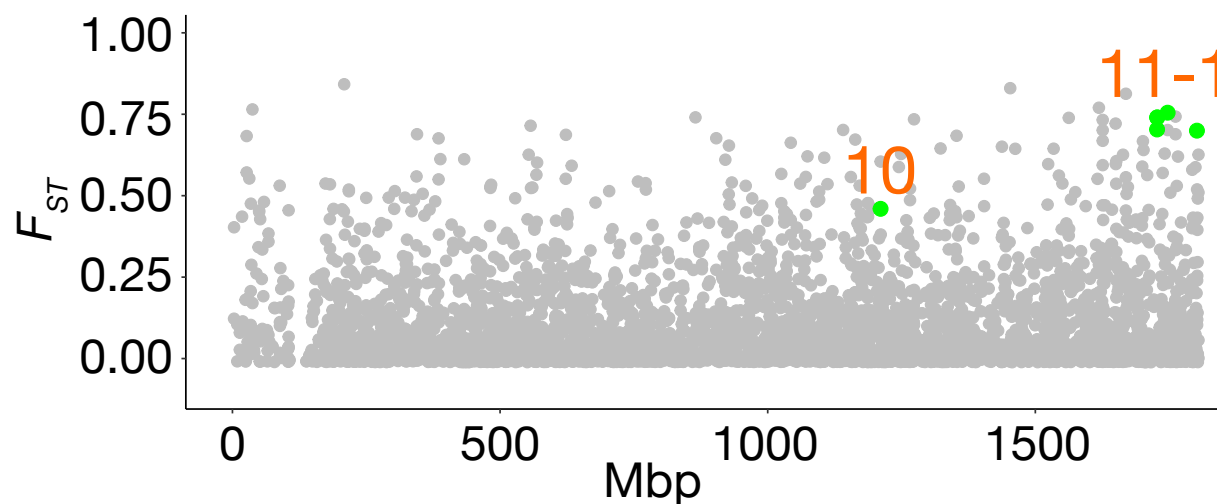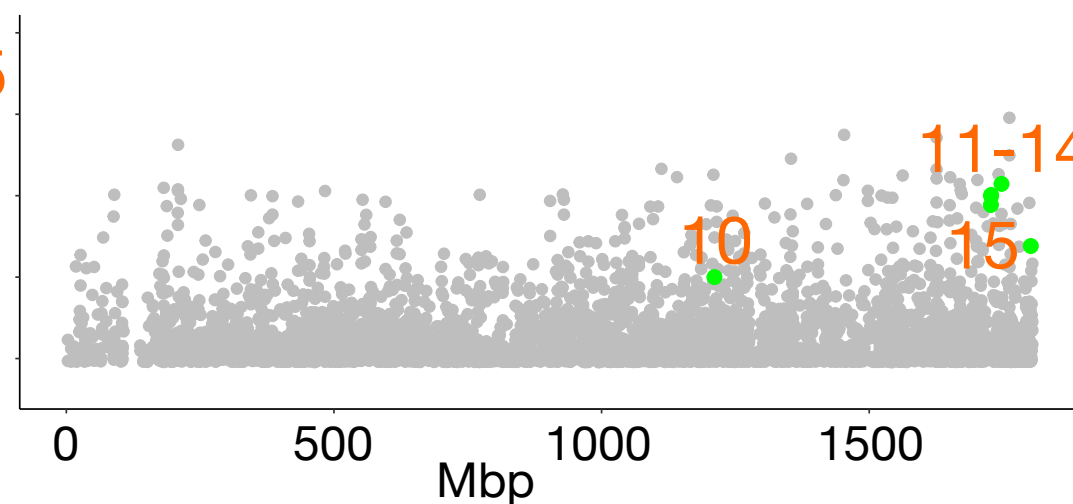

Chr2

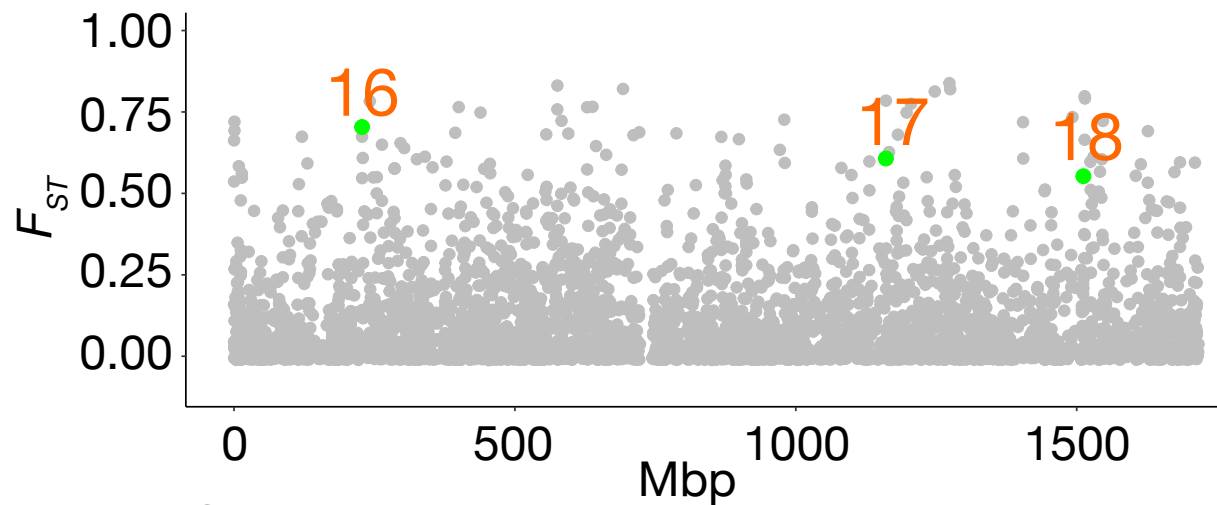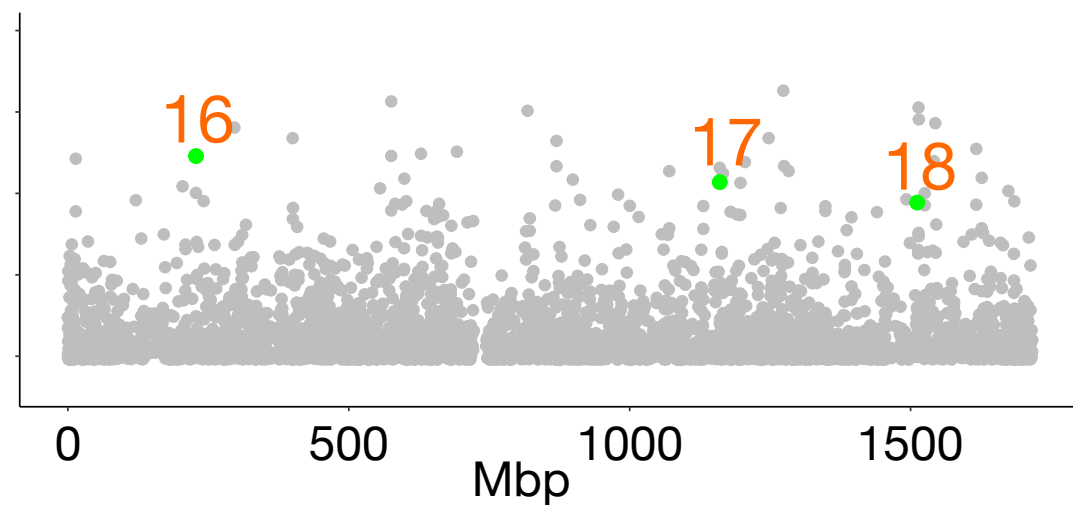

Chr3

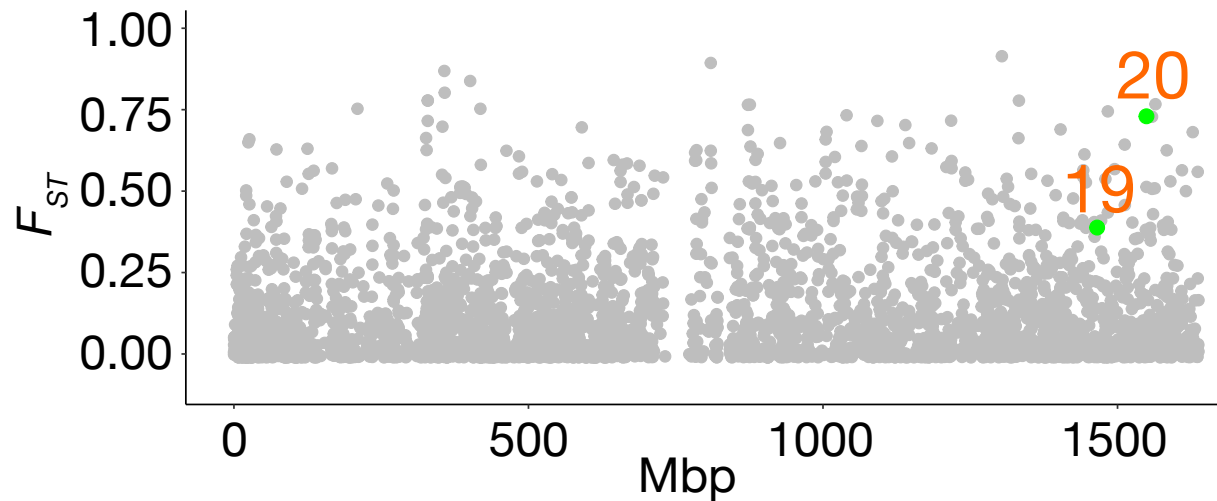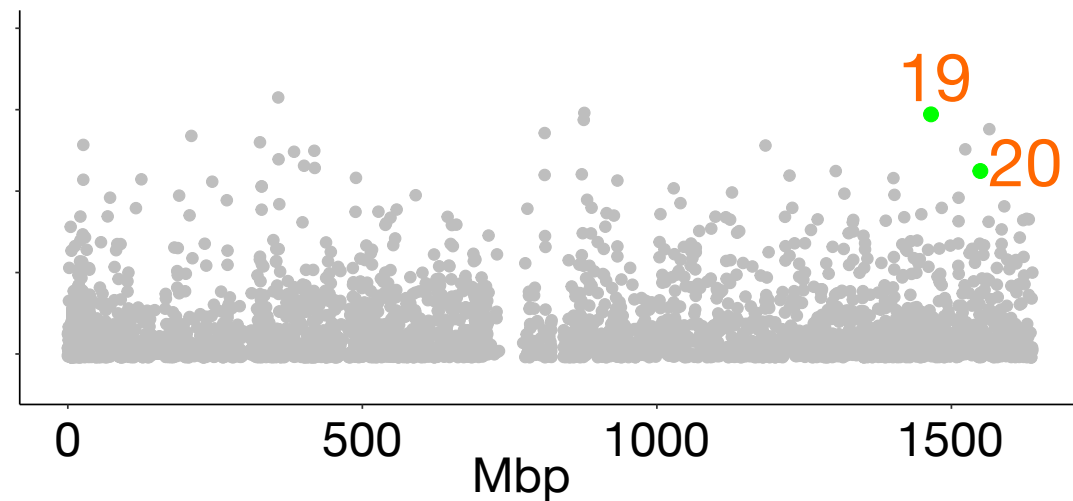

Chr4

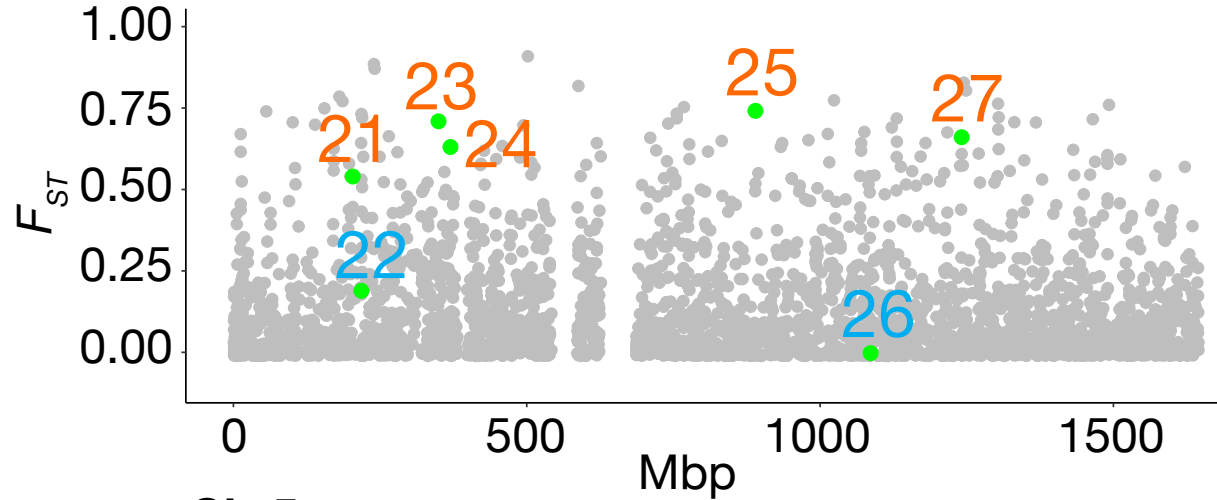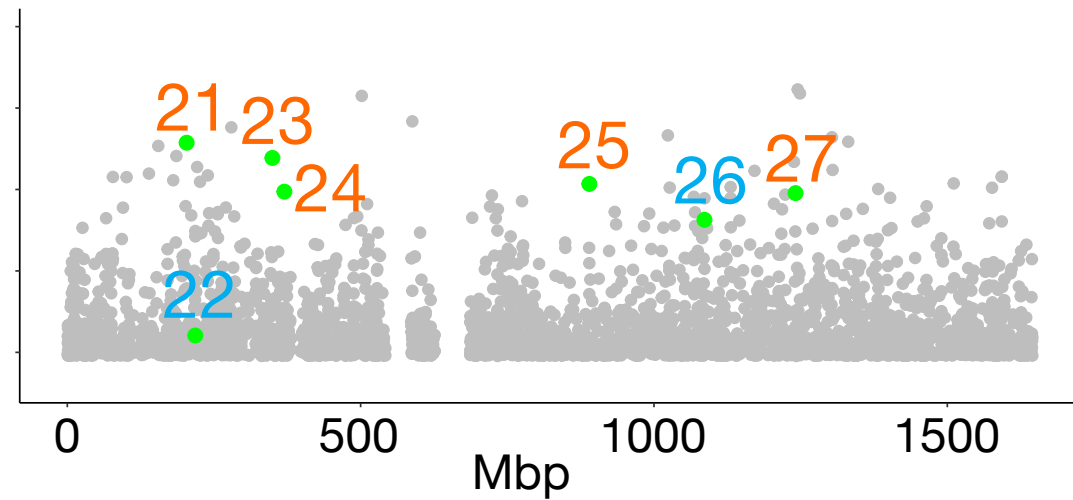

Chr5

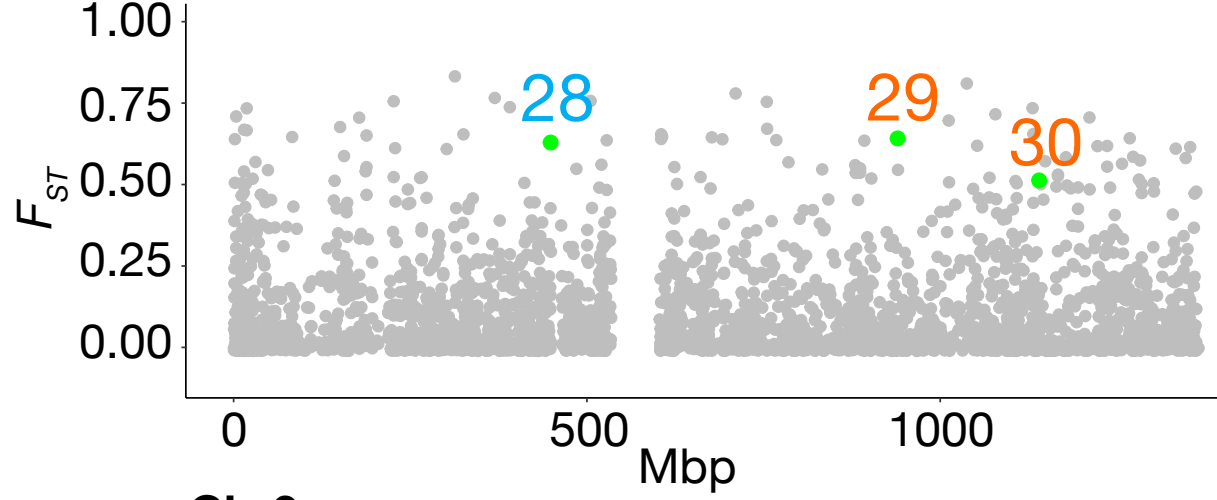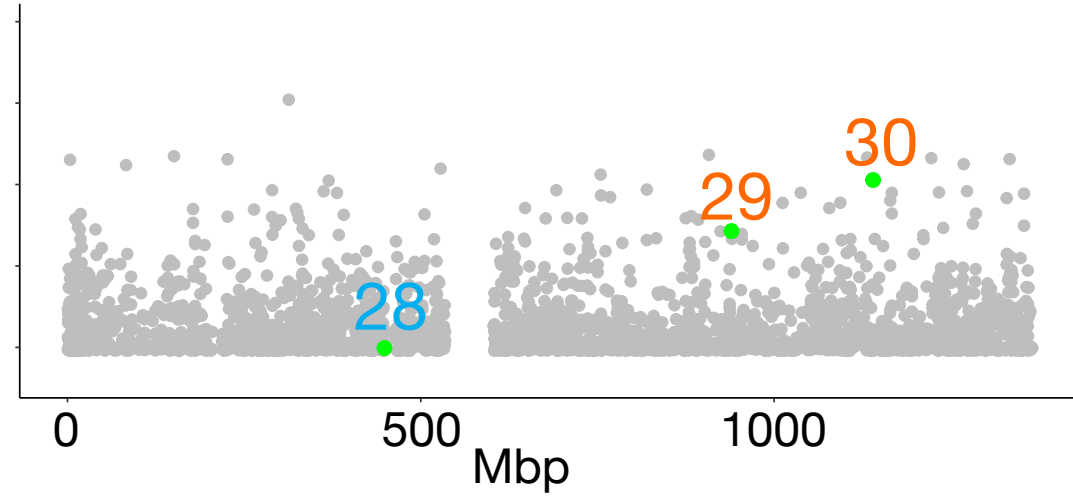

Chr6

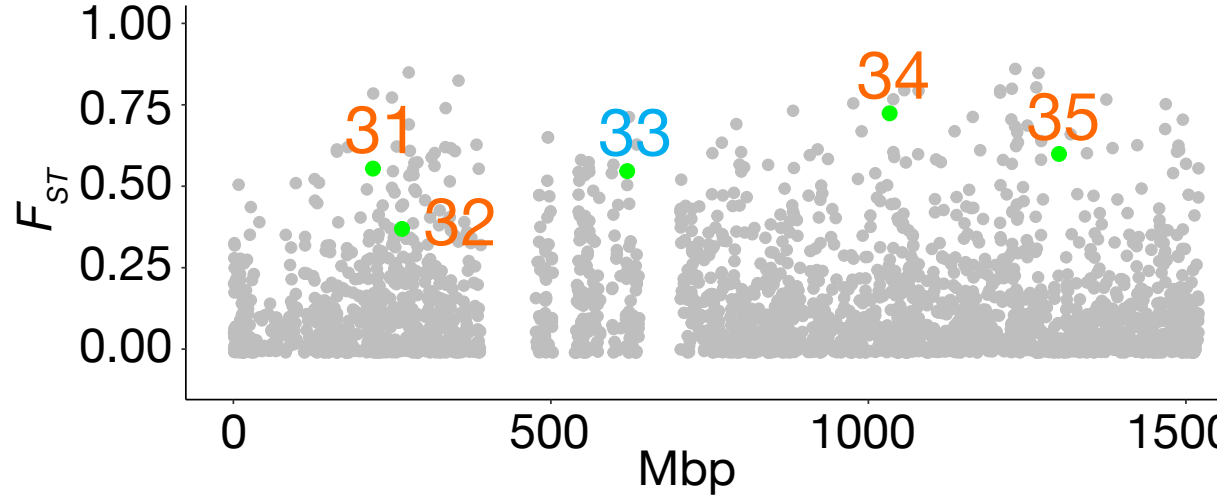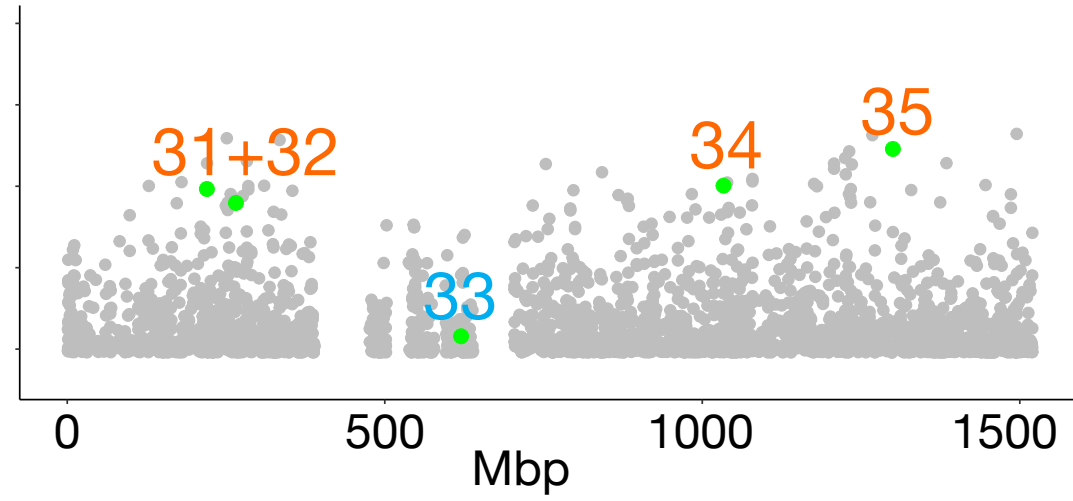

Supplement: Supplementary file 8 — Supplementary Figure 8. Genome-wide distribution of FST values for pairs of subpopulations. The FST values of each SNP throughout a chromosome are displayed as grey dots. The green dots report the 35 SNPs under selection identified in the outlier scans. The numbers next to the green dots serve as a marker code: 1: AX-416824401, 2: AX-416760427, 3: AX-416791399, 4: AX-416723873, 5: AX-416737096, 6: AX-416776470, 7: AX-181492359, 8: AX-416745027, 9: AX-416819371, 10: AX-416741889, 11: AX-181188041, 12: AX-181482613, 13: AX-416771656, 14: AX-416765862, 15: AX-181440418, 16: AX-181487950, 17: AX-181175939, 18: AX-181486832, 19: AX-181194098, 20: AX-416747475, 21: AX-416778737, 22: AX-416724016, 23: AX-416761735, 24: AX-181496895, 25: AX-416722420, 26: AX-181165197, 27: AX-416775196, 28: AX-416783057, 29: AX-416763147, 30: AX-416779502, 31: AX-416767699, 32: AX-181158030, 33: AX-181497981, 34: AX-416738786, 35: AX-181155942. Markers in LD group 1 are highlighted in blue. (PDF 747 KB) [file 122_2023_4360_MOESM8_ESM.pdf]

Subpopulation

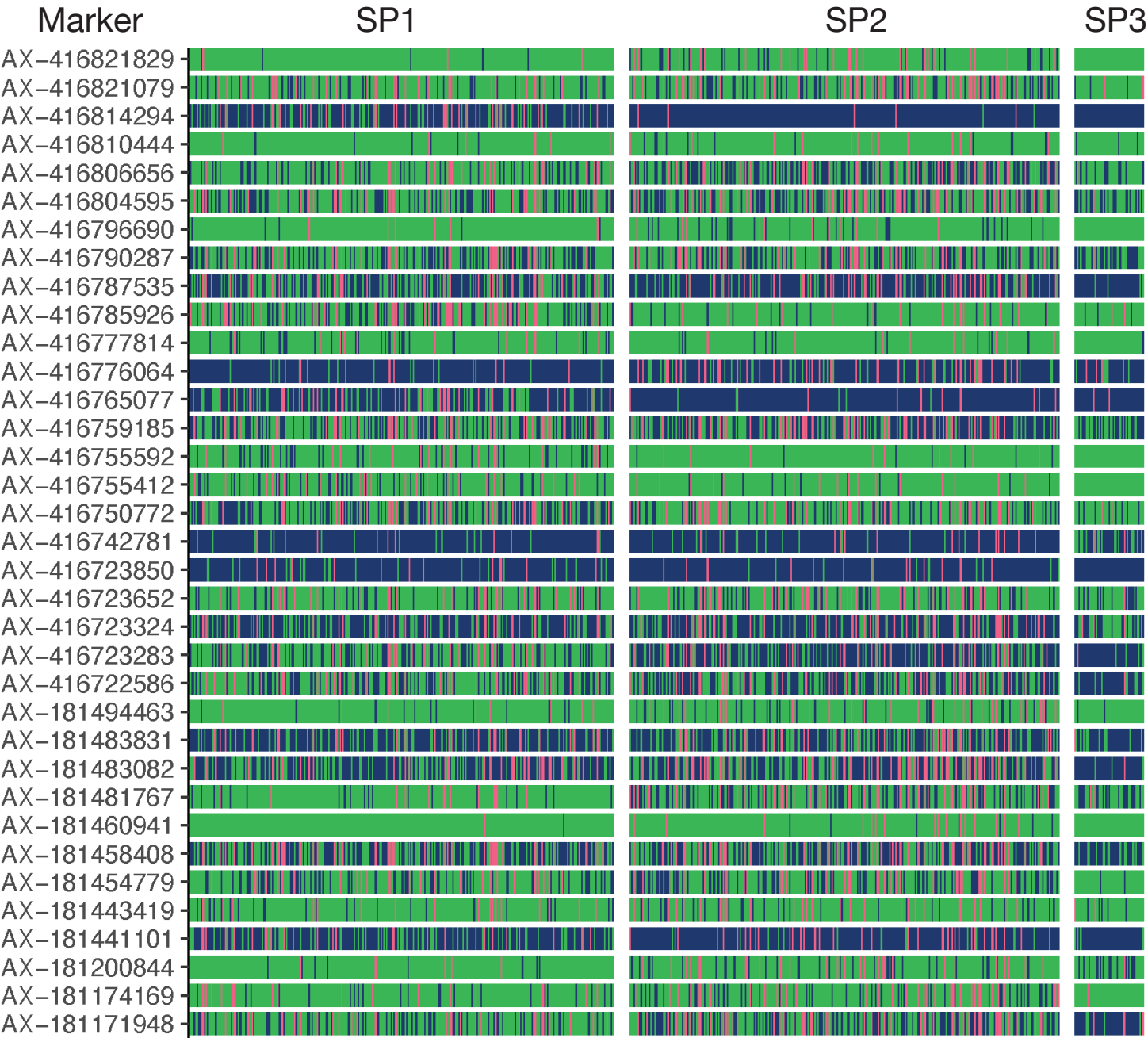

Supplement: Supplementary file 9 — Supplementary Figure 9. Segregation of 35 random markers. Each row shows the segregation pattern of one of 35 random markers. Each vertical line represents an accession and is colored by genotype for a specific marker. Genotype coloring scheme is as follows: green, reference homozygote; pink, heterozygote; blue, alternative homozygote. (PDF 621 KB) [file 122_2023_4360_MOESM9_ESM.pdf]

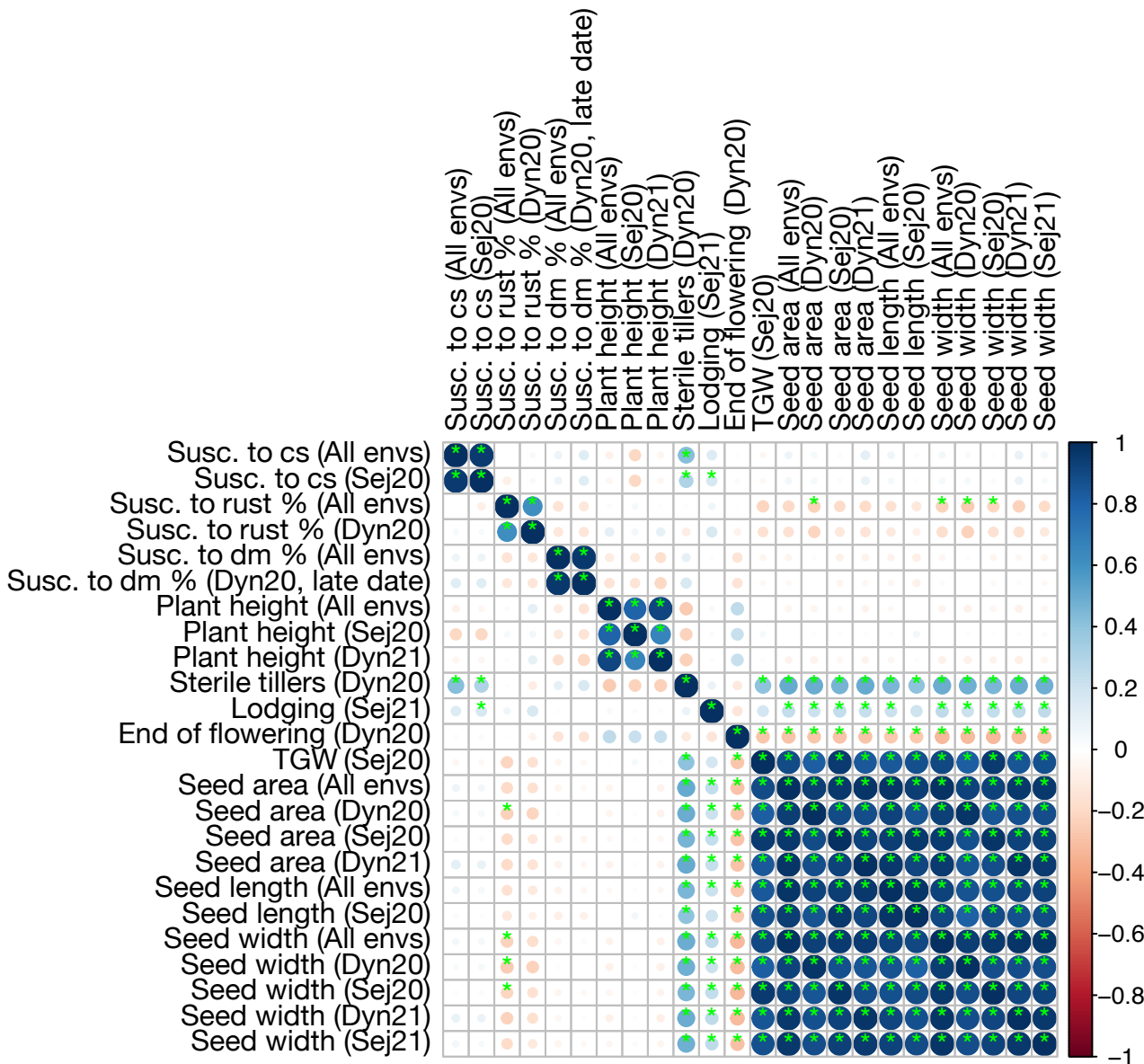

Supplement: Supplementary file 10 — Supplementary Figure 10. Genetic correlations between traits significantly explained by markers associated with North (SP1) versus South (SP2) differentiation. Green asterisk indicates statistical significance of correlation coefficients using a Bonferroni-corrected threshold of p < 0.05. Abbreviations: cha, character; cs, chocolate spot; dm, downy mildew; Dyn20, Dyngby 2020; Dyn21, Dyngby 2021; envs., environments; Sej20, Sejet 2020; Sej21, Sejet 2021; susc., susceptibility; TGW, Thousand grain weight. (PDF 350 KB) [file 122_2023_4360_MOESM10_ESM.pdf]

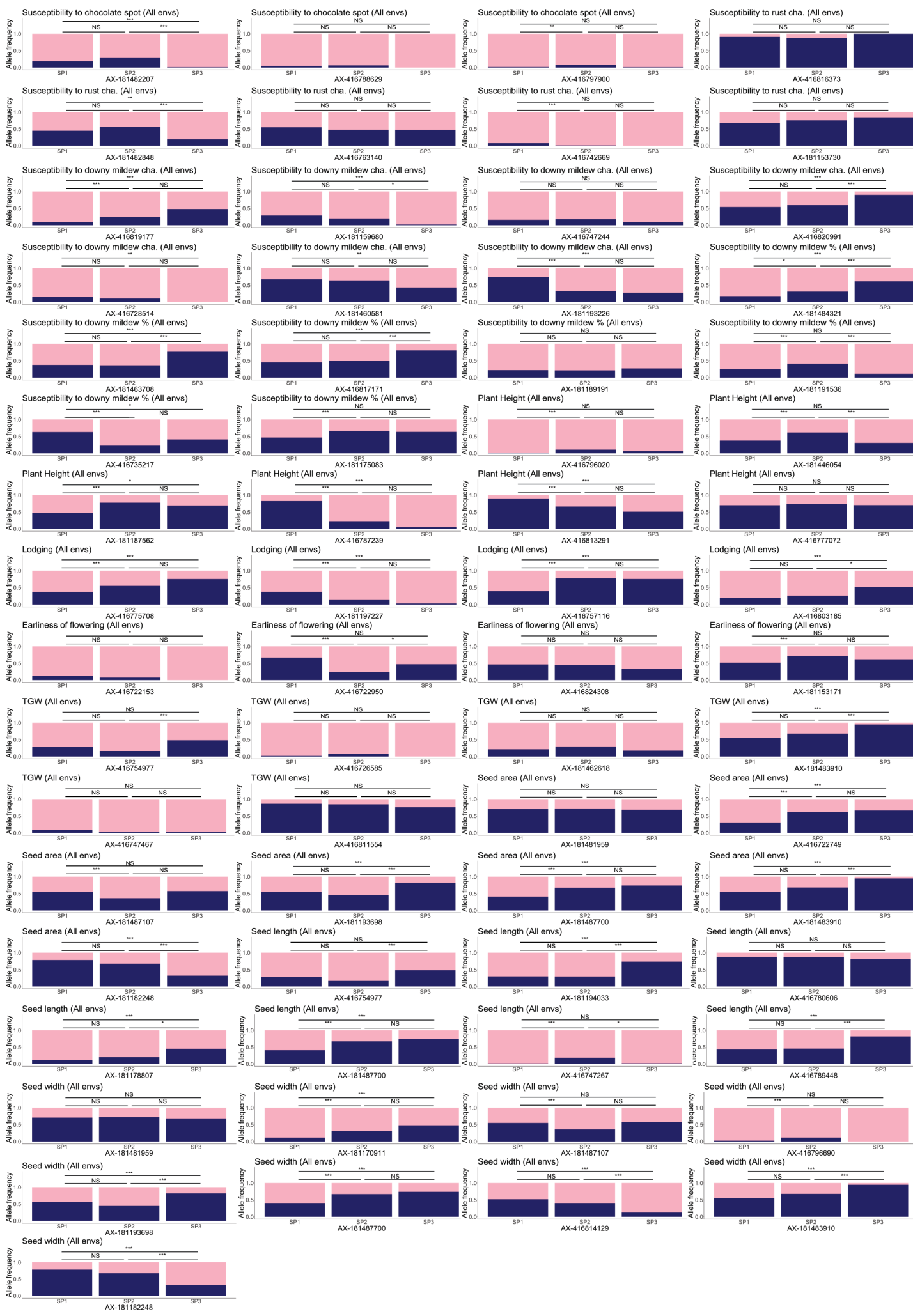

Supplement: Supplementary file 11 — Supplementary Figure 11. The subpopulation specific allele frequencies of the 65 stable QTLs identified in GWAS. Alleles associated with larger trait values in the GWAS population were defined as ‘positive’ (blue), whereas those associated with lower trait values were defined as ‘negative’ (pink). Asterisks indicate statistical significance of differential allele frequencies between subpopulations when corrected for multiple testing (Bonferroni correction) at p < 0.05 (*), p < 0.01 (**) and p < 0.005 significance levels. Abbreviations: cha, character; Dyn20, Dyngby 2020; Dyn21, Dyngby 2021; envs., environments; Sej20, Sejet 2020; Sej21, Sejet 2021; TGW, Thousand grain weight. (PDF 1028 KB) [file 122_2023_4360_MOESM11_ESM.pdf]

A

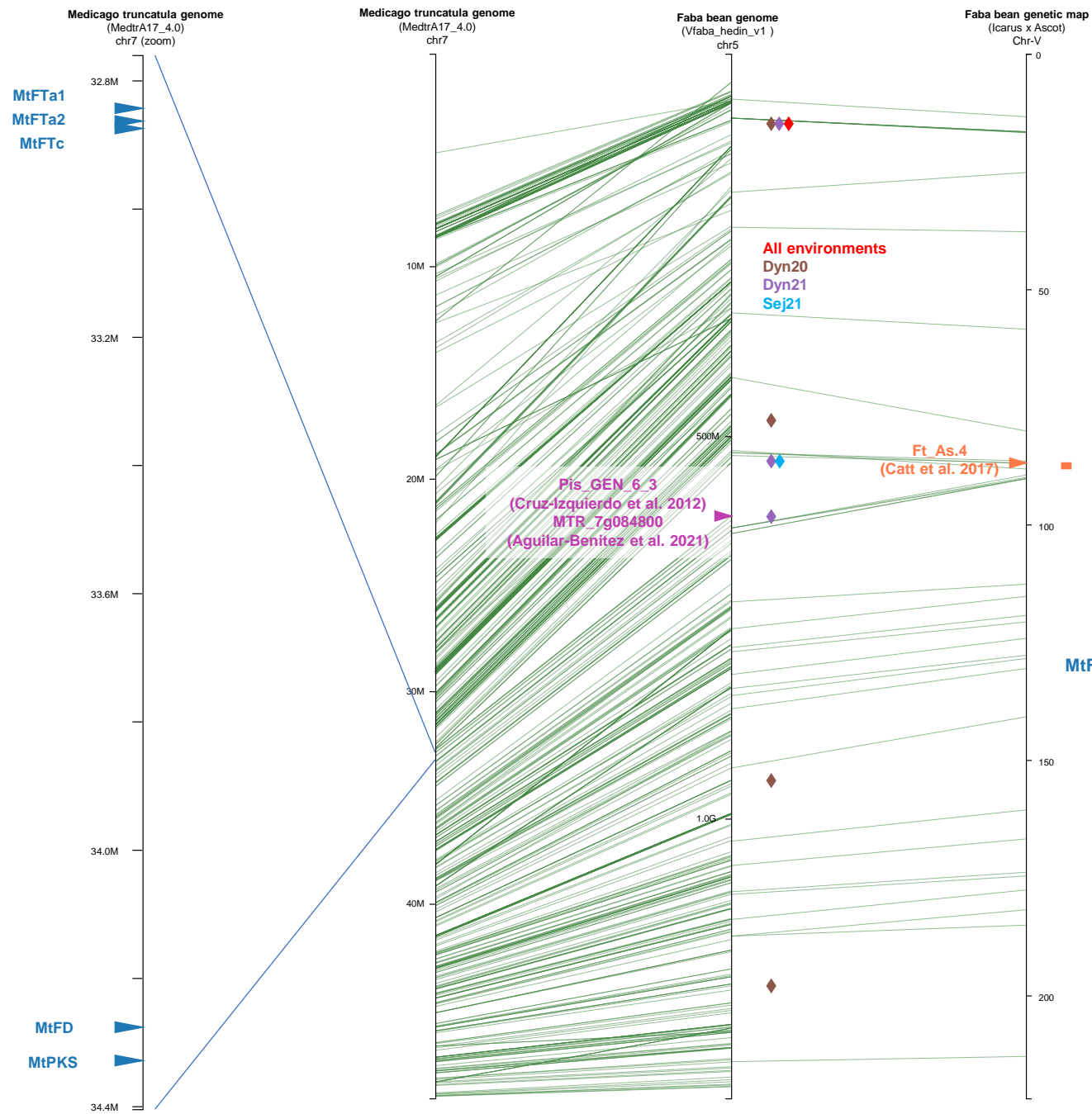

B

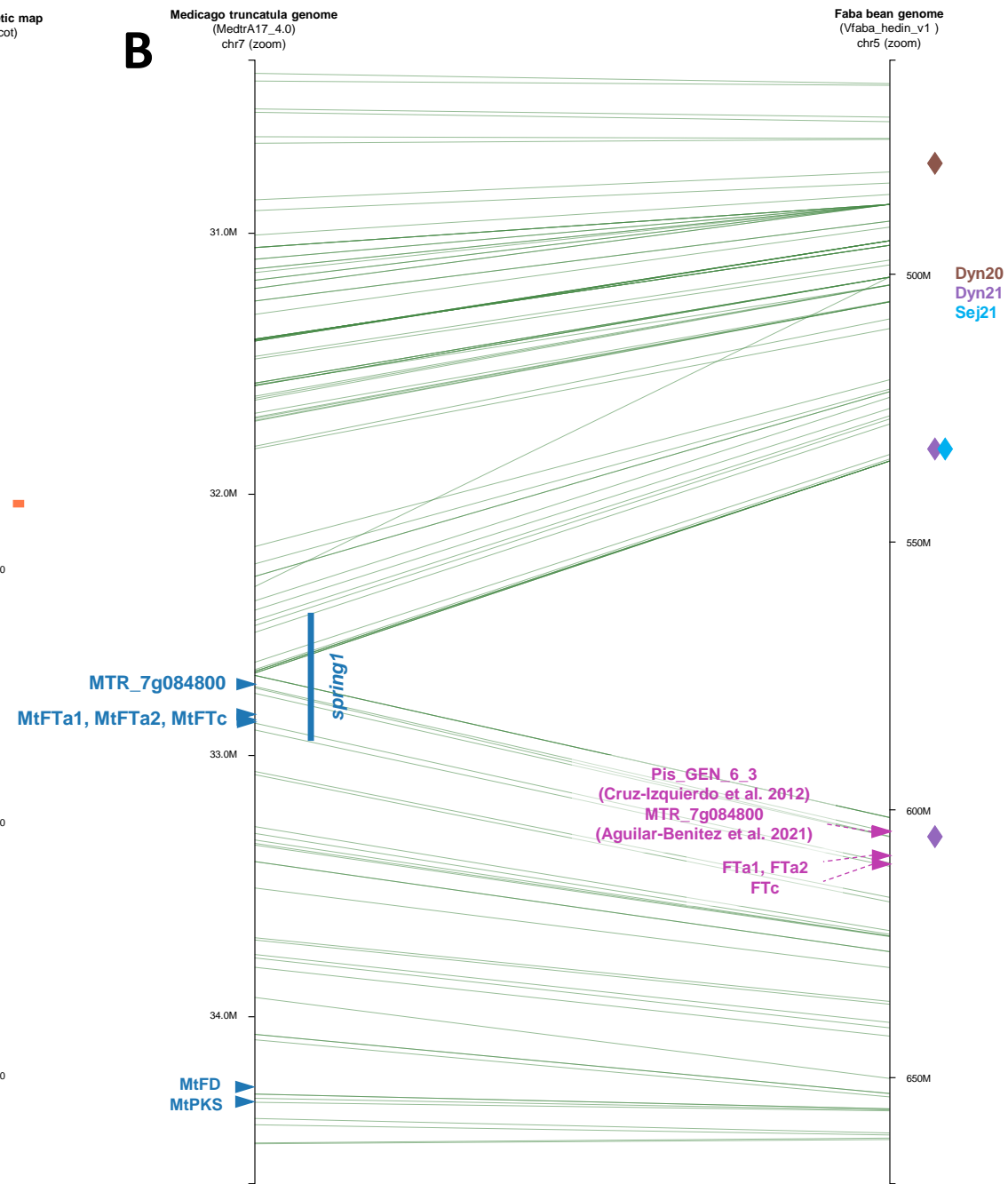

Supplement: Supplementary file 13 — Supplementary Figure 13. Synteny for QTLs related to flowering. A) A region on Medicago truncatula chromosome 7 containing five flowering time genes, defined in Yeoh et al. 2013 (zoomed in view, axis 1). The complete Medicago truncatula chromosome 7 projected across to faba bean chromosome 5 (axes 2 and 3). Green lines indicate gene mappings by sequence from Medicago truncatula to faba bean. The earliness of flowering QTLs from the present study are indicated by colored diamonds (all environments shown in red, Dyn20 in brown, Dyn21 in purple, Sej21 in light blue). The QTL from Catt et al. 2017 identified in the Icarus x Ascot genetic map (axis 4) is projected against faba bean chromosome 5 via mapped markers (green lines connecting the genetic map to physical sequence). B) Zoomed in view of the syntenic context around the spring1 locus in Medicago truncatula chromosome 7 (left axis) and corresponding region of faba bean chromosome 7 (right axis). The plot features 5 flowering time genes in Medicago truncatula (MtFTa1, MtFTa2, MtFTc, MtFD,MtPKS) located on chromosome 7. (PDF 73 KB) [file 122_2023_4360_MOESM13_ESM.pdf]
