# Supplementary material for: Genetic analysis of global faba bean diversity, agronomic traits and selection signatures
Source: Theor Appl Genet. 2023 Apr 19;136(5):114. doi: 10.1007/s00122-023-04360-8 (PMC10115707; doi:10.1007/s00122-023-04360-8)

Herbicide damage (Dyn21)

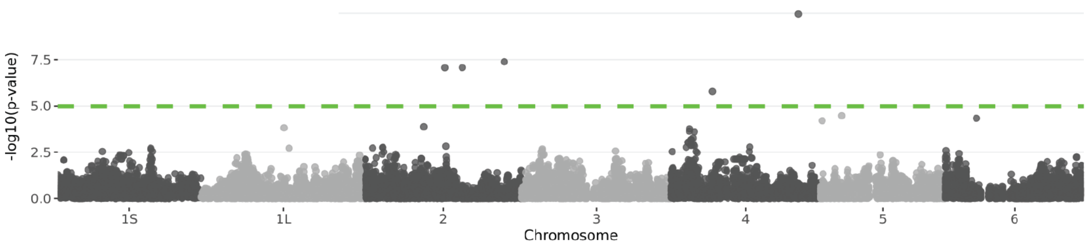

Branching (Dyn20)

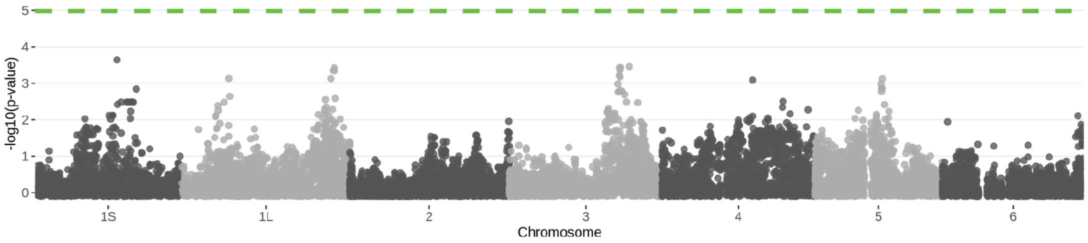

Number of ovules (Dyn20)

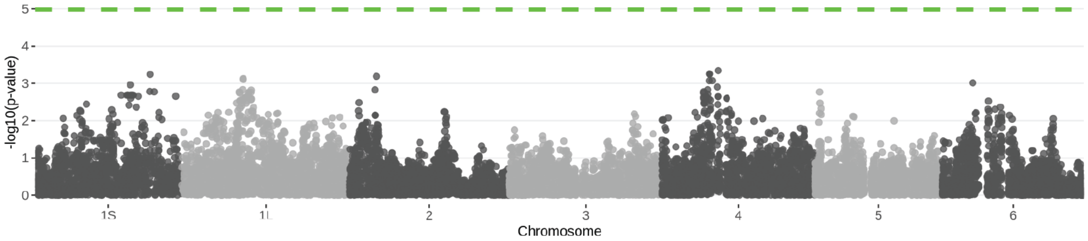

Sterile tillers (Dyn20)

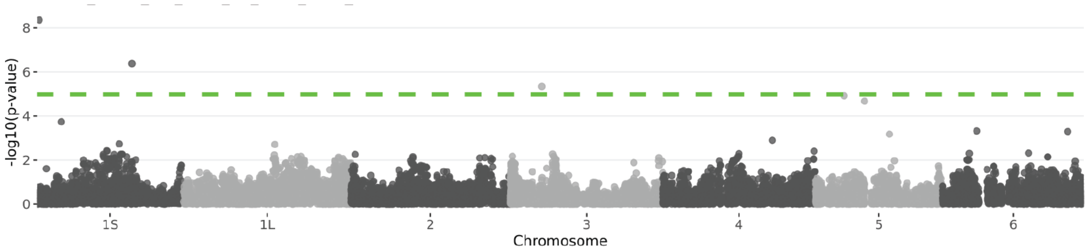

Maturation (Dyn20)

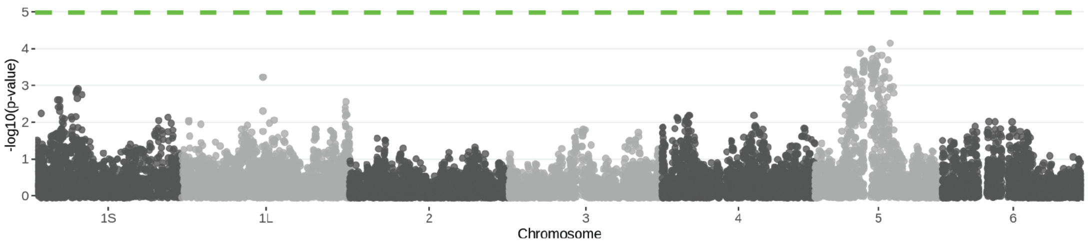

End of flowering (Dyn20)

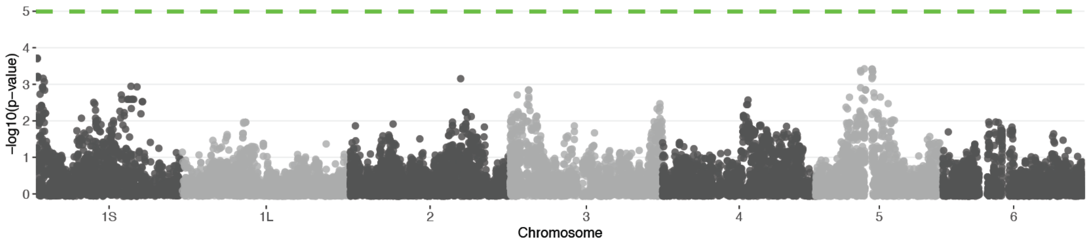

Duration of flowering (Dyn20)

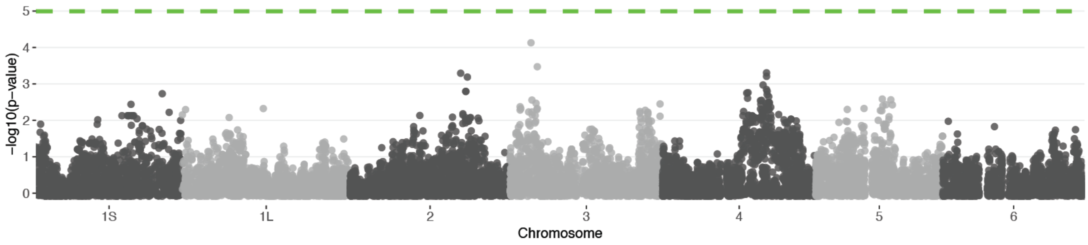

Supplement: Supplementary file 5 — Supplementary Figure 5. Additional Manhattan plots. Manhattan plots for GWAS of herbicide damage, branching, number of ovules, number of sterile tillers per plant, maturation date, end of flowering, and duration of flowering. The green line indicates the SimpleM-corrected threshold for significance. Abbreviations: Dyn20, Dyngby 2020; Dyn21, Dyngby 2021. (PDF 902 KB) [file 122_2023_4360_MOESM5_ESM.pdf]
