# Supplementary material for: Genetic analysis of global faba bean diversity, agronomic traits and selection signatures
Source: Theor Appl Genet. 2023 Apr 19;136(5):114. doi: 10.1007/s00122-023-04360-8 (PMC10115707; doi:10.1007/s00122-023-04360-8)
Supplement: Supplementary file 12 — Supplementary Figure 12. Synteny for QTLs related to seed size. A) Mapped QTLs for seed area, seed width, seed length and TGW on chromosome 2 (whole chromosome, left axis) and zoomed region (right side). Markers defining the QTL interval on chromosome 2 reported for seed weight in Khazaei et al. 2014 are shown in blue. B) Mapped QTLs for seed area, seed width, seed length and TGW on chromosome 4 (whole chromosome, left axis) and zoomed region (right side). Markers defining the QTL interval on chromosome 4 reported for seed weight in Khazaei et al. 2014 are shown in blue. (PDF 74 KB) [file 122_2023_4360_MOESM12_ESM.pdf]

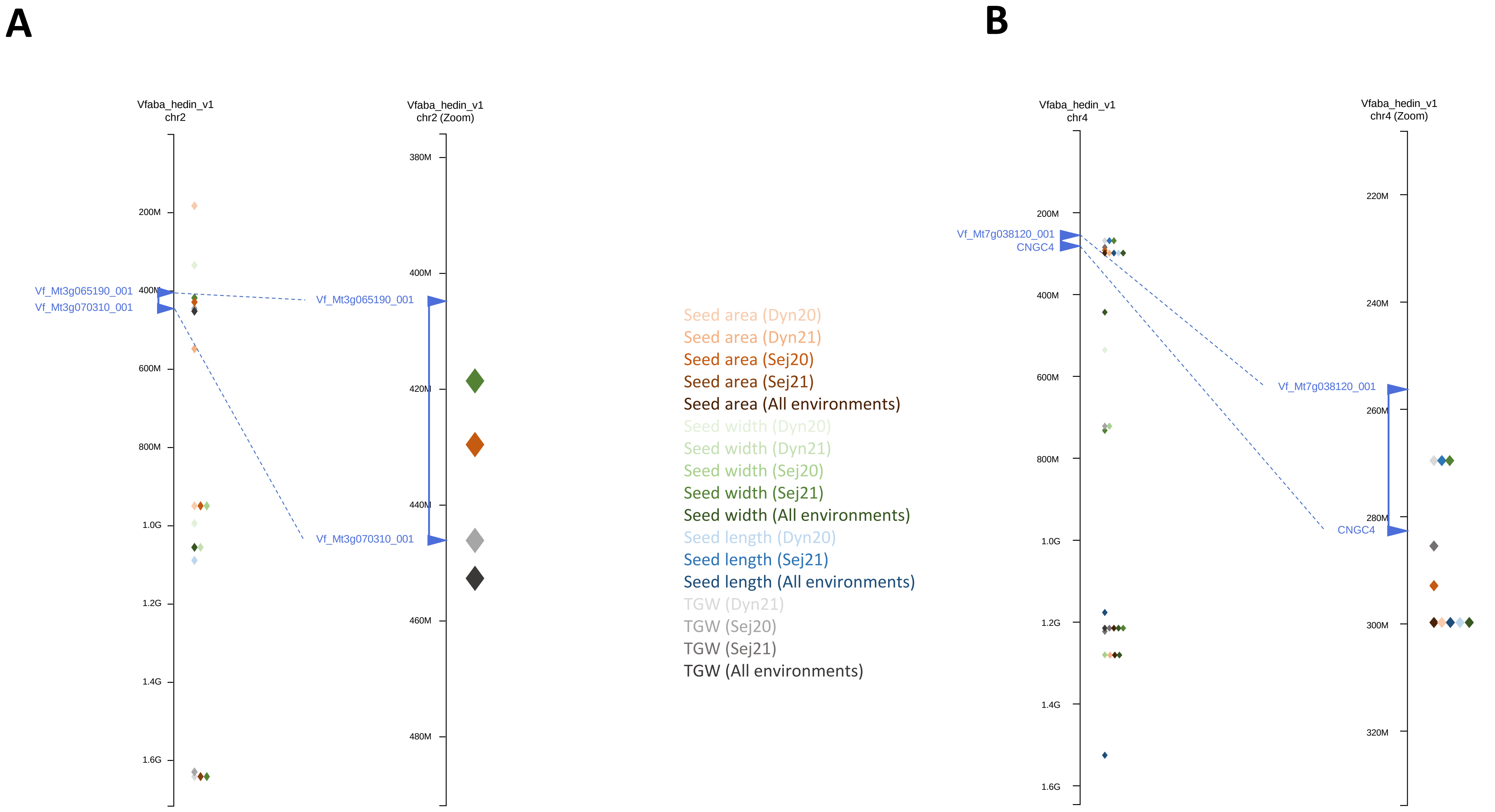
A
Vfaba_hedin_v1
chr2
200M
400M
600M
800M
1.0G
1.2G
1.4G
1.6G
Vf_Mt3g065190_001
Vf_Mt3g070310_001
Vfaba_hedin_v1
chr2 (Zoom)
380M
400M
420M
440M
460M
480M
Vf_Mt3g065190_001
Vf_Mt3g070310_001
Seed area (Dyn20)
Seed area (Dyn21)
Seed area (Sej20)
Seed area (Sej21)
Seed area (All environments)
Seed width (Dyn20)
Seed width (Dyn21)
Seed width (Sej20)
Seed width (Sej21)
Seed width (All environments)
Seed length (Dyn20)
Seed length (Sej21)
Seed length (All environments)
TGW (Dyn21)
TGW (Sej20)
TGW (Sej21)
TGW (All environments)
B
Vfaba_hedin_v1
chr4
200M
400M
600M
800M
1.0G
1.2G
1.4G
1.6G
Vf_Mt7g038120_001
CNGC4
Vfaba_hedin_v1
chr4 (Zoom)
220M
240M
260M
280M
300M
320M
Vf_Mt7g038120_001
CNGC4
