# Supplementary material for: Genetic analysis of global faba bean diversity, agronomic traits and selection signatures
Source: Theor Appl Genet. 2023 Apr 19;136(5):114. doi: 10.1007/s00122-023-04360-8 (PMC10115707; doi:10.1007/s00122-023-04360-8)

Pretzel instructions

1. Log in

Access Pretzel Pulses URL: https://pulses.plantinformatics.io/
Click Log In in top right

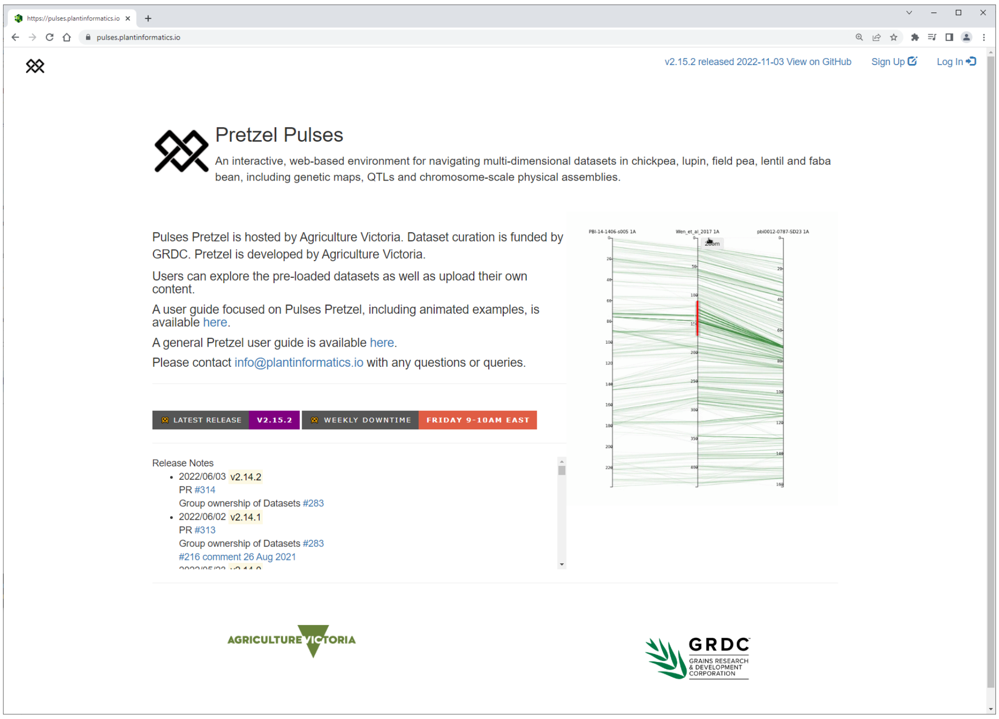

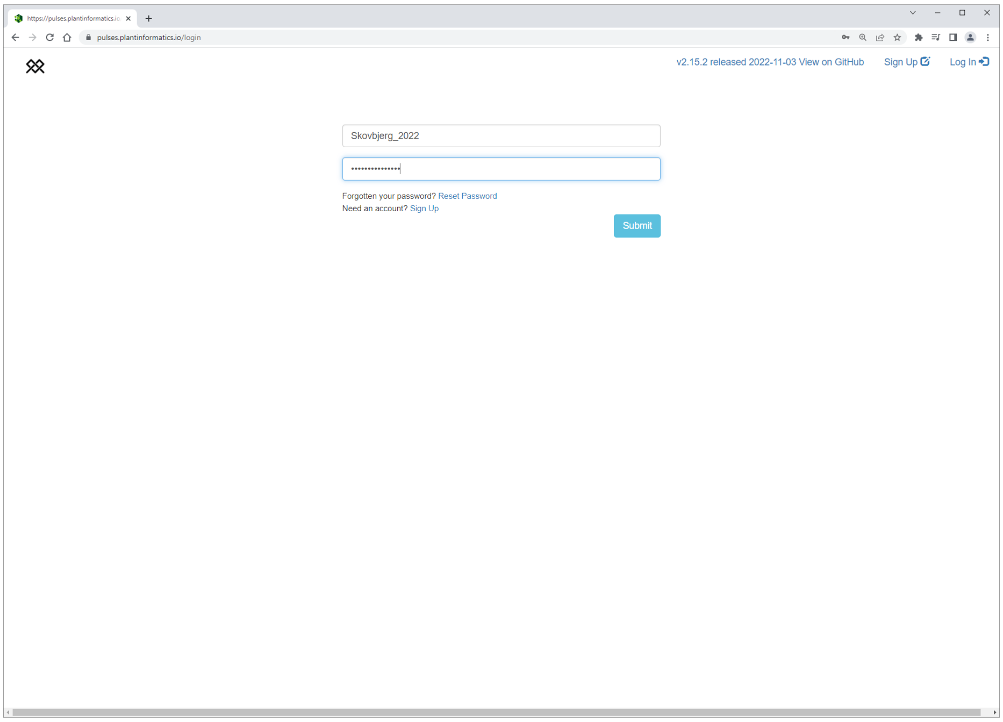

Log in with the following details:

Username: Skovbjerg_2022
Password: FabaBean&ABC123

2. Load datasets

Scroll down the dataset explorer on the left of the screen to select the following datasets:

- Vfaba_hedin_v1.MedtrA17_4.0_mapping – chr5

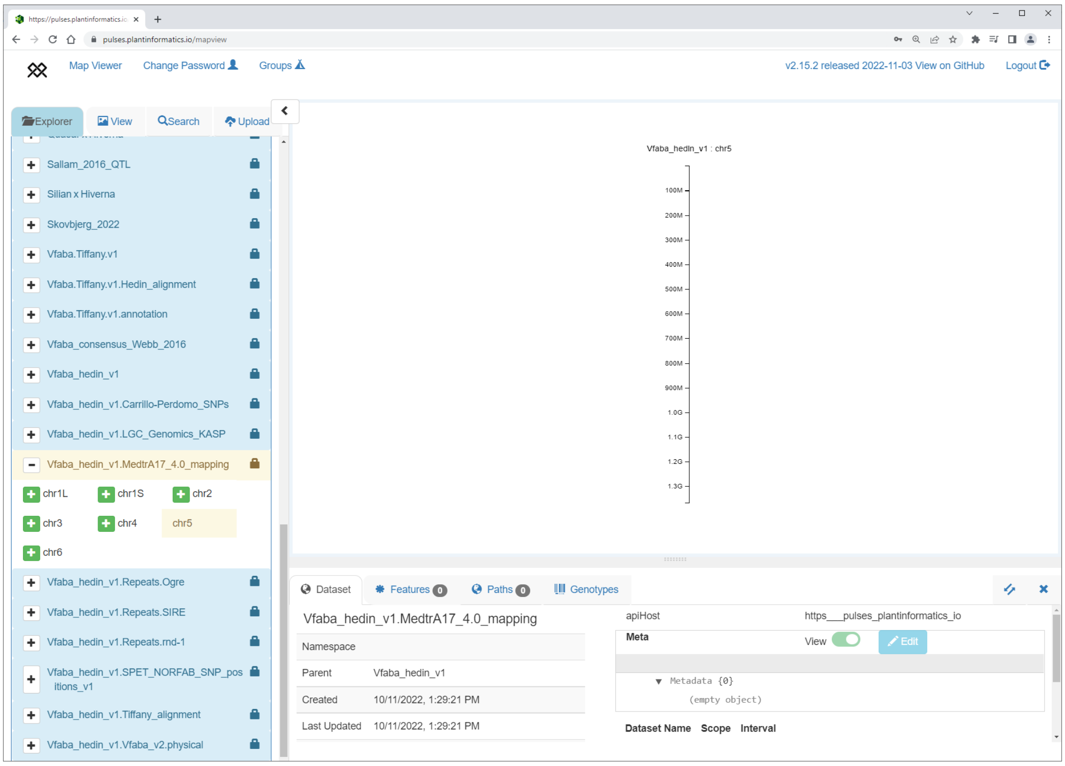

- Medicago_truncatula.MedtrA17_4.0.annotation – chr7

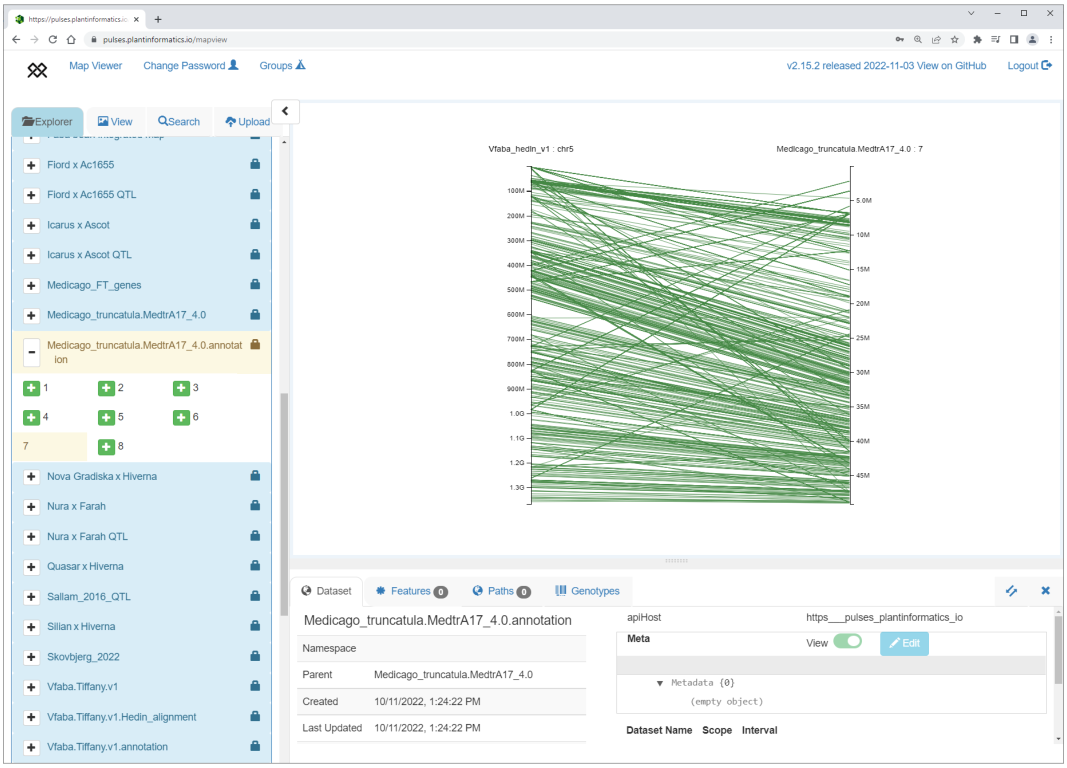

- Skovbjerg_2022 – chr5

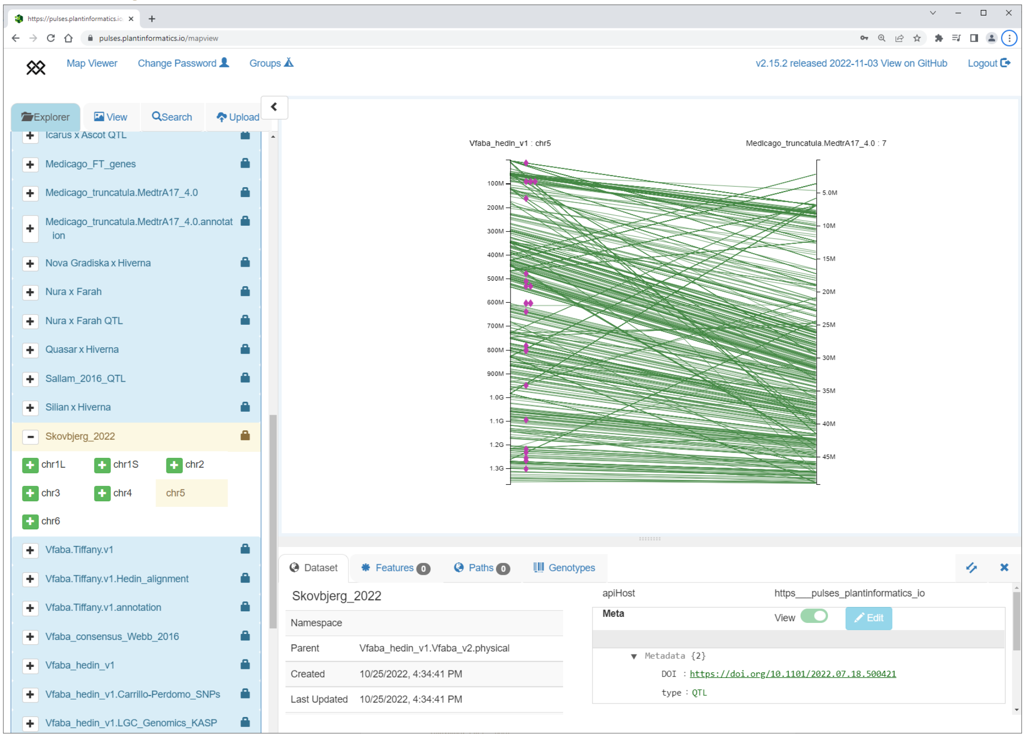

- 20210909_Fababean_SNP_SSR_Seq_Info – chr5
- Icarus x Ascot QTL – Chr-V

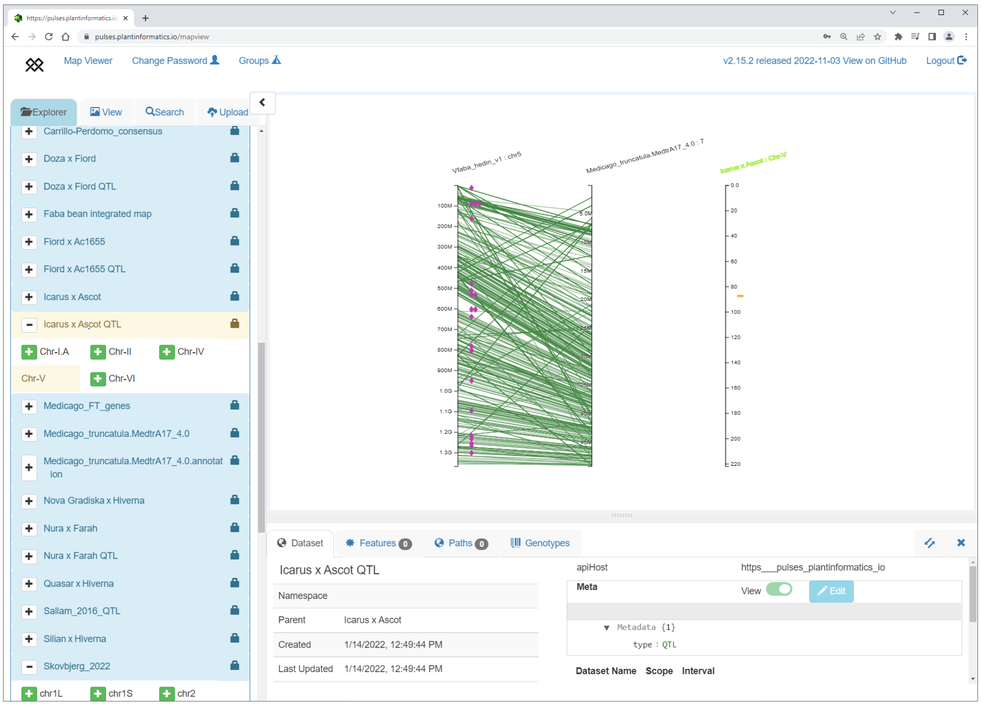

- Medicago_FT_genes - 7

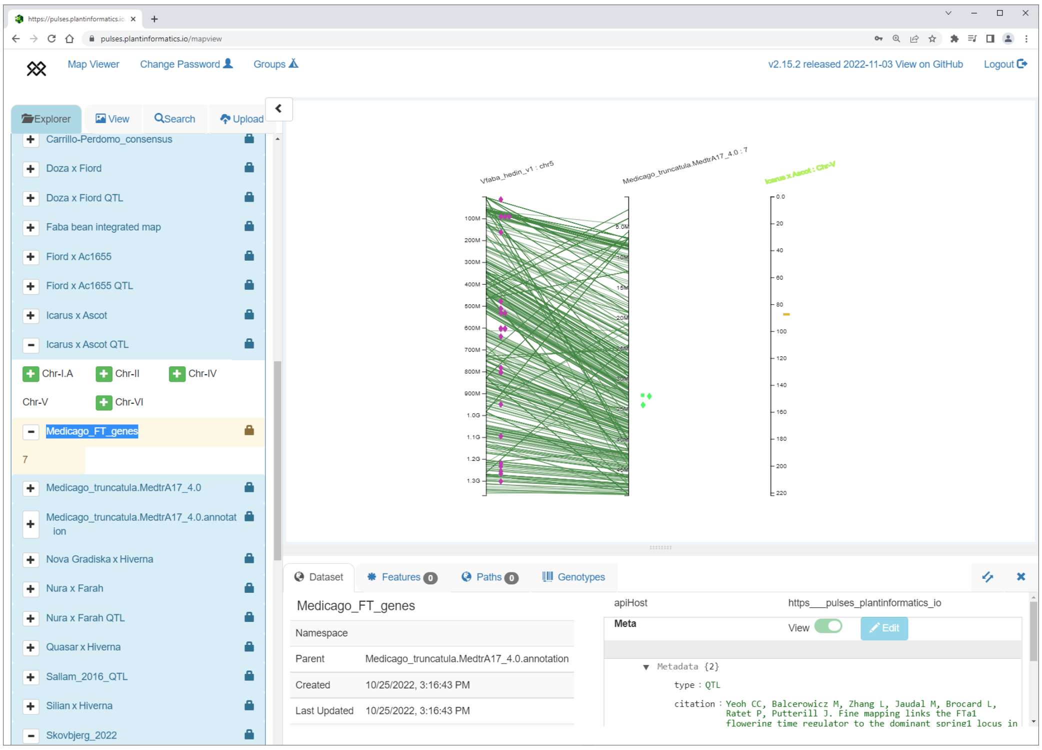

3. Re-arrange axes

Hold the CTRL key, click and drag on the Medicago axis to move it to the left side.

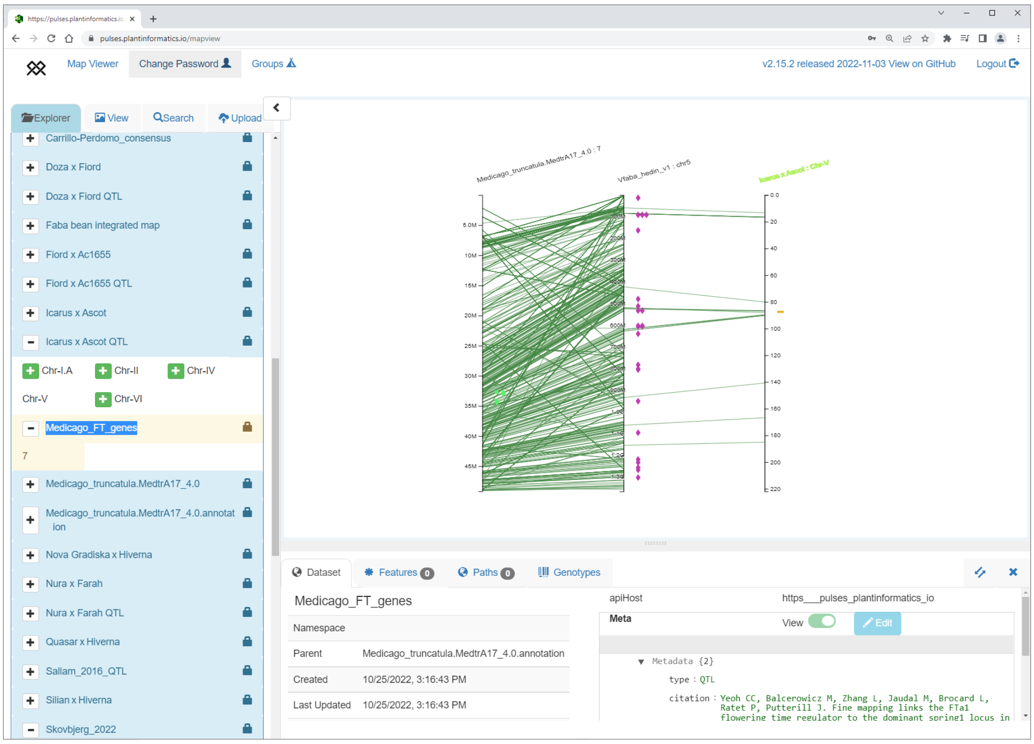

4. Toggle displayed traits

Click on the View tab on the left side panel.

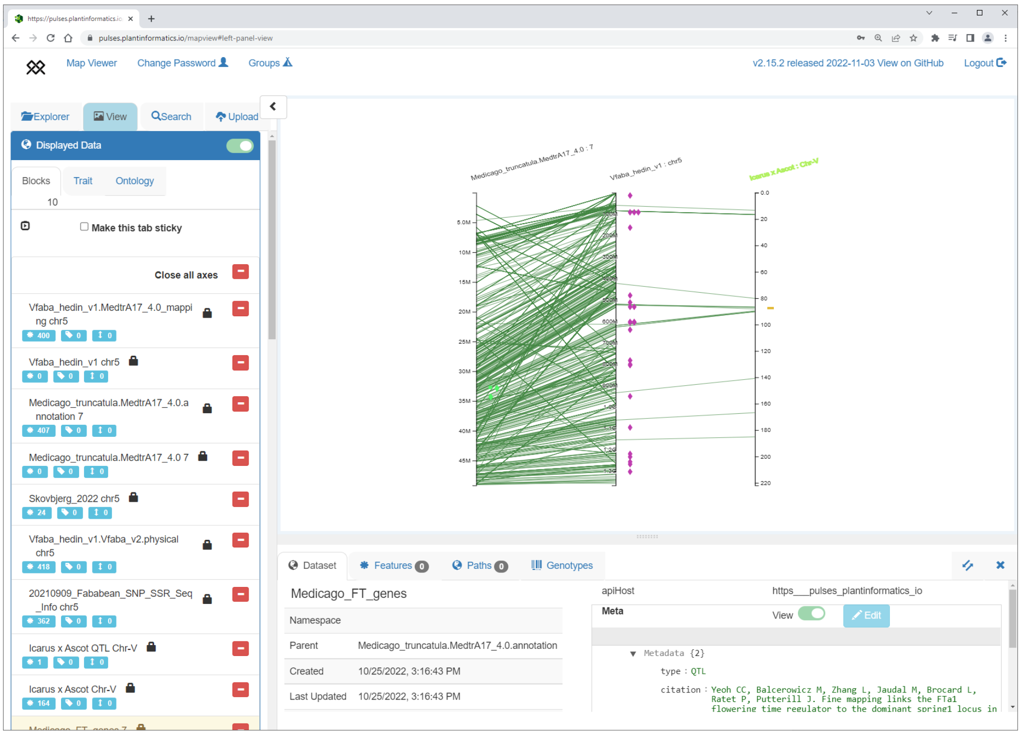

Within the Displayed Data sub-panel, select the Trait tab at the top.

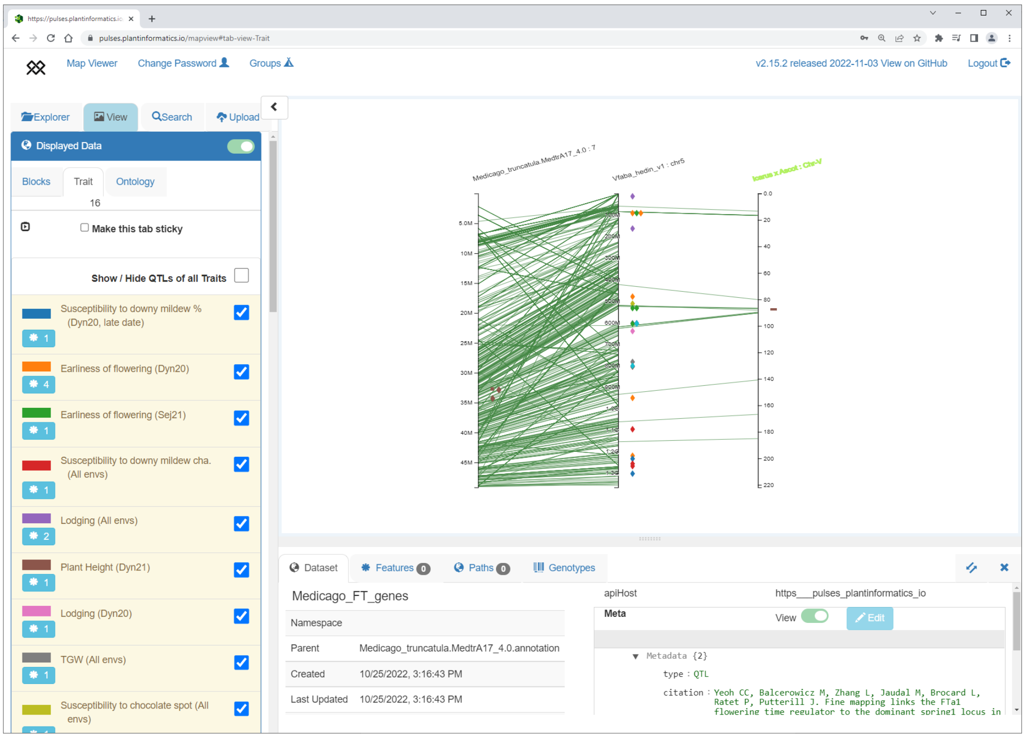

This colours the QTL symbols according to trait. Mouse over any trait symbols for detail on that QTL.

5. Brush (select) a region of an axis

Move the mouse over the axis, click and drag up or down to “brush” (make a selection on) the axis.

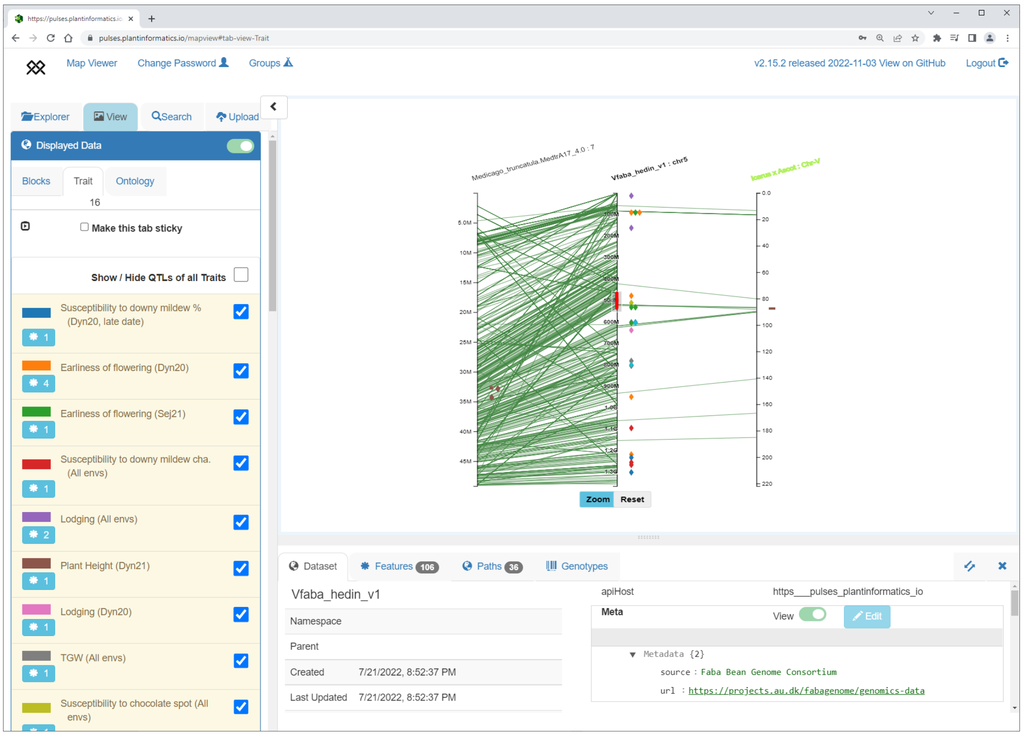

The selected region will be indicated by a grey rectangle and underlying features shown in red.

6. Zoom on selected region

Click the Zoom button to zoom the axis to the selected region.

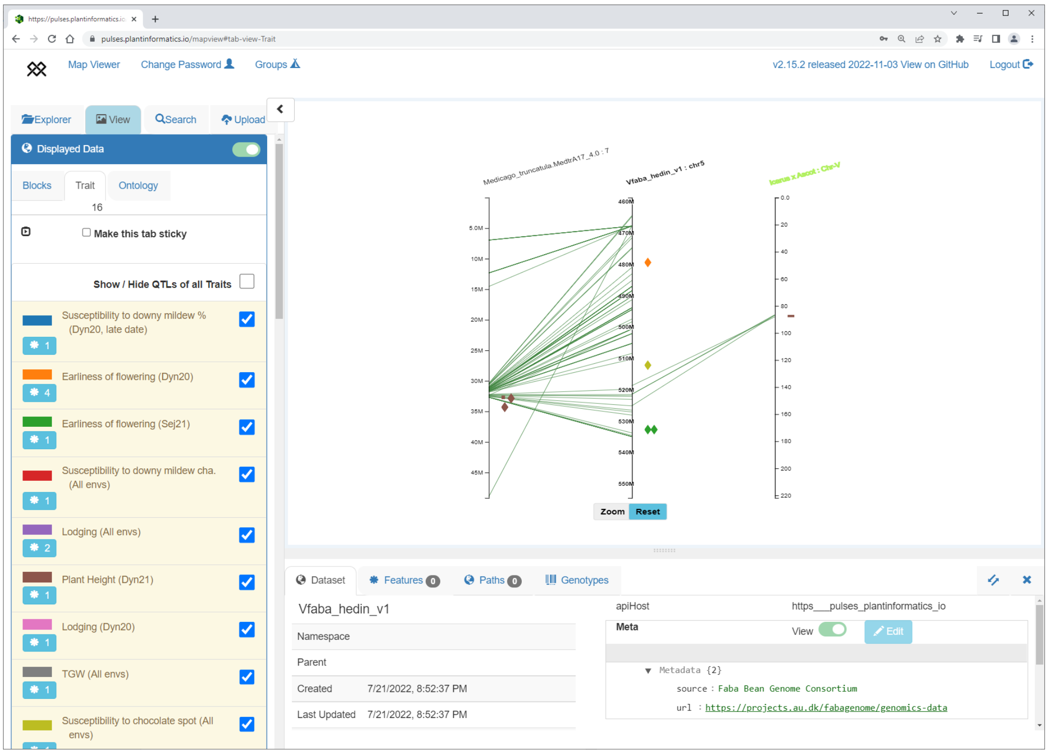

Reset the zoom by clicking the Reset button.

7. Tweak visualisation options

In the View tab of the left panel, scroll down to access visualisation options.

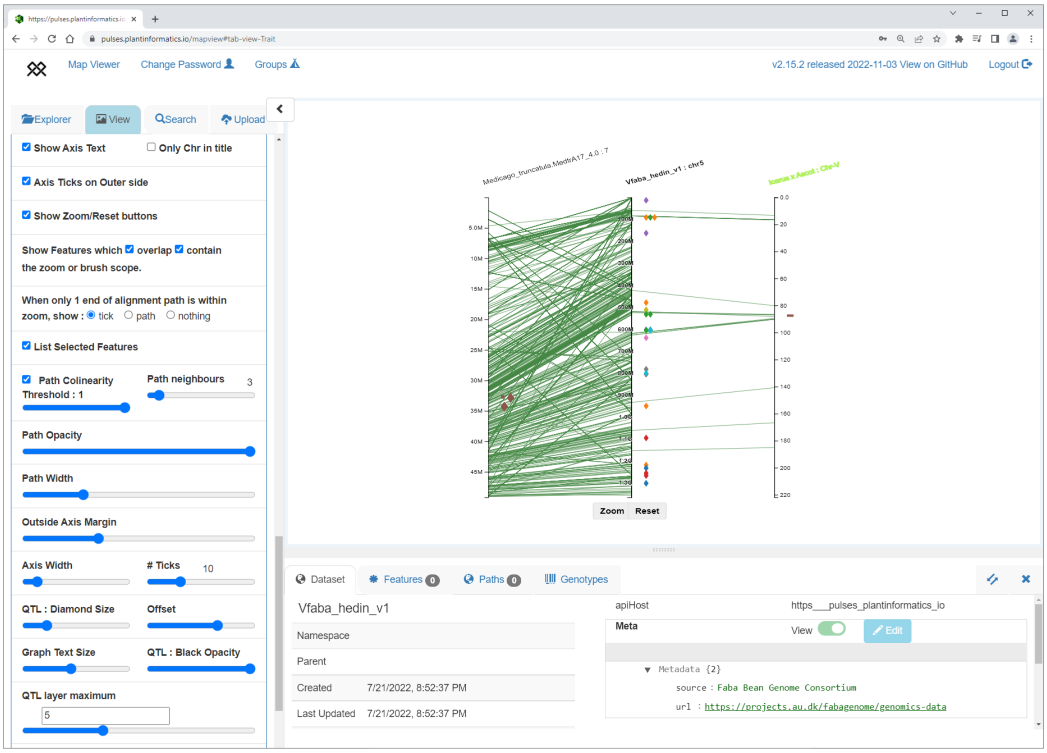

Reduce Path Opacity to reduce the opacity of the paths and the Path Collinearity Threshold to remove some of the cross-over paths.

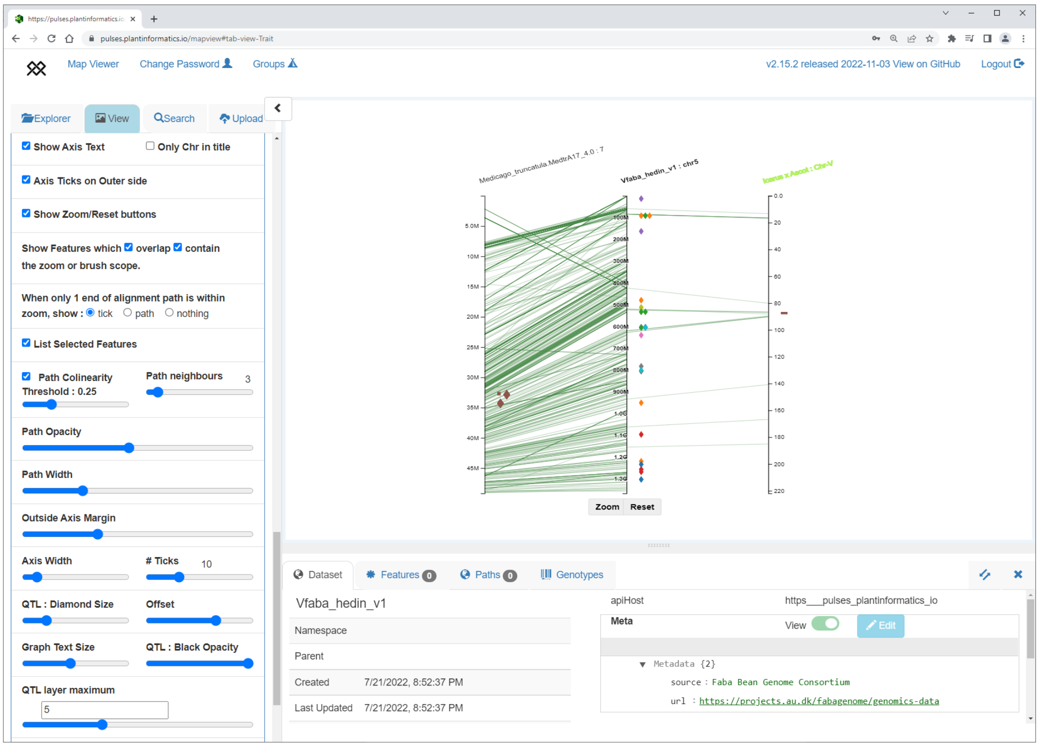

8. Zoom in to regions of interest

Using the brush operation described in step 5 above, select the regions of interest around the QTLs loaded.

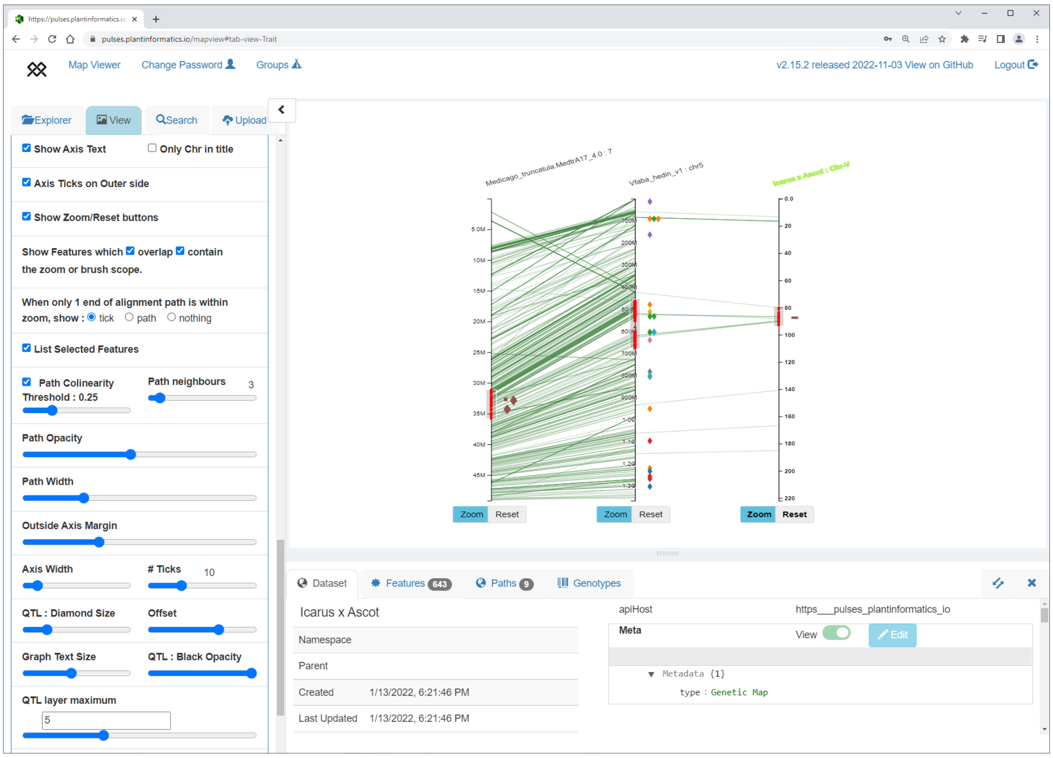

Zoom in to the brushed regions.

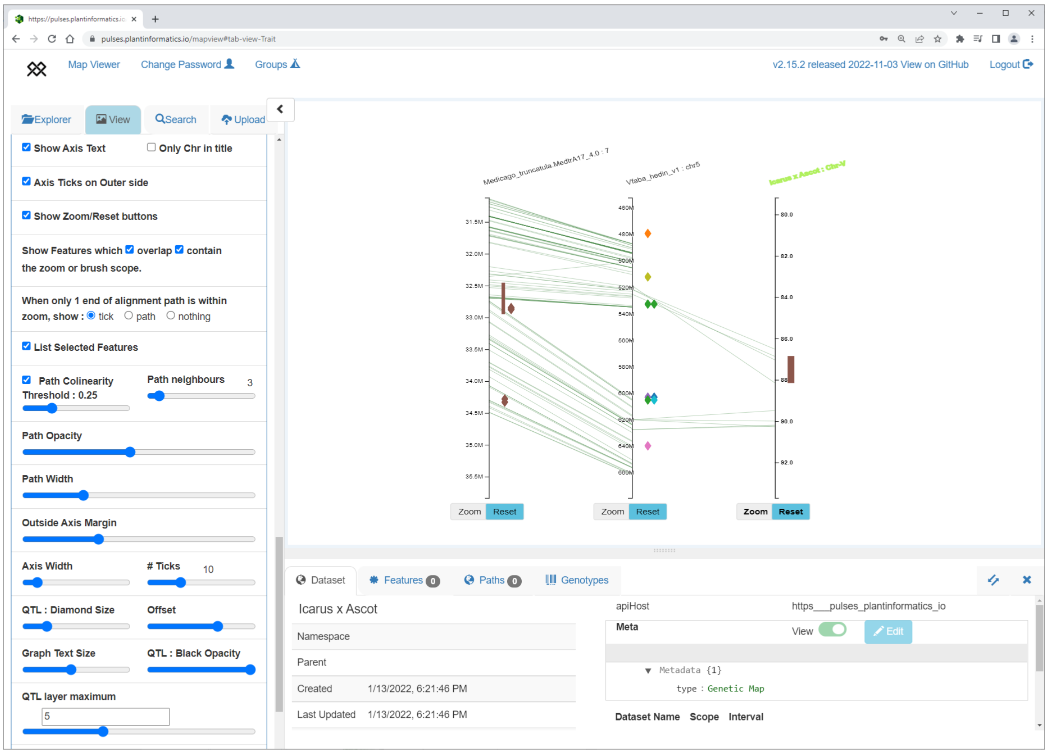

9. Visualise features in a table

Once a region of interest has been brushed, click the Features tab in the bottom panel.

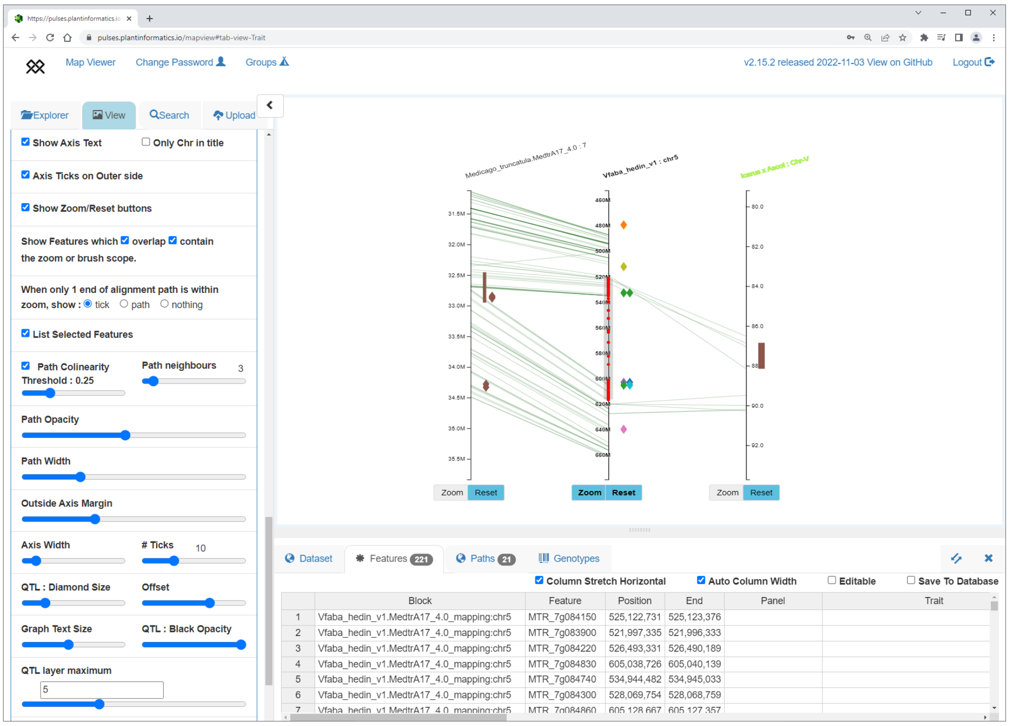

The displayed table shows all features in the brushed region (based on the datasets loaded in step 2 above).

10. Explore candidate genes in brushed region

Click the Explorer tab on the left panel and add the dataset Vfaba_hedin_v1.annotation – chr5.

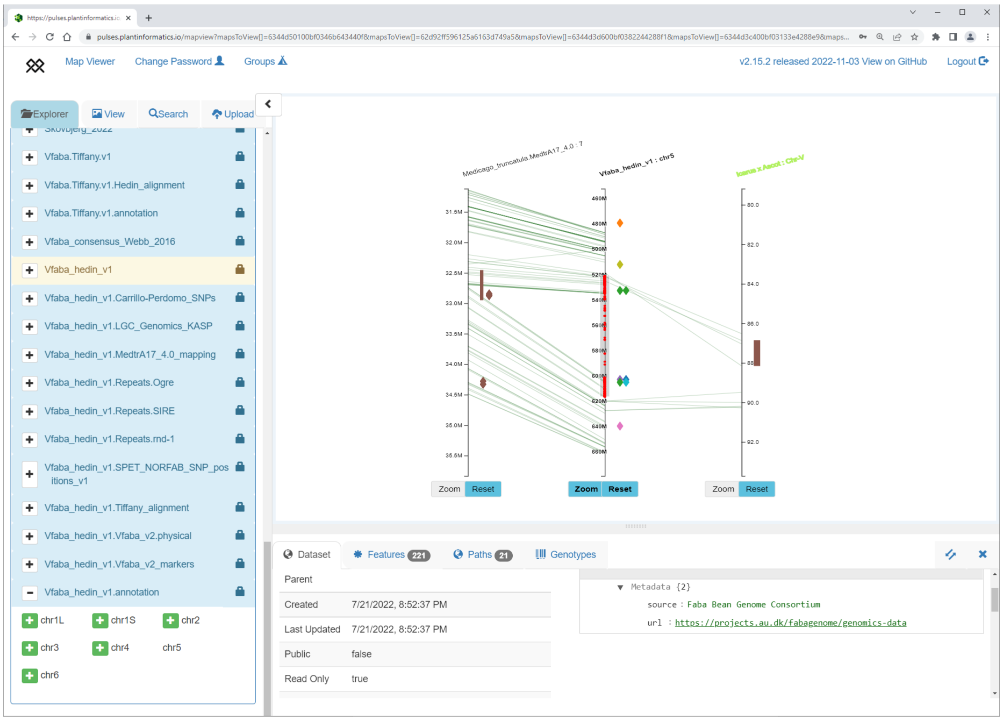

Click on the **Vfaba_hedin_v1 : chr5** text at the top of the axis to open the axis menu.

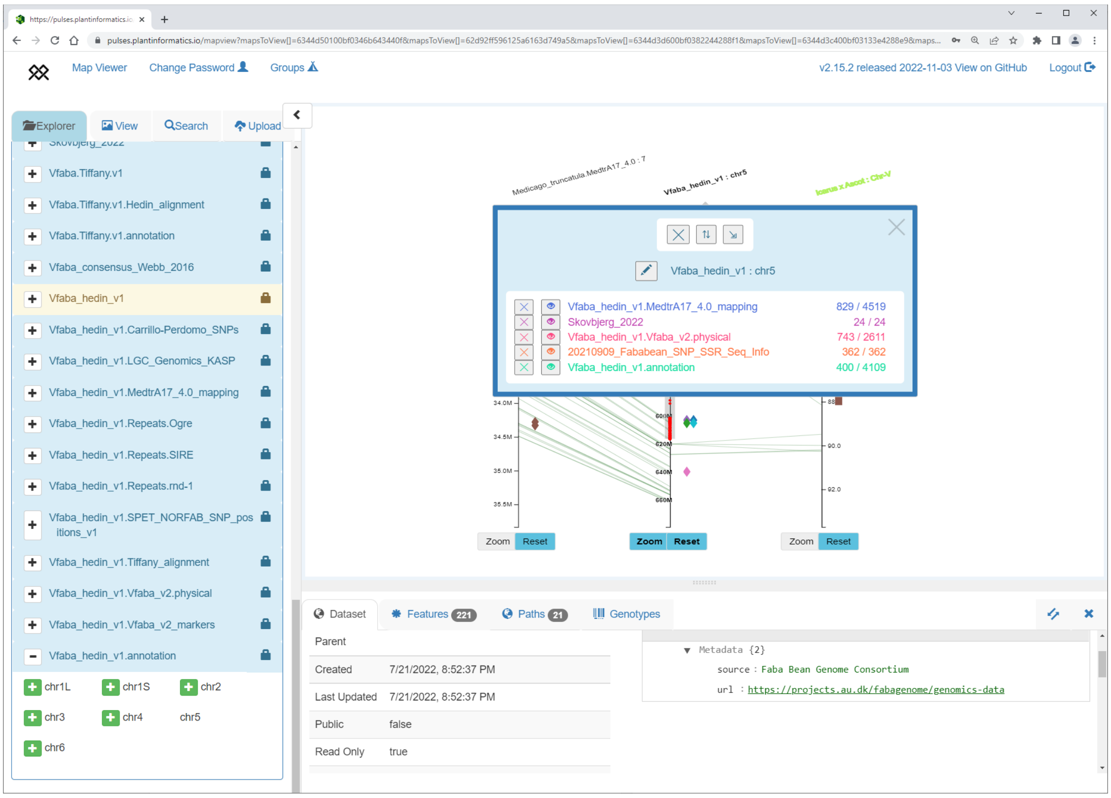

Click the icon with arrow pointing to the bottom right to split the axis.

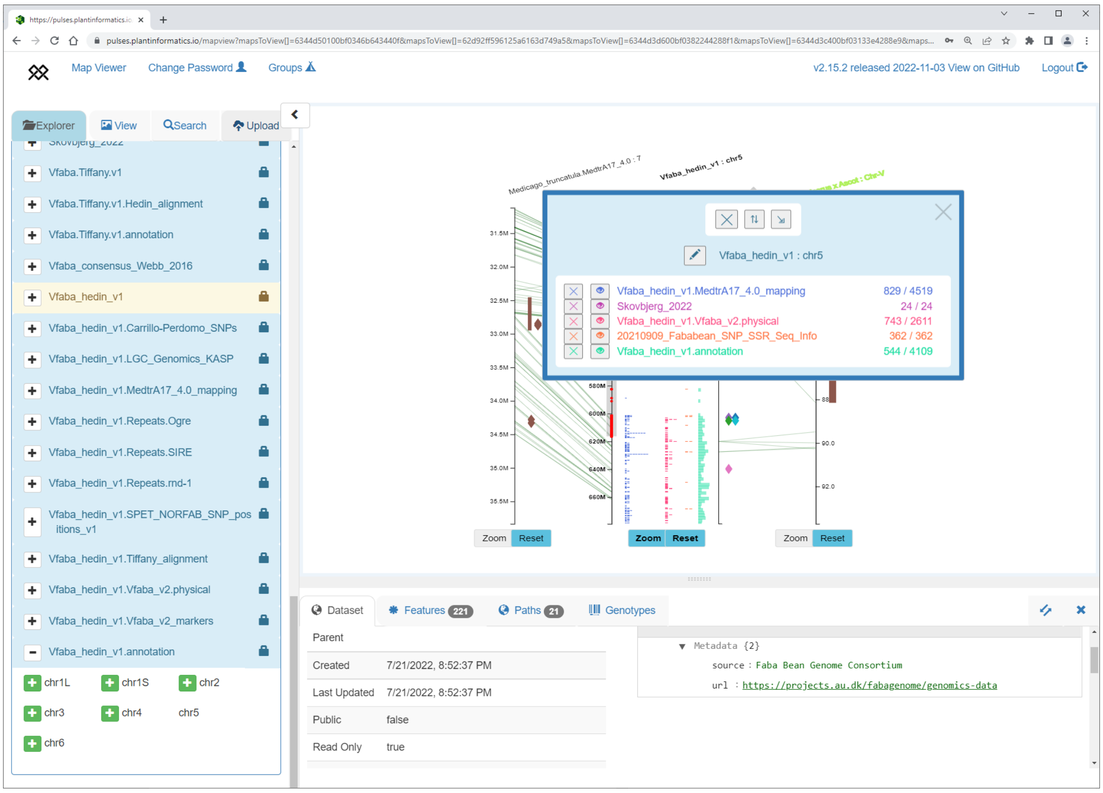

The split axis now displays the loaded datasets as individual features, or histograms if too many to draw. Zooming in will progressively re-draw the features.

Click the cross in the top right of the axis menu to close it. Now brush a region of interest and explore the Features table by clicking the Features tab in the bottom panel.

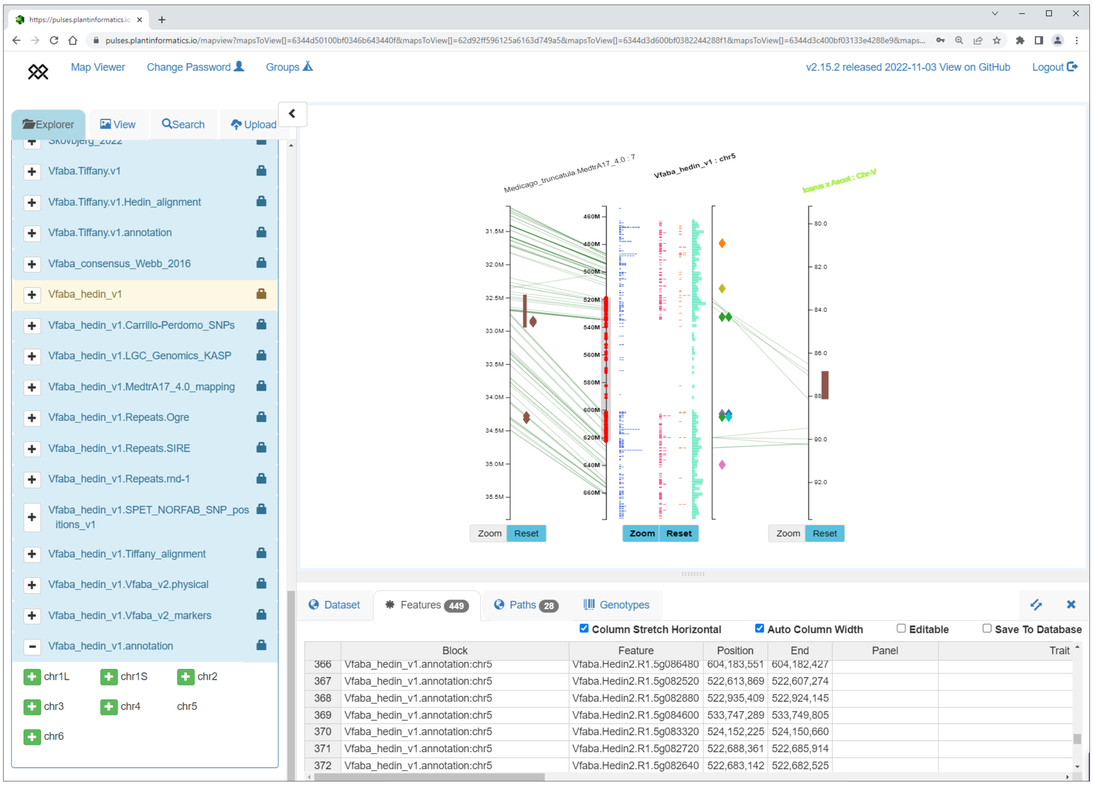

Supplement: Supplementary file 22 — Supplementary File 9: Raw Phenotype Scores from Field Trials (PDF 7208 KB) [file 122_2023_4360_MOESM22_ESM.pdf]
