# Supplementary material for: Genetic analysis of global faba bean diversity, agronomic traits and selection signatures
Source: Theor Appl Genet. 2023 Apr 19;136(5):114. doi: 10.1007/s00122-023-04360-8 (PMC10115707; doi:10.1007/s00122-023-04360-8)
Supplement: Supplementary file 24 — Supplementary file24 (DOCX 13 KB) [file 122_2023_4360_MOESM24_ESM.docx]

**Supplementary Tables**

**Supplementary Table 1. Management of trials.**

| **Treatment** | **Product** | **Applied amount** | **Date (DAY/MONTH/YEAR)** |
| --- | --- | --- | --- |
| **Nordic Seed, Dyngby, 2020 (Dyn20)** |  |  |  |
| Fertilizer | 0-8-23 | 300 kg/ha | 4/7/2020 |
| Herbicide | Novitron DAM TEC | 1.8 kg/ha | 4/8/2020 |
| Herbicide | Renol | 0.20 L/ha | 5/5/2020 |
| Herbicide | Fighter 480 | 1 /ha | 5/5/2020 |
| **Sejet, Horsens, 2020 (Sej20)** |  |  |  |
| Herbicide | Fighter 480 | 1.7 kg/ha | 5/7/2020 |
| Herbicide | Stomp SC | 1.4 kg/ha | 5/7/2020 |
| Insecticide | Karate 2.5 WG | 5 g/ha | 5/7/2020 |
| Herbicide | Agil | 5 g/ha | 5/7/2020 |
| Insecticide | MAVRIK VITA | 48 g/ha | 6/2/2020 |
| **Nordic Seed, Dyngby, 2021 (Dyn21)** |  |  |  |
| Fertilizer | 0-8-23 Mg 4S | 330 kg/ha | 2/4/2021 |
| Herbicide | Roundup PowerMax | 0.5 kg/ha | 10/4/2021 |
| Herbicide | Noviton DAM TEC | 1.8 kg/ha | 10/4/2021 |
| Insectiside | Lamdex | 0.20 kg/ha | 7/6/2021 |
| Insectiside | Pirimor 500 WG | 0.20 kg/ha | 18/06/2021 |
| **Sejet, Horsens, 2021 (Sej21)** |  |  |  |
| Herbicide | FIGHTER 480 - Bentazon | 1.0 l/ha | 19/05/2021 |
| Herbicide | STOMP SC - pendimethalin | 1.0 l/ha | 19/05/2021 |
| Insecticide | LAMDEX - lambda-cyhalothrin | 0.2 kg/ha | 19/05/2021 |
| Herbicide | FOCUS ULTRA - cycloxydim | 2.0 l/ha | 01/06/2021 |
